# Genomic Analysis Points to Multiple Genetic Mechanisms for Non-Transformable *Campylobacter jejuni* ST-50

**DOI:** 10.3390/microorganisms12020327

**Published:** 2024-02-04

**Authors:** Craig T. Parker, David A. Villafuerte, William G. Miller, Steven Huynh, Mary H. Chapman, Zahra Hanafy, James H. Jackson, Morgan A. Miller, Sophia Kathariou

**Affiliations:** 1 Produce Safety and Microbiology Research Unit, Agricultural Research Service, United States Department of Agriculture, Albany, CA 94710, USA; william.miller@usda.gov (W.G.M.);; 2 Department of Food, Nutrition and Bioprocessing Sciences, North Carolina State University, Raleigh, NC 27695, USA; david.a.villafuerte@outlook.com (D.A.V.); zahra.elsayed20@gmail.com (Z.H.); jjackson0195@gmail.com (J.H.J.III); mmiller1037@gmail.com (M.A.M.)

**Keywords:** *Campylobacter*, transformation, antimicrobial resistance

## Abstract

*Campylobacter jejuni* and *Campylobacter coli* are well known for their natural competence, i.e., their capacity for the uptake of naked DNA with subsequent transformation. This study identifies non-transformable *C. jejuni* and *C. coli* strains from domestic animals and employs genomic analysis to investigate the strain genotypes and their associated genetic mechanisms. The results reveal genetic associations leading to a non-transformable state, including functional DNase genes from bacteriophages and mutations within the *cts*-encoded DNA-uptake system, which impact the initial steps of the DNA uptake during natural transformation. Interestingly, all 38 tested *C. jejuni* ST-50 strains from the United States exhibit a high prevalence of non-transformability, and the strains harbor a variety of these genetic markers. This research emphasizes the role of these genetic markers in hindering the transfer of antimicrobial resistance (AMR) determinants, providing valuable insights into the genetic diversity of *Campylobacter*. As ST-50 is a major clone of *C.* jejuni globally, we additionally determined the prevalence of the genetic markers for non-transformability among *C. jejuni* ST-50 from different regions of the world, revealing distinct patterns of evolution and a strong selective pressure on the loss of competence in ST-50 strains, particularly in the agricultural environment in the United States. Our findings contribute to a comprehensive understanding of genetic exchange mechanisms within *Campylobacter* strains, and their implications for antimicrobial resistance dissemination and evolutionary pathways within specific lineages.

## 1. Introduction

*Campylobacter jejuni* and *Campylobacter coli* are Gram-negative, obligately microaerophilic, spiral-shaped and highly motile bacteria that frequently colonize poultry, cattle, swine and other animals worldwide [1]. Known for being the most common causative agent of bacterial gastroenteritis and the leading bacterial antecedent of the severe autoimmune complication Guillain-Barré syndrome [2,3,4], *C. jejuni* is an almost ubiquitous gastrointestinal pathogen. While most cases of campylobacteriosis in healthy individuals are self-resolving, immunocompromised patients may require antibiotics to prevent recurrent infections or the further progression of disease [5]. However, the increased incidences of antimicrobial resistance (AMR) in *C. jejuni* and *C. coli* have made it harder to treat known infections, and thereby cause an increased threat to public health [6,7].

*C. jejuni* and *C. coli* are genetically diverse, with over 12,000 sequence types (STs) in the PubMLST database [8]. *C. jejuni* and *C. coli* multilocus sequence typing (MLST), based on the alleles of seven housekeeping genes, is used to genotypically classify strains and strain lineages [9]. Indeed, MLST has become an important source-attribution tool for the epidemiology of *Campylobacter* [10,11].

The natural competence of *C. jejuni* and *C. coli*, i.e., the ability to undergo transformation via the uptake of naked DNA from its environment, plays an important role in their pronounced genetic diversity and horizonal acquisition of AMR determinants [6,12,13,14,15,16]. However, several studies reveal a variability in the transformation capacity among *Campylobacter* strains [16,17,18,19]. Notably, in previous studies from our laboratory, *C. coli* strains from turkeys were significantly more prone to be transformed to erythromycin resistance than *C. coli* from swine, and certain *C. coli* strains appeared completely unable to acquire either erythromycin or nalidixic acid resistance via transformation [20]. Inability to undergo transformation may reflect either the absence of determinants required for transformation [21] or the presence of impediments to transformation, such as restriction/modification systems, DNases encoded by integrated elements, and other barriers to gene flow [12,13,17,19]. Several genes have been shown to affect natural competence. The *Campylobacter cts* genes, which encode a DNA uptake system that is similar to type II secretion systems, have been shown to be essential for competence [21]. Additionally, nucleases encoded by *Campylobacter* bacteriophages have been identified as key players in preventing natural competence [17,19].

Despite the importance of understanding competence variation, investigations related to the transformation-mediated transfer of AMR genes have predominantly involved a limited number of extensively characterized strains [17,18,21,22,23]. In particular, studies that reported a variation in competence of *C. jejuni* and *C. coli* largely lack cross-referenceable information on the genotypes or clonal groups of strains that appear unable to be transformed [16,17,18,20]. Bridging this knowledge gap is important to further elucidate the role of competence in *C. jejuni* and *C. coli* in shaping the incidence of AMR and overall genomic diversity in different clonal groups.

In this study, we investigated the transformability of a panel of *C. jejuni* and *C. coli* strains isolated from domestic food animals in the in the United States (U.S.). From this strain panel, we identified several non-transformable *C. jejuni* and *C. coli* strains. Strikingly, we found that all the *C. jejuni* ST-50 strains in this strain panel were non-transformable. By analyzing the genomic sequences of these strains, we identified at least two potential mechanisms leading to non-transformability: (1) a defective *cts*-encoded DNA uptake system, or (2) the presence of a prophage-encoded nuclease. Evaluating *C. jejuni* ST-50 strains globally, we found that European and North American strains possessed considerably different prevalence of *cts* mutations, but comparable levels of phage-encoded DNases. Finally, our phylogenetic analysis points to the evolution of a clade of ST-50 strains throughout the U.S. which harbor *ctsD* and *ctsE* double mutations.

## 2. Materials and Methods

### 2.1. Bacterial Strains and Growth Conditions

The *C. jejuni* and *C. coli* strains investigated for transformability in this study are listed in Table S1. All strains with the FSIS designation were kindly provided by Dr. Glenn Tillman and Mustafa Simmons, United States Department of Agriculture (USDA), Food Safety Inspection Service (FSIS). The strains were chosen to be from diverse food animal hosts and to lack resistance to multiple antimicrobials. Several of the strains lacked known AMR determinants and were considered pan-susceptible, while some were resistant to one or maximally two antimicrobial classes (Table S1). The strains were grown microaerobically at 42 °C on Mueller–Hinton agar (MHA), consisting of Mueller–Hinton broth (MHB) (Becton, Dickinson and Co., Franklin Lakes, NJ, USA) amended with 1.2% agar. Microaerobic conditions were established using a Bactrox Hypoxia Chamber (Shel Lab, Cornelius, OR, USA).

### 2.2. Determination of the Transformation and Mutation Frequencies

The transformation assays were determined as previously described [24]. Briefly, DNA was extracted from donor strains *C. jejuni* 14980A and *C. coli* 14983A using the Qiagen DNeasy Blood and Tissue Kit (Qiagen, Redwood City, CA, USA), and was used for transformation of *C. jejuni* and *C. coli*, respectively, to nalidixic acid resistance (Nal^R^), or to streptomycin resistance (Str^R^) when the recipient was already Nal^R^ [25]. The transformation recipients were grown on MHA overnight. A colony from the MHA plate was suspended in 1 mL MHB, and 25 µL of this suspension was spotted onto a new MHA plate. Donor DNA (approx. 700 ng) was added to each recipient suspension spot, and the spots were allowed to dry in a NuAire biosafety cabinet (Class 2, type A/B3; NuAire, Plymouth, MN, USA) prior to being incubated microaerobically at 42 °C overnight. For enumerations of transformants, each spot was suspended in 500 µL MHB, diluted as needed, and the dilutions spotted (10 µL, in duplicate) or spread-plated on MHA were amended with either nalidixic acid (32 µg/mL) or streptomycin (64 µg/mL). Colonies were enumerated following incubation at 42 °C microaerobically for 36–48 h. The total CFUs of the recipient at the end of the transformation period were also enumerated by plating the dilutions on MHA without nalidixic acid or streptomycin. The transformation frequency was determined as the ratio of total CFUs on MHA with the appropriate antibiotic over the total CFUs on MHA without any antibiotics. To determine the spontaneous mutation frequency of resistance to the relevant antimicrobial, cell suspensions from recipient spots with no added DNA were diluted and plated on MHA with either nalidixic acid (32 µg/mL) or streptomycin (64 µg/mL). The mutation frequency was calculated as the ratio of total CFUs in the control spot without added DNA on MHA supplemented with the appropriate antibiotic over the total CFUs on MHA with no antibiotic. All transformation assays included as a control *C. jejuni Cj0264c*::Cm, a derivative of *C. jejuni* NCTC 11168, that was confirmed to be readily transformable to Nal^R^ and Str^R^ using DNA from the donors employed here [24].

### 2.3. Bacterial Strain Genomic Sequences

The genomes for the FSIS strains in Table S1 were obtained from BioProject PRJNA292668, available at NCBI (https://www.ncbi.nlm.nih.gov/bioproject/PRJNA292668/, accessed on 2 January 2024); the associated GenBank assembly accession numbers are also in Table S1. For additional genomic sequences obtained for this study, DNA was extracted from *C. jejuni* strains RM3405, RM3412, RM5146, RM5148, RM5149 and RM5156, as described previously [26]. DNA sequencing libraries for Illumina MiSeq were prepared using an Illumina DNA Prep Tagmentation kit (Illumina, San Diego, CA, USA), following the manufacturer’s instructions, except for changes that increased library insert size. To do this, the library preparation was done to a median of 631 bp and a size range between ~375 and1100 bp by decreasing the 1st and 2nd volume of Sample Purification Beads to 40 μL and 11 μL, respectively. This modification resulted in larger inserts compared to the mostly <300 bp inserts obtained using the manufacturer’s protocol. The final elution volume of the libraries was in 10 μL of the Illumina resuspension buffer. Illumina-DNA/RNA UD Indexes Plate A, B, C and D dual index adapters were ordered from Integrated DNA Technologies (Coralville, IA, USA) and used at a 1 μM final concentration. Instead of pooling equal volumes, individual libraries were quantified using the KAPA Library Quantification Kit (Roche, Indianapolis, IN, USA), since we found qPCR to be a more accurate quantification than using equal volume. Libraries were quantified in 10 μL volume reactions and 90 s annealing/extension PCR, and then pooled and normalized to 4 nM. The pooled libraries were re-quantified using ddPCR on a QX200 system (Bio-Rad, Hercules, CA, USA) using the Illumina TruSeq ddPCR Library Quantification Kit and following the manufacturer’s protocols. Libraries were sequenced using a MiSeq Reagent Kit v2 (500-cycles; Illumina) on a MiSeq instrument (Illumina) at 16 pM, following the manufacturer’s protocols. Genomes were assembled using a SPAdes assembler 3.15.5 within Geneious Prime v. 2023.1.2 (Biomatters, Ltd., Auckland, New Zealand). The GenBank accession numbers for these genome sequences are also listed in Table S1.

### 2.4. Determination of cts Operon, CJIEs and Associated DNase Genes

PubMLST (https://pubmlst.org/organisms/campylobacter-jejunicoli; accessed on 2 January 2024) was used to identify ST-50 strains with whole genome data. BLASTN analysis tools within Geneious Prime v. 2023.1.2 or PubMLST (https://pubmlst.org/organisms/campylobacter-jejunicoli; accessed on 2 January 2024) were used to screen for specific genomic regions within the genomes of the *C. jejuni* and *C. coli* strains described in Table S1 and all the ST-50 strains accessible in PubMLST (Table S2). The specific bait sequences for the *cts* genes [21] were from *C. jejuni* strain 14980A (GenBank accession: CP017029; locus tags: CJ14980A_1035, CJ14980A_1081, CJ14980A_1331 and CJ14980A_1454-CJ14980A_1459) or *C. coli* strain 14983A (GenBank accession: CP017025; locus tags: CC14983A_0294- CC14983A_0299, CC14983A_0414, CC14983A_0691 and CC14983A_0743) [25]. The specific bait sequences for three *C. jejuni*-integrated elements (CJIE1, CJIE2 and CJIE4) and their cognate nuclease genes (locus tags CJE0256, CJE0566 and CJE1444 from CJIE1, CJIE2 and CJIE4, respectively) [17,19] were from the *C. jejuni* strain RM1221 (GenBank accession: CP000025) [27]. The genes from each strain were downloaded from PubMLST for the determination of complete open reading frames, nonsense mutations and frameshift mutations. Any genes with contig ends within the reading frame were not characterized further.

For the Australian *C. jejuni* ST-50 strains [28], SRA reads (Table S3) were downloaded from NCBI and assembled using SPAdes assembler 3.15.5 within Geneious Prime v. 2023.1.2 (Biomatters, Ltd., Auckland, New Zealand). The *cts* genes and the CJIE1, CJIE2 and CJIE4 nuclease genes mentioned above were identified using the annotation function within Geneious Prime v. 2023.1.2.

### 2.5. DNase Detection

Cultures were started on anaerobe basal agar plates (Oxoid, Thermo Fisher Scientific, Waltham, MA, USA), amended with 5% laked horse blood (Innovative Research, Novi, MI, USA) and incubated for 24–48 h at 37 °C microaerobically (using a proprietary blend of gases (Bioblend, Linde USA, Danbury, CT, USA) consisting of 10% CO_2_, 10% H_2_, and 80% N_2_ to adjust the final concentration of O_2_ to 3–5%) in an Oxoid 3.5 L AnaeroJar (Oxoid, Thermo Fisher Scientific, Waltham, MA, USA). Methyl green-DNA plates were prepared by adding methyl green dye (Thermo Fisher Scientific; final concentration = 50 mg L^−1^) and fish sperm DNA (Fisher Scientific; final concentration 2 g/L) to blood-free anaerobe basal agar. Cultures were spotted thickly onto methyl green-DNA media and incubated in flat = bottomed freezer bags (Ziploc, S.C. Johnson, Racine, WI, USA) at the same O_2_ level. The presence of DNase was indicated by the appearance of a clear halo around the culture.

### 2.6. Determination of Phylogeny

A total of 63 ST-50 strains were examined phylogenetically (Table S4). The genomes of six strains (RM3405, RM3412, RM5146, RM5148, RM5149 and RM5156) sequenced for this study were uploaded to PubMLST and sequence typed to the seven gene multilocus sequence type (MLST) and the core genome MLST in silico [9]. Genomes for 22 FSIS strains in Table S1 were available in PubMLST, and 41 additional ST-50 genomes in the PubMLST database were used for comparison. A multisequence alignment was created from the concatenated gene sequences of all core genes (found in 100% of isolates) shared with NCTC 11168 (AL111168) using MAFFT [29]. Dendrograms were created using the neighbor-joining method.

Additionally, the 63 ST-50 strains (Table S4) were compared phylogenetically to the Australian ST-50 strains (Table S3). The assembled genomes of the 23 Australian ST-50 strains (Table S3) were uploaded to PubMLST. A neighbor-joining dendrogram was created using the Interactive Tree of Life (iTOL) plugin within PubMLST using the concatenated nucleotide sequences of the *C. jejuni*/*C. coli* cgMLSTv2 datasets for each genome.

## 3. Results

### 3.1. Transformation Frequencies of C. jejuni and C. coli

An initial analysis of a panel of 25 sequenced FSIS *C. jejuni* and *C. coli* strains with few or no AMR determinants and derived from poultry processing plants in the United States (US) identified 13 that appeared to be non-transformable to Nal^R^, including both available *C. jejuni* strains of ST-50. To determine the prevalence of non-transformability in ST-50, additional ST-50 strains from diverse food animals were requested from the FSIS. Interestingly, the testing of 36 additional FSIS *C. jejuni* ST-50 strains from poultry, cattle and swine in the US revealed that all were similarly non-transformable (Table 1). Altogether, we examined the natural transformability of 73 *Campylobacter* isolated from chickens, cattle, and sheep across multiple locations in the U.S. by the USDA and FSIS from 2016 to 2019 (Table S1). This panel of strains consisted of 54 *C. jejuni* strains, representing six MLST sequence types and 19 *C. coli* strains, representing 5 sequence types (Table 1).

Each of the *C. jejuni* and *C. coli* strains were used as recipient for donor DNA from the Nal^R^, Str^R^ strains *C. jejuni* 14980A and *C. coli* 14983A, respectively. Certain recipient strains were already Nal^R^, and these were only transformed to Str^R^. Since Nal^R^ and Str^R^ strains can also arise by spontaneous point mutations within *gyrA* and *rpsL*, respectively, we performed control transformations without any donor DNA added to determine the relevant mutation frequencies (Table 1). The strains were determined to be transformable when the transformation frequency was three logs or higher above the mutation frequency. There were 9 of 18 *C. coli* strains that were not transformable and 46 of 55 *C. jejuni* strains that were not transformable, including all 38 ST-50 strains. Since 100% of the *C. jejuni* ST-50 strains that we examined from the U.S. were not transformable, we investigated the natural competence of six other ST-50 strains available in our collection and isolated outside of the U.S, including four from Italy and two from Canada (Table S1). Among these six ST-50 strains, one (RM5156, from Italy) was identified as transformable.

### 3.2. Multiple Mechanisms for Non-Transformability among ST-50 Strains

To determine whether the lack of transformability reflected the presence of specific restriction/modification systems harbored by the recipient strains, genomic DNA was extracted from spontaneous Nal^R^ mutants identified in the control transformation assays. This DNA was then utilized as donor DNA for transformation of the corresponding parental, nalidixic acid-susceptible recipient strain to Nal^R^. Without exception, the recipient stains remained non-transformable. 

To understand the mechanism(s) for why some strains were not naturally transformable, we queried the whole genome sequences of these strains against loci that are known to affect natural competence, specifically mutations within *cts* genes and the presence of bacteriophage with nuclease genes (Table 2). All of the transformable *C. jejuni* and *C. coli* strains in the panel possessed wild-type *cts* genes. Among the 46 *C. jejuni* strains that were not transformable, 19 strains had nonsense or frameshift mutations within *cts* genes that would prematurely disrupt the coding sequence and should disrupt the Cts DNA uptake system (Table 2). From these 19 strains, 18 were ST-50 and 15 of these ST-50 strains possessed the same double mutations within *ctsD* and *ctsE*. Strain RM3405 from Canada had nonsense mutations in *ctsR* and *ctsF*.

There were also 27 non-transformable *C. jejuni* strains that possessed wild-type *cts* genes, suggesting multiple mechanisms for the non-transformable phenotype among *C. jejuni* strains. Among these 27 *C. jejuni* strains, all but one (RM3412) possessed at least one bacteriophage with a nuclease gene. Indeed, 26 ST-50 strains possessed a Mu-like bacteriophage that carried *dns,* which encodes an extracellular DNase. In five ST-50 strains (FSIS12030287, FSIS12030565, FSIS12031145, FSIS1608758, and FSIS1609357) and one ST-353 strain (FSIS11810577), *dns* possessed frameshift mutations. However, the six strains mentioned above also possessed double mutations within *ctsD* and *ctsE* and were not transformable, while the ST-353 strain with a *dns* nonsense mutation had wild-type *cts* genes and was transformable (Table 1 and Table 2). The four ST-50 strains from Italy had wild-type *cts* genes and Mu-like bacteriophages. Among these, the one transformable strain (RM5156) harbored a nonsense mutation in *dns*. Bacteriophages harboring nuclease genes similar to CJE0566 and CJE1444, defined here as *dns2* and *dns3*, respectively (Table 2), were also harbored by *C. jejuni* strains that were not naturally transformable. Finally, RM3412, a ST-50 strain from Canada, lacked the discernable genetic content that leads to non-transformability (Table 1 and Table 2).

In the case of *C. coli*, all non-transformable strains possessed functional *cts* genes. Eleven *C. coli* strains were ST-3262 and eight of these were not naturally transformable. All of the ST-3262 strains harbored a Mu-like bacteriophage and CJIE2-like bacteriophage. However, the Mu-like bacteriophage did not harbor *dns*, while the CJIE2-like bacteriophage did possess *dns2*. The three transformable ST-3262 strains had nonsense mutations within *dns2* of the CJIE2-like bacteriophage (Table 2).

### 3.3. Global Examination of the ST-50 Non-Transformability Genotype

With 42/43 non-transformable *C. jejuni* ST-50 strains having a likely genetic basis for the phenotype, our investigation extended to the analysis of over 2200 *C. jejuni* ST-50 genomes in the PubMLST database. The search focused on identifying *cts* mutations or the presence of bacteriophage-encoded DNase genes. The criteria for *cts* genes involved assessing wild-type and loss-of-function mutational states requiring the presence of the complete *ctsRDPXEF* operon on a single contig and the complete coding sequences of *ctsG*, *ctsT*, and *ctsW*. Similarly, for bacteriophage-encoded DNase assessments, the criteria included the complete coding sequences of *dns* (the Mu-like phage DNase gene), *dns2* (CJIE2-like) and *dns3* (CJIE4-like) needed to be on single contigs with frameshift and nonsense mutations indicating a nonfunctional gene. Missense mutations were disregarded, as their functional outcome was difficult to assess and beyond the scope of this study.

Table 3 illustrates that 37.8% of ST-50 strains from North America possessed *cts* gene mutations, while only 3.3% of European ST-50 strains exhibited such mutations. The presence of Mu-like bacteriophage *dns* was determined with 53.3% and 64.3% of strains possessing the gene in North America and Europe, respectively. The *dns2* and *dns3* genes from CJIE2 and CJIE4 were detected at lower levels than the Mu-like bacteriophage *dns* in both regions, with 30.3% in North America and 28.4% in Europe. Some strains featured multiple mechanisms for non-transformability, exemplified by strain FSIS1710700 with *ctsD ctsE* double mutations and *dns*, and strain FSIS1607146 with *dns* and *dns3* (Table 2). Calculations based on presence of *cts* mutations, *dns*, *dns2* and *dns3* in ST-50 strains from North America and Europe revealed an approximate percentage of non-naturally transformable ST-50 strains to be 91% in North America and 75% in Europe.

We also examined the genomes from 23 *C. jejuni* ST-50 isolated from chickens in Australia [28]. We assembled these genomes from the available SRA reads in NCBI, since they were not available in PubMLST (Table 1). We found *cts* mutations in 8 out of 23 strains (35%), a level similar to the prevalence of *cts* mutations in *C. jejuni* ST-50 in the U.S. (Table 3). In the Australian strains, loss-of-function mutations were exclusively in a single gene, *ctsE*. Phage-encoded DNase genes were also identified in these strains, with *dns* present in five of 23 (~22%) and *dns2* present in 12 out of 23 (~52%) (Supplementary Table S1). Overall, 17 out of the 23 (~74%) strains harbored genetic markers for non-transformability.

To assess whether the findings regarding non-transformability of *C. jejuni* ST-50 were unique to this clone, we examined the genetic markers for transformability for over 800 *C. jejuni* ST-8 strains to determine if non-transformability was common to *C. jejuni* strains in North America. *C. jejuni* ST-8 strains are in the same clonal complex, CC-21, as ST-50, and they are predominantly distributed in North America. For *cts* mutations, only 641 genome sequences harbored the operon *ctsRDPXEF* on a single contig. We identified that 49 of these 641 (~8%) harbored loss-of-function *cts* mutations. Phage-encoded DNase genes were also identified in these strains, with *dns* present in 62 out of 809 strains (~8%) and *dns2* or *dns3* present in 237 out of 809 (~29%) (Table 3). Overall, there were 641 ST-8 strains that we could examine where all genetic markers for transformability could be assessed. Collectively, our analysis indicated that 218 (~34%) of these 641 ST-8 strains harbored genetic markers for non-transformability. (Table S4).

### 3.4. Evidence of Extracellular DNase from dns Positive Strains

**Figure 1:**
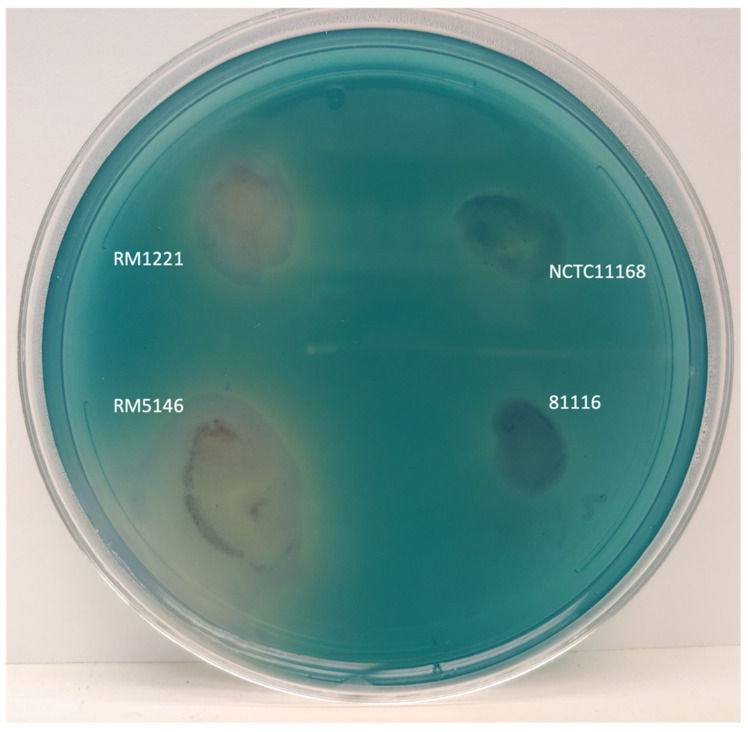
Extracellular DNase activity of *C. jejuni*. Methyl green-DNA media were inoculated with four different strains *C. jejuni*: RM1221 (*dns*^+^), NCTC 11168 (*dns*^−^), RM5146 (*dns*^+^), and 81116 (*dns*^−^). The presence of DNase was indicated by the appearance of a clear halo beneath and around the culture.

Four ST-50 strains (RM5146, RM5148, RM5149 and RM5156) possessing Mu-like bacteriophages with *dns* were examined for extracellular DNase activity. Three strains (RM5146, RM5148 and RM5149) were non-transformable and had wild-type *dns* genes. All three strains showed DNase activity. Figure 1 illustrates the DNase activity of RM5146 compared to *C. jejuni* strain RM1221, which also possesses *dns*, and strains NCTC 11168 and 81116 that do not have the *dns* gene. The DNase activity of strain RM5146 appeared noticeably higher than strain RM1221, while no activity was detected for strains NCTC 11168 and 81116 (Figure 1). The *Campylobacter jejuni* strain RM5156 with mutated *dns* showed no DNase activity.

### 3.5. Phylogenetic Comparison of North American and European ST-50 Strains

**Figure 2:**
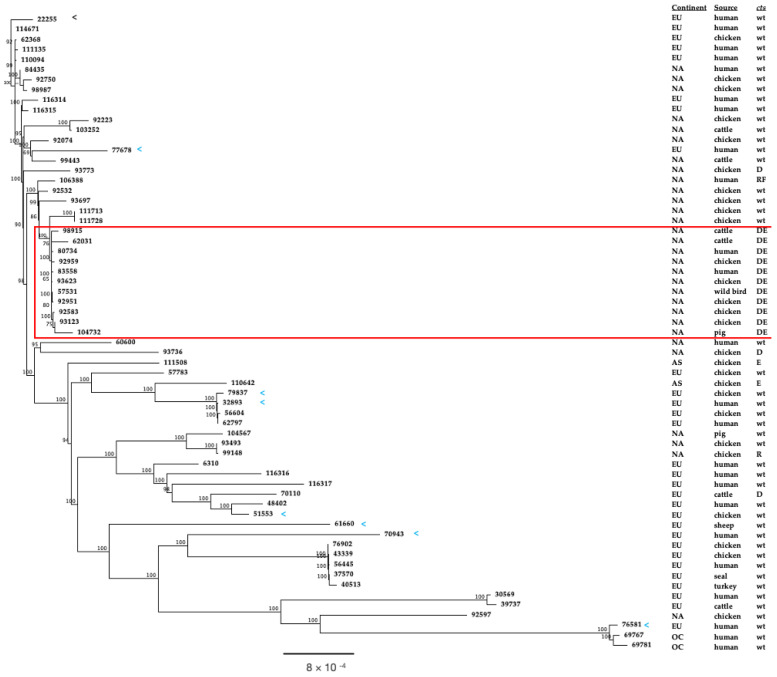
Neighbor-joining dendrogram of *C. jejuni* ST-50 strains. The neighbor-joining dendrogram of 63 *C. jejuni* ST-50 strains (Supplementary Table S1) was constructed from the concatenated, aligned core genes. Bootstrap values of ≥50%, generated from 500 replicates, are shown at the nodes. Leaves are labeled with PubMLST IDs, and the strain information can be found in Table S5. Blue arrowheads indicate strains that lacked the investigated non-transformability genetic markers. Metadata related to continent (North America: NA, Europe: EU, Asia: AS, and Oceania: OC) source; *cts* mutations are adjacent to each leaf. The red rectangle indicates the cluster of strains that share the *ctsD* and *ctsE* mutations.

The phylogeny of 63 ST-50 strains (Table S5) was inferred from their core genomes ((genes present in all strains), using the neighbor-joining method to create a dendrogram (Figure 2). The strain collection included 30 from North America, 29 from Europe, and two each from Asia and Oceania. These strains were isolated from a variety of sources including humans, chickens, pigs, cattle and sheep. The dendrogram shows that North American ST-50 strains mostly form clusters with other North American ST-50 strains. Conspicuously, there was a large cluster of strains from the U.S. that all possessed double mutations within *ctsD* and *ctsE* (Figure 2, red-boxed cluster). The strains in this cluster were from human clinical and multiple animal sources (chicken, cattle, pig and wild bird). Moreover, the *C. jejuni* ST-50 strains possessing the *ctsD* and *ctsE* mutations were not confined to a particular region within the U.S., but were isolated from animals from the southeastern seaboard to the west coast of the U.S. (Table S5). Among the European ST-50 strains, 8 (PubMLST IDs 22255, 32893, 51553, 61660, 70943, 76581, 77678, 79837) possessed wild-type *cts* genes and no *dns*, *dns2*, or *dns3*. The extent to which these strains were non-transformable remains unknown, as they were not available for experimental assessments. These eight strains did not cluster together, but were dispersed throughout the dendrogram (Figure 2, see arrows, <). Finally, we performed a phylogenic analysis that included the 23 Australian ST-50 strains that were previously shown to be phylogenetically distinct from most North American and European ST-50 strains [28] (Supplementary Table S1). This analysis verified that most Australian ST-50 strains form a distinct clade, with the inclusion of one strain from Europe (Figure S1).

## 4. Discussion

In this study, we identified both non-transformable *C. jejuni* and *C. coli* strains isolated from domestic food animals. We employed genomic analysis to understand the mechanisms associated with *Campylobacter* strains that are not naturally transformable. We have established the genetic associations leading to a non-transformable state, including the presence of functional DNase genes possessed by bacteriophages in both *C. jejuni* and *C. coli* strains, which have been previously observed in some *C. jejuni* strains [17,19]. Additionally, we identified *C. jejuni* strains possessing *cts* mutations that would disrupt the DNA uptake system involved in natural transformation [21,22].

An especially interesting and unexpected finding was the non-transformability of *C. jejuni* ST-50, a major, globally-distributed clone with intriguing phylogeny [28]. Among the *Campylobacter* strain panel investigated for transformation capacity, we identified that all but one (43/44) strains of *C. jejuni* ST-50 were non-transformable. The sole ST-50 strain that was found to be transformable was from Italy and did not harbor the established genetic markers. We further determined that globally >75% of approximately 2400 *C. jejuni* ST-50 strains from the PubMLST database would be expected to be non-transformable, as they possess the genetic markers associated with the non-transformable phenotype. The remaining ST-50 strains not harboring these genetic markers may be non-transformable via alternative mechanisms not investigated in this study, or they may be transformable. Thus, we defined the genetic markers for a non-transformable genotype among field isolates of *C. jejuni* ST-50 and other non-transformable field isolates of *C. jejuni* and *C. coli*, specifically the presence of functional bacteriophage DNase genes or mutations within *cts* genes.

These genetic markers harbored by non-transformable *C. jejuni* and *C. coli* strains are expected to have a negative impact on the initial steps of DNA uptake during natural transformation. The natural competence for transformation of *C. jejuni* with naked chromosomal DNA was first detailed in 1990 [16]. The identification of *cts* genes as encoding components of a DNA uptake apparatus was elucidated more than a decade later [21]. We identified nonsense and frameshift mutations in *ctsD*, *ctsE*, *ctsR* and *ctsF*. These mutations cause prematurely truncated components and result in a nonfunctional DNA uptake system, as previously observed following insertion mutations within the same genes [21,22]. The involvement of the bacteriophage DNases in natural competence was reported for three different bacteriophage families, CJIE-1-like bacteriophage (Mu-like phage), CJIE2-like bacteriophage and CJIE4-like bacteriophage [17,19]. We identified the non-transformable strains possessing *dns* from the *Campylobacter* Mu-like phage and *dns2* or *dns3* from CJIE2 and CJIE4 bacteriophages, respectively. Additionally, we observed that strains with loss-of-function mutations in *dns* and *dns2* were transformable, further supporting the role of *dns* and *dns2* in non-transformability. The impact of these DNases on natural transformation would be to degrade the naked donor DNA prior to its interaction with the DNA uptake system or once in the cytoplasm nd before recombination. We demonstrated that the strains possessing *dns* degraded extracellular DNA. Further research is needed to determine mechanisms via which *dns2* or *dns3* inhibit transformation.

The ability to acquire and recombine DNA between strains of *C. jejuni* and *C. coli* plays a significant role in the genetic diversity of *C. jejuni* and *C. coli*, including the movement of chromosomal AMR markers in these species [9,16,30]. Our study along with others [20,24] establishes that the transfer of AMR determinants through natural transformation is markedly more frequent, by several orders of magnitude, than the occurrence of mutations leading to resistance. Although most natural transformation studies are performed in vitro using purified donor DNA [16,20,24], it is noteworthy that natural transformation has been demonstrated in vivo within chickens and turkeys, which are prevalent hosts for this pathogen [31,32].

All 38 experimentally-investigated *C. jejuni* ST-50 strains isolated from domestic food animals by the U.S. Department of Agriculture Food Safety Inspection Service exhibited the non-transformable phenotype and corresponding associations with the *cts* and DNase non-transformability genetic markers. Strikingly, the non-transformable genotypes varied among the *C. jejuni* ST-50 strains that we investigated. Among the entire panel (n= 44, including 38 from the US and six from elsewhere) strains of *C. jejuni* ST-50 that were experimentally assessed for transformation, 9 harbored *cts* mutations only, 14 possessed a complete *dns* only, 5 had a complete *dns2* only, 2 harbored a complete *dns3* only, and 12 harbored multiple markers. One strain was non-transformable but lacked the investigated genetic markers. In this case, it is possible that a *cts* missense mutation impaired the DNA uptake system, or that the strain possesses a novel DNase that caused the non-transformable phenotype. Intriguingly, as indicated above several of these ST-50 strains possessed more than one genetic marker, i.e., *cts* mutations as well as a complete *dns*. 

Upon examining these non-transformable genotypes in a broader context within the global set of *C. jejuni* ST-50, a discernible pattern emerged. Specifically, *cts* loss-of-function mutations, particularly the *ctsD* and *ctsE* double mutations, appear to have evolved and dispersed throughout the agricultural environment within the U.S. These mutations seem to have occurred independent of the bacteriophage DNases, which are present at high levels in both North American and European *C. jejuni* ST-50 strains. Our phylogenetic results examining a global set *C. jejuni* ST-50 strains support the independent evolution of the *C. jejuni* ST-50 possessing the *ctsD* and *ctsE* double mutations in the U.S. This observation aligns with a study by Wallace et al. [28], who performed a similar phylogenetic analysis on 162 *C. jejuni* ST-50 isolates, comparing those from Australia with other parts of the world. Wallace et al. [28] highlighted the independent evolution of Australian ST-50 strains, which formed a distinct cluster from ST-50 isolates from Europe and North America. Inclusion of the Australian ST-50 strains not only verifies the findings of this previous study [28], but demonstrates that the evolution of strains in the U.S. and Australia is truly independent.

As mentioned above, we have identified that the genetic markers for non-transformability vary in occurrence. Notably, *cts* mutations were much more common in strains from North America and Australia (~37%) when compared to those from Europe (~3%). However, the prevalence of the different bacteriophages was comparable between North America and Europe, whereas the Mu-like phage was present in >50% of the ST-50 strains from the U.S. and Europe but in <25% of the ST-50 strains from Australia. Nevertheless, the overall prevalence of the non-transformable genotypes was ~75% in Europe and Australia and ~90% in North America. Finally, we determined that the prevalence of genetic markers associated with non-transformability within *C. jejuni* ST-8 strains was ~34%, demonstrating that non-transformability is not common to all *C. jejuni* strains in North America.

## 5. Conclusions

*Campylobacter* strains with reduced natural transformation frequencies were frequently observed in a panel of *C. jejuni* and *C. coli* strains from domestic food animals in the U.S and exhibited resistance to no or few antimicrobials (maximally two antimicrobial classes). Genomic characterization of the non-transformable strains allowed us to identify specific genomic markers/genotypes that likely accounted for non-transformability, including (1) loss-of-function mutations in the *cts* genes encoding a DNA uptake system, and/or (2) the presence of bacteriophage-encoded DNases. Notably, these non-transformability-associated genomic markers were detected, individually or in combination, in all but 1 of the 43 non-transformable *C. jejuni* ST-50 strains on our panel. Examining the prevalence of non-transformable genotypes of ST-50 strains from different regions of the world, we observed distinct patterns of evolution, particularly in the agricultural environment of the U.S. The findings suggest strong selection pressures for the evolution and maintenance of the non-transformable state in ST-50, a major, globally distributed clone of *C. jejuni*. The potential impacts of non-transformability on the natural fitness, and possibly the pathogenicity, of this clone remain to be elucidated. Moreover, our phylogenetic analysis supports the independent evolution of specific non-transformability-associated genotypes within *C. jejuni* ST-50 strains, possibly reflecting the independent evolutionary pathways in different geographic locations described by Wallace et al. [28]. This study provides a deeper understanding of the genetic mechanisms involved in the natural transformation of *Campylobacter* and highlights the complex interplay between natural transformation and bacterial evolution, genetic diversity, and AMR dissemination.

## Figures and Tables

**Table 1:** *Campylobacter* strains used for natural transformation and mutation frequencies.

C. jejuni Strains	ST	Average Transform. Frequency	TF SD	Average Mutation Frequency	MF SD	Competence of Recipient	TF/MF Log Diff.	AMR ^1^
FSIS11812945	10398	1.98 × 10^−7^	1.72 × 10^−7^	2.32 × 10^−8^	3.03 × 10^−8^	No	0–1	-
FSIS11812081	2132	1.06 × 10^−6^	4.60 × 10^−7^	3.09 × 10^−9^	2.95 × 10^−9^	Yes	≥3	-
FSIS11810577	353	6.14 × 10^−3^	5.03 × 10^−3^	1.19 × 10^−8^	1.10 × 10^−8^	Yes	≥3	-
FSIS11706266	464	3.49 × 10^−4^	3.98 × 10^−4^	3.96 × 10^−8^	1.98 × 10^−8^	Yes	≥3	-
FSIS11811270	50	1.92 × 10^−7^	4.10 × 10^−9^	1.02 × 10^−7^	1.03 × 10^−7^	No	0–1	-
FSIS11812063	50	8.68 × 10^−8^	2.93 × 10^−9^	1.16 × 10^−7^	9.51 × 10^−8^	No	0–1	-
FSIS12028218	50	1.51 × 10^−7^	8.81 × 10^−8^	2.68 × 10^−8^	2.98 × 10^−8^	No	0–1	T
FSIS12028305	50	6.76 × 10^−8^	2.79 × 10^−8^	1.77 × 10^−8^	8.26 × 10^−9^	No	0–1	T
FSIS12029464	50	1.09 × 10^−6^	1.32 × 10^−6^	1.23 × 10^−7^	2.56 × 10^−8^	No	0–1	-
FSIS12029904	50	4.42 × 10^−8^	4.81 × 10^−8^	3.54 × 10^−8^	3.89 × 10^−8^	No	0–1	-
FSIS12030287	50	1.53 × 10^−7^	1.16 × 10^−7^	7.59 × 10^−8^	6.56 × 10^−8^	No	0–1	-
FSIS12030565	50	1.03 × 10^−7^	8.59 × 10^−8^	5.66 × 10^−8^	2.71 × 10^−8^	No	0–1	-
FSIS12030692	50	3.51 × 10^−7^	3.64 × 10^−7^	4.65 × 10^−7^	4.48 × 10^−7^	No	0–1	TK
FSIS12030816	50	3.58 × 10^−8^	2.94 × 10^−8^	4.47 × 10^−8^	4.99 × 10^−8^	No	0–1	-
FSIS12031002	50	5.12 × 10^−8^	1.25 × 10^−8^	2.39 × 10^−8^	3.97 × 10^−9^	No	0–1	-
FSIS12031145	50	7.25 × 10^−8^	5.77 × 10^−8^	5.77 × 10^−7^	7.84 × 10^−7^	No	0–1	-
FSIS22028286	50	4.50 × 10^−7^	4.75 × 10^−7^	4.36 × 10^−8^	3.80 × 10^−8^	No	0–1	-
FSIS22028636	50	7.60 × 10^−8^	2.25 × 10^−8^	1.91 × 10^−7^	2.08 × 10^−7^	No	0–1	-
FSIS1607853	50	8.74 × 10^−8^	7.30 × 10^−8^	1.90 × 10^−8^	1.18 × 10^−8^	No	0–1	T
FSIS1608758	50	4.49 × 10^−8^	2.72 × 10^−8^	2.17 × 10^−7^	2.73 × 10^−7^	No	0–1	-
FSIS1609357	50	4.14 × 10^−8^	9.19 × 10^−10^	1.40 × 10^−8^	1.38 × 10^−8^	No	0–1	-
FSIS1609374	50	6.58 × 10^−8^	4.50 × 10^−8^	1.10 × 10^−8^	4.52 × 10^−9^	No	0–1	-
FSIS1709833	50	2.33 × 10^−7^	2.61 × 10^−7^	8.34 × 10^−9^	1.18 × 10^−9^	No	~2	T
FSIS1710700	50	4.10 × 10^−8^	2.52 × 10^−8^	1.26 × 10^−8^	4.30 × 10^−9^	No	0–1	-
FSIS1710996	50	2.45 × 10^−8^	1.10 × 10^−8^	2.45 × 10^−9^	1.10 × 10^−9^	No	0–1	QT
FSIS1701236	50	6.57 × 10^−7^	1.44 × 10^−7^	6.43 × 10^−8^	7.03 × 10^−8^	No	0–1	T
FSIS1702913	50	3.14 × 10^−7^	1.02 × 10^−7^	2.09 × 10^−7^	2.74 × 10^−7^	No	0–1	-
FSIS1703025	50	9.48 × 10^−8^	1.09 × 10^−7^	5.89 × 10^−8^	4.72 × 10^−8^	No	0–1	QT
FSIS11705500	50	5.22 × 10^−8^	5.23 × 10^−8^	4.35 × 10^−10^	0	No	~2	QT
FSIS21720655	50	5.26 × 10^−8^	2.77 × 10^−8^	7.66 × 10^−9^	7.69 × 10^−9^	No	0–1	QT
FSIS21720686	50	1.40 × 10^−6^	1.69 × 10^−6^	3.25 × 10^−8^	2.38 × 10^−8^	No	~2	-
FSIS21820901	50	3.20 × 10^−6^	4.32 × 10^−6^	8.85 × 10^−8^	1.11 × 10^−7^	No	~2	-
FSIS1606748	50	3.80 × 10^−8^	7.57 × 10^−9^	4.31 × 10^−8^	5.69 × 10^−8^	No	0–1	TK
FSIS1607146	50	2.30 × 10^−8^	1.77 × 10^−8^	8.87 × 10^−9^	7.54 × 10^−9^	No	0–1	T
FSIS1701497	50	2.70 × 10^−8^	3.25 × 10^−8^	2.51 × 10^−9^	2.27 × 10^−9^	No	0–1	QT
FSIS11812592	50	5.87 × 10^−7^	4.89 × 10^−7^	1.59 × 10^−7^	5.66 × 10^−9^	No	0–1	T
FSIS11917669	50	5.16 × 10^−8^	2.69 × 10^−8^	1.16 × 10^−8^	1.24 × 10^−8^	No	0–1	-
FSIS11918239	50	7.66 × 10^−8^	4.30 × 10^−8^	5.71 × 10^−8^	5.17 × 10^−8^	No	0–1	-
FSIS12028219	50	2.80 × 10^−8^	2.31 × 10^−8^	6.21 × 10^−10^	4.68 × 10^−10^	No	~2	-
FSIS12032984	50	1.20 × 10^−7^	1.52 × 10^−7^	7.50 × 10^−9^	3.13 × 10^−9^	No	~2	T
FSIS12033376	50	4.63 × 10^−8^	2.30 × 10^−8^	5.34 × 10^−8^	6.03 × 10^−8^	No	0–1	-
FSIS12138180	50	5.01 × 10^−9^	2.04 × 10^−9^	7.88 × 10^−10^	3.71 × 10^−10^	No	0–1	T
FSIS11814023	939	1.40 × 10^−7^	1.50 × 10^−7^	2.19 × 10^−8^	2.60 × 10^−8^	No	0–1	-
FSIS12028216	939	9.62 × 10^−9^	1.07 × 10^−8^	2.85 × 10^−9^	1.20 × 10^−9^	No	0–1	TK
FSIS12028439	939	4.80 × 10^−4^	3.96 × 10^−4^	1.41 × 10^−8^	1.88 × 10^−8^	Yes	≥3	T
FSIS12030679	939	2.24 × 10^−4^	1.08 × 10^−4^	3.23 × 10^−9^	3.51 × 10^−9^	Yes	≥3	-
FSIS12031025	939	3.65 × 10^−5^	2.99 × 10^−5^	6.16 × 10^−8^	8.11 × 10^−8^	Yes	≥3	K
FSIS12031178	939	3.98 × 10^−4^	2.86 × 10^−4^	4.50 × 10^−9^	1.08 × 10^−9^	Yes	≥3	T
FSIS12031661	939	2.81 × 10^−4^	3.68 × 10^−4^	3.35 × 10^−7^	4.69 × 10^−7^	Yes	≥3	-
FSIS12031779	939	1.40 × 10^−7^	1.25 × 10^−7^	4.77 × 10^−9^	3.54 × 10^−9^	No	~2	-
FSIS22027247	939	1.38 × 10^−8^	1.07 × 10^−8^	1.40 × 10^−8^	1.39 × 10^−8^	No	0–1	-
FSIS22027921	939	3.72 × 10^−8^	3.05 × 10^−8^	4.64 × 10^−9^	5.15 × 10^−9^	No	0–1	-
FSIS22028453	939	3.71 × 10^−7^	4.85 × 10^−7^	1.33 × 10^−7^	1.83 × 10^−7^	No	0–1	-
FSIS32003146	939	4.02 × 10^−4^	4.00 × 10^−4^	8.17 × 10^−10^	2.33 × 10^−11^	Yes	≥3	K
RM 3405	50	1.33 × 10^−8^	1.29 × 10^−8^	1.45 × 10^−8^	1.58 × 10^−8^	No	0–1	-
RM 3412	50	2.79 × 10^−5^	3.60 × 10^−5^	1.04 × 10^−6^	7.57 × 10^−7^	No	0–1	T
RM 5146	50	3.80 × 10^−8^	3.61 × 10^−8^	1.47 × 10^−9^	1.46 × 10^−9^	No	0–1	T
RM 5148	50	8.25 × 10^−8^	8.84 × 10^−8^	1.09 × 10^−8^	7.78 × 10^−10^	No	0–1	T
RM 5149	50	5.91 × 10^−9^	1.08 × 10^−9^	3.06 × 10^−9^	3.74 × 10^−9^	No	0–1	TQ
RM 5156	50	1.52 × 10^−4^	9.86 × 10^−5^	6.13 × 10^−8^	6.75 × 10^−8^	Yes	≥3	T
***C. coli* Strains**	**ST**	**Average Transform. Frequency**	**TF SD**	**Average Mutation Frequency**	**MF SD**	**Competence of recipient**	**TF/MF Log Diff.**	**AMR**
FSIS11813367	1050	5.76 × 10^−3^	3.69 × 10^−3^	3.48 × 10^−8^	3.75 × 10^−8^	Yes	≥3	-
FSIS1710488	7818	8.32 × 10^−4^	4.16 × 10^−5^	8.03 × 10^−9^	3.50 × 10^−9^	Yes	≥3	-
FSIS1710329	7818	1.87 × 10^−4^	2.36 × 10^−4^	1.52 × 10^−8^	1.94 × 10^−8^	Yes	≥3	-
FSIS11811291	829	8.29 × 10^−4^	4.08 × 10^−4^	1.13 × 10^−8^	1.10 × 10^−8^	Yes	≥3	E
FSIS11813365	829	4.62 × 10^−4^	2.44 × 10^−4^	2.10 × 10^−8^	1.87 × 10^−8^	Yes	≥3	E
FSIS1607221	829	4.64 × 10^−7^	2.24 × 10^−8^	3.16 × 10^−7^	4.30 × 10^−7^	No	0–1	-
FSIS21822106	829	1.25 × 10^−6^	1.49 × 10^−6^	2.31 × 10^−7^	2.42 × 10^−7^	No	0–1	-
FSIS11813852	902	6.64 × 10^−4^	3.31 × 10^−4^	1.42 × 10^−9^	1.00 × 10^−9^	Yes	≥3	Q
FSIS1710767	3262	1.89 × 10^−8^	1.05 × 10^−8^	8.17 × 10^−10^	2.33 × 10^−11^	No	~2	Q
FSIS12027778	3262	1.73 × 10^−5^	1.05 × 10^−5^	2.38 × 10^−9^	1.99 × 10^−10^	Yes	≥3	Q
FSIS12030275	3262	1.92 × 10^−8^	9.89 × 10^−9^	1.13 × 10^−9^	1.04 × 10^−9^	No	0–1	Q
FSIS12031023	3262	1.54 × 10^−3^	1.84 × 10^−3^	6.96 × 10^−9^	7.83 × 10^−9^	Yes	≥3	Q
FSIS12031175	3262	3.02 × 10^−7^	3.41 × 10^−7^	8.95 × 10^−7^	7.15 × 10^−7^	No	0–1	-
FSIS12031458	3262	1.59 × 10^−8^	1.68 × 10^−8^	2.44 × 10^−9^	6.90 × 10^−13^	No	0–1	Q
FSIS12031835	3262	1.11 × 10^−7^	8.88 × 10^−8^	1.25 × 10^−8^	3.13 × 10^−9^	No	0–1	-
FSIS12031855	3262	3.07 × 10^−8^	2.73 × 10^−8^	4.51 × 10^−9^	3.39 × 10^−9^	No	0–1	Q
FSIS22027509	3262	7.38 × 10^−4^	4.58 × 10^−4^	1.34 × 10^−8^	6.14 × 10^−9^	Yes	≥3	Q
FSIS22028223	3262	1.82 × 10^−7^	2.17 × 10^−7^	1.31 × 10^−8^	1.02 × 10^−9^	No	0–1	Q
FSIS22028629	3262	5.18 × 10^−8^	6.10 × 10^−8^	2.36 × 10^−8^	3.26 × 10^−8^	No	0–1	Q

^1^: Pan-sensitive; T: Tetracycline resistant; K: Kanamycin resistant; E: Erythromycin resistant; Q: Quinolone resistant.

**Table 2:** *Campylobacter* genetic markers affecting natural competence.

*C. jejuni* Strains	ST	*cts* Mutations ^1^	*dns* ^2^	*dns2*	*dns3*	Competence
FSIS11812945	10398	D	N	N	N	No
FSIS11812081	2132	none	N	N	Y	Yes
FSIS11810577	353	none	Y (FS)	N	N	Yes
FSIS11706266	464	none	N	N	N	Yes
FSIS11811270	50	none	Y	N	N	No
FSIS11812063	50	N.D.	N	Y	N	No
FSIS12028218	50	DE	Y	N	N	No
FSIS12028305	50	DE	Y	N	N	No
FSIS12029464	50	none	Y	N	N	No
FSIS12029904	50	none	Y	N	Y	No
FSIS12030287	50	DE	Y (FS)	N	N	No
FSIS12030565	50	DE	Y (FS)	N	N	No
FSIS12030692	50	DE	Y	N	N	No
FSIS12030816	50	none	N	Y	N	No
FSIS12031002	50	none	N	Y	Y	No
FSIS12031145	50	DE	Y (FS)	N	N	No
FSIS22028286	50	none	Y	N	N	No
FSIS22028636	50	none	N	Y	N	No
FSIS1607853	50	none	Y	N	N	No
FSIS1608758	50	DE	Y (FS)	N	N	No
FSIS1609357	50	DE	Y (FS)	N	N	No
FSIS1609374	50	none	N	Y	N	No
FSIS1709833	50	DE	N	N	N	No
FSIS1710700	50	DE	Y	N	N	No
FSIS1710996	50	none	Y	N	N	No
FSIS1701236	50	none	Y	N	N	No
FSIS1702913	50	DE	N	N	N	No
FSIS1703025	50	none	Y	N	N	No
FSIS11705500	50	D	N	N	N	No
FSIS21720655	50	D	N	N	N	No
FSIS21720686	50	none	Y	N	N	No
FSIS21820901	50	none	Y	N	N	No
FSIS1606748	50	DE	Y	N	N	No
FSIS1607146	50	none	Y	N	Y	No
FSIS1701497	50	none	Y	N	N	No
FSIS11812592	50	DE	Y	N	N	No
FSIS11917669	50	DE	N	N	N	No
FSIS11918239	50	none	Y	N	N	No
FSIS12028219	50	none	N	N	Y	No
FSIS12032984	50	N.D.	Y	N	N	No
FSIS12033376	50	none	N	Y	N	No
FSIS12138180	50	DE	Y	N	N	No
FSIS11814023	939	none	N	N	Y	No
FSIS12028216	939	none	N	N	Y	No
FSIS12028439	939	none	N	N	N	Yes
FSIS12030679	939	none	N	N	N	Yes
FSIS12031025	939	none	N	N	N	Yes
FSIS12031178	939	none	N	N	N	Yes
FSIS12031661	939	none	N	N	N	Yes
FSIS12031779	939	none	N	N	Y	No
FSIS22027247	939	none	N	N	Y	No
FSIS22027921	939	none	N	N	Y	No
FSIS22028453	939	none	N	N	Y	No
FSIS32003146	939	none	N	N	N	Yes
RM3405	50	RF	N	N	N	No
RM3412	50	none	N	N	N	No
RM5146	50	none	Y	N	N	No
RM5148	50	none	Y	N	Y	No
RM5149	50	none	Y	N	Y	No
RM5156	50	none	Y (FS)	N	N	Yes
***C. coli* Strains**	**ST**	***cts* Mutations ^1^**	***dns***	***dns2* ^3^**	***dns3***	**Competence**
FSIS11813367	1050	none	Y	N	N	Yes
FSIS1710488	7818	none	N	N	N	Yes
FSIS1710329	7818	none	N	N	N	Yes
FSIS11811291	829	none	N	N	N	Yes
FSIS11813365	829	none	N	N	N	Yes
FSIS1607221	829	none	N	N	N	No
FSIS21822106	829	none	Y	N	N	No
FSIS11813852	902	none	N	N	N	Yes
FSIS1710767	3262	none	N	Y	N	No
FSIS12027778	3262	none	N	Y (*)	N	Yes
FSIS12030275	3262	none	N	Y	N	No
FSIS12031023	3262	none	N	Y (*)	N	Yes
FSIS12031175	3262	none	N	Y	N	No
FSIS12031458	3262	none	N	Y	N	No
FSIS12031835	3262	none	N	Y	N	No
FSIS12031855	3262	none	N	Y	N	No
FSIS22027509	3262	none	N	Y (*)	N	Yes
FSIS22028223	3262	none	N	Y	N	No
FSIS22028629	3262	none	N	Y	N	No

^1^ N.D.: not determined; none: no mutation, D, E, R and F: mutations in *ctsD*, *ctsE*, *ctsR* and *ctsF*, respectively.^2^ Y: gene present; N: gene absent; Y (FS): *dns* gene contains a frameshift. ^3^ Y (*): *dns2* gene contains a nonsense mutation.

**Table 3:** *C. jejuni* ST-50 genetic markers associated with non-transformation.

Region	*cts* Mutations	Total Strains	% cts Mutations	Mu-*dns*+	Total Strains	% Mu-*dns*+	*dns2* or *dns3*	Total Strains	% *dns2* or *dns3*
Europe	37	1124	3.3	933	1450	64.3	412	1450	28.4
N. America	214	566	37.8	416	780	53.3	237	780	30.3
Australia	8	23	34.7	5	23	21.7	12	23	52.2
ST-8	49	641	7.6	62	809	7.7	218	809	26.9

## Data Availability

The DNA sequence data for this study have been deposited within NCBI BioProject as follows: RM3405 as BioProject PRJNA206196; RM3412 as BioProject PRJNA70859 and RM5146, RM5148, RM5149 and RM5156 within BioProject PRJNA982140.

## Supplementary Materials

The following supporting information can be downloaded at: https://www.mdpi.com/article/10.3390/microorganisms12020327/s1, Figure S1: Neighbor-joining dendrogram of *C. jejuni* ST-50 strains from multiple countries including the U.S. and Australia; Table S1: *Campylobacter* strains used in transformation study; Table S2: Campylobacter jejuni ST-50 strains from North America and Europe, available from PubMLST; Table S3: Campylobacter jejuni ST-50 strains from Australia; Table S4: *Campylobacter jejuni* ST-8 strains from North America, from PubMLST; Table S5: *Campylobacter jejuni* ST-50 strains from PubMLST used to create Figure 2. Neighbor-joining dendrogram of *C. jejuni* ST-50 strains.
