# Supplementary material for: Genomic Analysis Points to Multiple Genetic Mechanisms for Non-Transformable Campylobacter jejuni ST-50
Source: Microorganisms. 2024 Feb 4;12(2):327. doi: 10.3390/microorganisms12020327 (PMC10893306; doi:10.3390/microorganisms12020327)
Supplement: Supplementary file 1 [file microorganisms-12-00327-s001.zip › Figure S1-Parker_et_al2024.pdf]

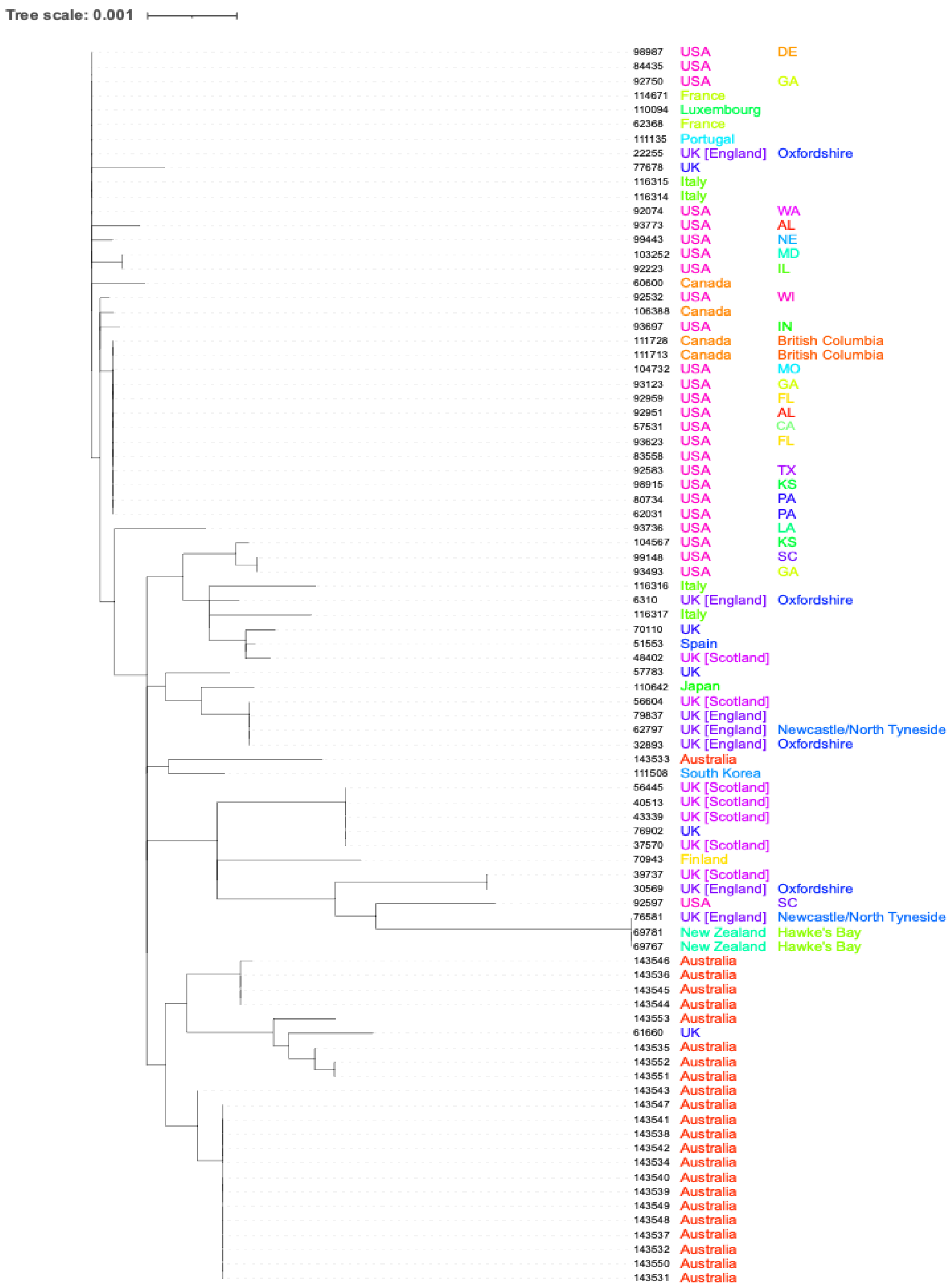

Figure S1. Neighbor-joining dendrogram of *C. jejuni* ST-50 strains from multiple countries including United States and Australia. This neighbor-joining dendrogram was created using the Interactive Tree of Life (iTOL) plugin within PubMLST using the concatenated nucleotide sequences of the *C. jejuni*/*C. coli* cgMLSTv2 dataset shared by the strains. The genomic data includes the genomic sequences from 63 *C. jejuni* ST-50 strains used for Figure 2 and 23 *C. jejuni* ST-50 strains from Australia [28]. Metadata at tree leaves show PubMLST id, country of origin, and region within country if provided.
