# Supplementary material for: Genomic Analysis Points to Multiple Genetic Mechanisms for Non-Transformable Campylobacter jejuni ST-50
Source: Microorganisms. 2024 Feb 4;12(2):327. doi: 10.3390/microorganisms12020327 (PMC10893306; doi:10.3390/microorganisms12020327)
Supplement: Supplementary file 1 [file microorganisms-12-00327-s001.zip › Table S1-Parker_et_al2024.pdf]

**Table S1.** *Campylobacter* strains used in transformation study

| *C. jejuni* strains | ST | GenBank assembly | Source | Year[1] | Country | U.S. State | AMR[2] |
|---|---|---|---|---|---|---|---|
| FSIS11812945 | 10398 | GCA_005367025.1 | Chicken | 2018 | U.S. | | - |
| FSIS11812081 | 2132 | GCA_005345325.1 | Chicken | 2018 | U.S. | | - |
| FSIS11810577 | 353 | GCA_005308685.1 | Chicken | 2018 | U.S. | | - |
| FSIS11706266 | 464 | GCA_005196475.1 | Chicken | 2017 | U.S. | | - |
| FSIS11811270 | 50 | GCA_005319705.1 | Chicken | 2018 | U.S. | SC | - |
| FSIS11812063 | 50 | GCA_005346505.1 | Chicken | 2018 | U.S. | DE | - |
| FSIS12028218 | 50 | GCA_013787785.1 | Chicken | 2020 | U.S. | PA | T |
| FSIS12028305 | 50 | GCA_010916015.1 | Chicken | 2020 | U.S. | GA | T |
| FSIS12029464 | 50 | GCA_011988645.1 | Chicken | 2020 | U.S. | GA | - |
| FSIS12029904 | 50 | GCA_012862935.1 | Chicken | 2020 | U.S. | GA | - |
| FSIS12030287 | 50 | GCA_013800385.1 | Chicken | 2020 | U.S. | GA | - |
| FSIS12030565 | 50 | GCA_013158105.1 | Chicken | 2020 | U.S. | VA | - |
| FSIS12030692 | 50 | GCA_013158405.1 | Chicken | 2020 | U.S. | TN | TK |
| FSIS12030816 | 50 | GCA_013802355.1 | Chicken | 2020 | U.S. | ME | - |
| FSIS12031002 | 50 | GCA_013742095.1 | Chicken | 2020 | U.S. | ME | - |
| FSIS12031145 | 50 | GCA_013585715.1 | Chicken | 2020 | U.S. | GA | - |
| FSIS22028286 | 50 | GCA_013718365.1 | Chicken | 2020 | U.S. | PA | - |
| FSIS22028636 | 50 | GCA_013514075.1 | Chicken | 2020 | U.S. | MD | - |
| FSIS1607853 | 50 | GCA_005199775.1 | Chicken | 2016 | U.S. | WI | T |
| FSIS1608758 | 50 | GCA_005115065.1 | Chicken | 2016 | U.S. | GA | - |
| FSIS1609357 | 50 | GCA_005127145.1 | Chicken | 2016 | U.S. | FL | - |
| FSIS1609374 | 50 | GCA_005164265.1 | Chicken | 2016 | U.S. | GA | - |
| FSIS1709833 | 50 | GCA_005127925.1 | Chicken | 2016 | U.S. | AL | T |
| FSIS1710700 | 50 | GCA_005018415.1 | Chicken | 2017 | U.S. | FL | - |
| FSIS1710996 | 50 | GCA_005268315.1 | Chicken | 2017 | U.S. | IL | QT |
| FSIS1701236 | 50 | GCA_005275875.1 | Chicken | 2017 | U.S. | WA | T |
| FSIS1702913 | 50 | GCA_005190995.1 | Chicken | 2017 | U.S. | TX | - |
| FSIS1703025 | 50 | GCA_005002075.1 | Chicken | 2017 | U.S. | IN | QT |
| FSIS11705500 | 50 | GCA_004994685.1 | Chicken | 2017 | U.S. | LA | QT |
| FSIS21720655 | 50 | GCA_004986225.1 | Chicken | 2017 | U.S. | AL | QT |
| FSIS21720686 | 50 | GCA_005056765.1 | Chicken | 2017 | U.S. | GA | - |
| FSIS21820901 | 50 | GCA_005187145.1 | Chicken | 2017 | U.S. | SC | - |
| FSIS1606748 | 50 | GCA_005293385.1 | Cattle | 2016 | U.S. | PA | TK |
| FSIS1607146 | 50 | GCA_005296665.1 | Cattle | 2016 | U.S. | NE | T |
| FSIS1701497 | 50 | GCA_005010355.1 | Cattle | 2017 | U.S. | MD | QT |
| FSIS11812592 | 50 | GCA_005350855.1 | Cattle | 2018 | U.S. | KS | T |
| FSIS11917669 | 50 | GCA_004885245.1 | Swine | 2019 | U.S. | MO | - |

| | | | | | | | |
|---|---|---|---|---|---|---|---|
| FSIS11918239 | 50 | GCA_004896895.1 | Swine | 2019 | U.S. | KS | - |
| FSIS12028219 | 50 | GCA_011722735.1 | Cattle | 2020 | U.S. | MD | - |
| FSIS12032984 | 50 | GCA_014547025.1 | Lamb | 2020 | U.S. | GA | T |
| FSIS12033376 | 50 | GCA_014660295.1 | Cattle | 2020 | U.S. | MI | - |
| FSIS12138180 | 50 | GCA_019563335.1 | Cattle | 2021 | U.S. | MI | T |
| FSIS11814023 | 939 | GCA_004810515.1 | Chicken | 2018 | U.S. | | - |
| FSIS12028216 | 939 | GCA_011723555.1 | Chicken | 2020 | U.S. | | TK |
| FSIS12028439 | 939 | GCA_010905265.1 | Chicken | 2020 | U.S. | | T |
| FSIS12030679 | 939 | GCA_013158065.1 | Chicken | 2020 | U.S. | | - |
| FSIS12031025 | 939 | GCA_013731875.1 | Chicken | 2020 | U.S. | | K |
| FSIS12031178 | 939 | GCA_013730685.1 | Chicken | 2020 | U.S. | | T |
| FSIS12031661 | 939 | GCA_013497495.1 | Chicken | 2020 | U.S. | | - |
| FSIS12031779 | 939 | GCA_013447495.1 | Chicken | 2020 | U.S. | | - |
| FSIS22027247 | 939 | GCA_011501785.1 | Chicken | 2020 | U.S. | | - |
| FSIS22027921 | 939 | GCA_012976125.1 | Chicken | 2020 | U.S. | | - |
| FSIS22028453 | 939 | GCA_013653585.1 | Chicken | 2020 | U.S. | | - |
| FSIS32003146 | 939 | GCA_010311025.1 | Chicken | 2020 | U.S. | | K |
| RM 3405 | 50 | GCA_032970625.1 | Human | 1979 | Canada | | - |
| RM 3412 | 50 | GCA_032970685.1 | Human | 1979 | Canada | | T |
| RM 5146 | 50 | GCF_032290505.1 | Human | Unk | Italy | | T |
| RM 5148 | 50 | GCF_032290485.1 | Human | Unk | Italy | | T |
| RM 5149 | 50 | GCF_032290445.1 | Human | Unk | Italy | | TQ |
| RM 5156 | 50 | GCF_032290405.1 | Chicken | Unk | Italy | | T |

| ***C. coli* strains** | **ST** | **GenBank assembly** | **Source** | **Year** | | | **AMR** |
|---|---|---|---|---|---|---|---|
| FSIS11813367 | 1050 | GCA_005374845.1 | Chicken | 2018 | U.S. | | - |
| FSIS1710488 | 7818 | GCA_005253605.1 | Chicken | 2017 | U.S. | | - |
| FSIS1710329 | 7818 | GCA_005257475.1 | Chicken | 2017 | U.S. | | - |
| FSIS11811291 | 829 | GCA_005319505.1 | Chicken | 2018 | U.S. | | E |
| FSIS11813365 | 829 | GCA_005374965.1 | Chicken | 2018 | U.S. | | E |
| FSIS1607221 | 829 | GCA_005297805.1 | Chicken | 2016 | U.S. | | - |
| FSIS21822106 | 829 | GCA_004808275.1 | Chicken | 2018 | U.S. | | - |
| FSIS11813852 | 902 | GCA_005379785.1 | Chicken | 2018 | U.S. | | Q |
| FSIS1710767 | 3262 | GCA_005258115.1 | Chicken | 2017 | U.S. | | Q |
| FSIS12027778 | 3262 | GCA_010217935.1 | Chicken | 2020 | U.S. | | Q |
| FSIS12030275 | 3262 | GCA_012975605.1 | Chicken | 2020 | U.S. | | Q |
| FSIS12031023 | 3262 | GCA_013731895.1 | Chicken | 2020 | U.S. | | Q |
| FSIS12031175 | 3262 | GCA_013730735.1 | Chicken | 2020 | U.S. | | - |
| FSIS12031458 | 3262 | GCA_013622705.1 | Chicken | 2020 | U.S. | | Q |
| FSIS12031835 | 3262 | GCA_013447195.1 | Chicken | 2020 | U.S. | | - |

| | | | | | |
|---|---|---|---|---|---|
| FSIS12031855 | 3262 | GCA_013443435.1 | Chicken | 2020 | Q |
| FSIS22027509 | 3262 | GCA_011998725.1 | Chicken | 2020 | Q |
| FSIS22028223 | 3262 | GCA_013760625.1 | Chicken | 2020 | Q |
| FSIS22028629 | 3262 | GCA_013514015.1 | Chicken | 2020 | Q |

[1]- Unk- unknown year

[2] -: pan-sensitive; T: Tetracycline resistant; K: Kanamycin resistant; E: Erythromycin resistant; Q: Quinolone resistant
