# Supplementary material for: Genomic Analysis Points to Multiple Genetic Mechanisms for Non-Transformable Campylobacter jejuni ST-50
Source: Microorganisms. 2024 Feb 4;12(2):327. doi: 10.3390/microorganisms12020327 (PMC10893306; doi:10.3390/microorganisms12020327)
Supplement: Supplementary file 1 [file microorganisms-12-00327-s001.zip › Table S2-Parker_et_al2024.pdf]

Table S2. Campylobacter jejuni ST-50 strains from North America and Europe from PubMLS

PubMLST id	Strain	Country	Year	Source	*dns* [1]	*dns2 or dns3* [2]	*cts* [3]
35308	Cj87330	USA		chicken	N	N	wt
47403	41923	USA	2009	human	Y	N	
47411	41912	USA	2008	human	Y	N	
57180	BCW_6919	USA		sheep	Y	N	
57475	BCW_4731	USA			N	Y	F
57491	BCW_5123	USA		human	Y	N	
57504	BCW_5141	USA		human	N		
57531	BCW_6872	USA		wild bird	N	N	DE
62031	FSIS1606748	USA	2016	cattle	Y	N	DE
62107	CVM N56306	USA	2015	chicken	N	Y	
62108	CVM N58758	USA	2015	chicken	N	Y	
62109	CVM N62614	USA	2015	chicken	N	Y	
62110	CVM N62615	USA	2015	chicken	N	Y	
70358	PNUSAC000034	USA	2014	human	Y	N	
70359	20100326	USA		human	Y	N	
70360	2005117	USA		human	Y	N	
70361	2014D-0170	USA		human	N	N	DE
70362	2014D-0222	USA		human	N	Y	DE
70363	2014D-0250	USA		human	N	Y	
70364	20100144	USA		human	N	N	D
70365	2012AY-1101	USA		human	Y	N	
70366	2015D-0003	USA		human	N	Y	
70367	PNUSAC000097	USA	2015	human	N	Y	
70368	PNUSAC000153	USA	2015	human	N	Y	
70369	PNUSAC000162	USA	2015	human	Y	N	DE
70370	PNUSAC000163	USA	2015	human	N	N	
70371	PNUSAC000249	USA	2015	human	N	N	DE
70373	2012D-9287	USA		human	N	Y	DE
70374	2015D-0128	USA		human	N	N	DE
70375	2015D-0096	USA		human	Y	N	DE
70376	2015D-0022	USA		human	N	Y	
70377	2012D-9292	USA		human	Y	N	
70378	PNUSAC000281	USA	2016	human	Y	N	
78540	SKS6840_20_S41	USA	2016	human stool	N	N	DE
78572	SKS6875_55_S81	USA	2016	human stool	Y	N	DE
79188	CVM N17C204	USA	2017	chicken	N	Y	
79192	CVM N17C209	USA	2017	chicken	N	Y	
79200	CVM N17C419	USA	2017	turkey	Y	N	
79210	PS00234	USA	2017	human	Y	Y	D
79211	PS00235	USA	2017	human	N	N	
79240	PS00291	USA	2017	human	N	Y	
80734	PS00309	USA	2018	human	Y	N	
80738	PS00313	USA	2018	human	N	Y	

80757	PS00332	USA	2018	human	N	Y	
80786	PS00363	USA	2018	human	N	Y	
80803	CVM N18C201	USA	2018	chicken	N	Y	
80874	PNUSAC010124	USA			N	N	
80877	PNUSAC009510	USA			Y	N	
80907	PNUSAC009920	USA			Y	N	DE
80950	PNUSAC009545	USA			Y	N	DE
80978	PNUSAC009538	USA			Y	N	DE
80985	PNUSAC009644	USA			N	N	DE
81016	PNUSAC009452	USA			Y	N	DE
81019	PNUSAC009455	USA			Y	N	DE
81031	PNUSAC009604	USA			Y	Y	DE
81052	PNUSAC009573	USA			Y	N	DE
81053	PNUSAC009572	USA			N	N	DE
81056	PNUSAC009556	USA			Y	N	
81080	PNUSAC009414	USA			N	N	
81148	PNUSAC009063	USA			Y	N	
81196	PNUSAC006088	USA			Y	N	
81205	PNUSAC005960	USA			Y	N	
81218	PNUSAC006063	USA			Y	Y	
81223	PNUSAC006067	USA			Y	N	DE
81297	PNUSAC005963	USA			N	N	DE
81316	PNUSAC005866	USA			Y	N	
81327	PNUSAC005842	USA			Y	N	
81353	PNUSAC005867	USA			N	N	
81359	PNUSAC005780	USA			Y	N	
81450	PNUSAC005826	USA			Y	Y	
81465	PNUSAC005820	USA			N	N	DE
81521	PNUSAC005594	USA			Y	N	
81566	PNUSAC005546	USA			Y	N	
81582	PNUSAC005485	USA			Y	N	
81594	PNUSAC005545	USA			Y	N	
81646	PNUSAC005622	USA			N	N	
81668	PNUSAC005627	USA			N		
81689	PNUSAC005402	USA			Y	Y	
81709	PNUSAC005578	USA			N	N	
81711	PNUSAC005520	USA			Y	N	DE
81712	PNUSAC005581	USA			Y	N	
81732	PNUSAC005528	USA			Y	N	
81737	PNUSAC005366	USA			N	N	DE
81750	PNUSAC005381	USA			Y	N	
81765	PNUSAC005484	USA			N	N	DE
81794	PNUSAC005441	USA			Y	N	
81806	PNUSAC005436	USA			N		
81857	PNUSAC005184	USA			N	N	DE
81870	PNUSAC005190	USA			N		
81880	PNUSAC005308	USA			Y	Y	

81886	PNUSAC005205	USA	Y	N	
81910	PNUSAC005309	USA	N	N	DE
81933	PNUSAC005286	USA	N	Y	
81959	PNUSAC004281	USA	Y	Y	
82015	PNUSAC005272	USA	Y	N	
82154	PNUSAC005088	USA	N	Y	
82245	PNUSAC004643	USA	Y	Y	
82280	PNUSAC000097	USA	N	Y	
82307	PNUSAC004994	USA	N	Y	
82344	PNUSAC004969	USA	Y	N	
82359	PNUSAC004906	USA	N	Y	
82364	PNUSAC004923	USA	Y	N	DE
82378	PNUSAC004834	USA	N	Y	
82384	PNUSAC004857	USA	N	N	
82393	PNUSAC004915	USA	N	N	F
82400	PNUSAC004864	USA	Y	N	DE
82437	PNUSAC004832	USA	N	N	DE
82448	PNUSAC004777	USA	Y	N	
82449	PNUSAC004766	USA	N	Y	
82456	PNUSAC004734	USA	Y	Y	
82471	PNUSAC004741	USA	Y	N	
82492	PNUSAC004661	USA	N	N	DE
82507	PNUSAC004708	USA	Y	Y	
82524	PNUSAC004701	USA	Y	N	
82525	PNUSAC003977	USA	Y	N	
82530	PNUSAC003976	USA	Y	N	
82555	PNUSAC004256	USA	N	Y	
82623	PNUSAC000987	USA	N	Y	
82641	TESTWGCAMPY0003852	USA	N	N	DE
82678	PNUSAC001933	USA	Y	N	DE
82682	PNUSAC009364	USA	Y	N	DE
82699	PNUSAC009283	USA	N	Y	
82712	PNUSAC009249	USA	Y	N	
82734	PNUSAC002012	USA	N	N	DE
82776	PNUSAC008916	USA	N		
82793	PNUSAC002010	USA	N	N	DE
82827	PNUSAC001968	USA	N	Y	
82830	PNUSAC001969	USA	N	Y	
82875	PNUSAC002165	USA	N	Y	
82902	PNUSAC001645	USA	N	Y	
82980	PNUSAC000516	USA	Y	N	
83002	PNUSAC000885	USA	N	N	
83025	PNUSAC000707	USA	Y	N	
83044	TESTWGCAMPY0004063	USA	Y	Y	
83076	PNUSAC002121	USA	N	N	
83134	PNUSAC002811	USA	Y	N	DE
83201	PNUSAC008944	USA	N	Y	

83210	PNUSAC008541	USA	N	N	
83340	PNUSAC000952	USA	N	N	DE
83360	PNUSAC000874	USA	N	Y	
83439	PNUSAC004461	USA	N	N	
83463	PNUSAC004166	USA	Y	N	
83487	PNUSAC002178	USA	N	N	
83532	PNUSAC002523	USA	N	N	
83558	2016D-0258	USA	Y	N	DE
83607	PNUSAC002948	USA	Y	N	DE
83683	PNUSAC003416	USA	N	N	DE
83685	PNUSAC002653	USA	Y	N	
83691	PNUSAC003826	USA	N	Y	
83694	PNUSAC002895	USA	N	N	DE
83708	PNUSAC003141	USA	Y	N	
83720	PNUSAC002886	USA	Y	N	
83723	PNUSAC003340	USA	Y	N	
83737	PNUSAC003431	USA	N	Y	
83831	PNUSAC001657	USA	N	Y	
83836	PNUSAC001722	USA	N	N	
83856	PNUSAC001635	USA	N	N	
83902	PNUSAC003000	USA	N	Y	
83953	PNUSAC004279	USA	Y	Y	
83975	PNUSAC003735	USA	N	Y	
83989	PNUSAC003729	USA	N		
84005	PNUSAC001694	USA	N		DE
84024	PNUSAC000722	USA	Y	N	DE
84034	PNUSAC001133	USA	N	Y	
84039	PNUSAC001466	USA	Y	N	DE
84052	PNUSAC003075	USA	N	Y	
84091	PNUSAC001613	USA	Y	N	
84117	PNUSAC000471	USA	N	Y	
84156	PNUSAC001465	USA	Y	N	DE
84206	PNUSAC000750	USA	N	N	
84248	PNUSAC001136	USA	Y	N	DE
84250	PNUSAC002740	USA	N	N	
84336	PNUSAC001841	USA	Y	N	
84338	PNUSAC001665	USA	Y	N	
84364	PNUSAC000515	USA	Y	N	
84380	PNUSAC000436	USA	N	Y	
84435	2016D-0064	USA	N	Y	
84501	TESTWGCAMPY0003766	USA	N	Y	DE
84522	PNUSAC000258	USA	Y	N	
84537	PNUSAC000264	USA	Y	N	
84557	PNUSAC000116	USA	Y	N	
84563	PNUSAC000130	USA	Y	N	DE
84574	TESTWGCAMPY10002671	USA	N	Y	
84653	TESTWGCAMPY0002095	USA	N	Y	DE

84697	TESTWGCAMPY0002353	USA	Y	N	
84726	PNUSAC009154	USA	Y	N	DE
84758	PNUSAC001232	USA	Y	N	
84775	PNUSAC001413	USA	Y	N	
84782	PNUSAC001287	USA	N	Y	
84814	PNUSAC008544	USA	Y	N	
84829	PNUSAC001468	USA	Y	N	DE
84901	PNUSAC001254	USA	Y	N	
84918	PNUSAC001189	USA	N	N	DE
84934	PNUSAC001179	USA	N	Y	
84941	PNUSAC001053	USA	N	Y	
84947	PNUSAC001098	USA	Y	N	
84973	PNUSAC000994	USA	Y	N	DE
85120	PNUSAC000592	USA	N	N	DE
85148	TESTWGCAMPY0003975	USA	N	N	DE
85153	TESTWGCAMPY0004054	USA	Y	N	DE
85189	PNUSAC000494	USA	Y	N	DE
85202	TESTWGCAMPY0004103	USA	N	Y	
85214	PNUSAC000518	USA	Y	N	
85291	PNUSAC000115	USA	N	Y	
85333	TESTWGCAMPY10002672	USA	Y	N	
85366	TESTWGCAMPY0002061	USA	N	N	DE
85393	PNUSAC000007	USA	N	Y	
85432	PNUSAC000032	USA	N	Y	
85436	PNUSAC007736	USA	N	N	DE
85440	PNUSAC007737	USA	N	N	DE
85453	PNUSAC009003	USA	Y	Y	
85454	PNUSAC007739	USA	Y	N	DE
85481	PNUSAC008948	USA	Y	N	
85581	PNUSAC004473	USA	Y	Y	
85653	PNUSAC004207	USA	Y	Y	
85656	PNUSAC004144	USA	N	N	DE
85660	PNUSAC004089	USA	N	N	
85785	PNUSAC003676	USA	unk		
85799	PNUSAC004332	USA	N	Y	
85824	PNUSAC004079	USA	N	N	DE
85839	PNUSAC004208	USA	Y	Y	
85840	PNUSAC004325	USA	Y	N	
85904	PNUSAC004226	USA	N	Y	
85927	PNUSAC004221	USA	N	Y	
85947	PNUSAC003821	USA	N	N	
85957	PNUSAC003738	USA	N	Y	
85974	PNUSAC003736	USA	N	Y	
85999	PNUSAC003568	USA	Y	N	DE
86029	PNUSAC004399	USA	N	N	DE
86033	PNUSAC004114	USA	N	Y	
86243	PNUSAC002913	USA	N		

86273	PNUSAC003778	USA	N	Y	
86291	PNUSAC003665	USA	N	N	DE
86322	PNUSAC003339	USA	Y	N	
86363	PNUSAC003125	USA	N	Y	
86368	PNUSAC003475	USA	N	N	DE
86399	PNUSAC002915	USA	Y	N	DE
86421	PNUSAC003290	USA	Y	N	
86442	PNUSAC003239	USA	Y	N	DE
86452	PNUSAC002284	USA	N	Y	
86519	PNUSAC002474	USA	N	N	E
86561	PNUSAC002835	USA	N	N	DE
86654	PNUSAC002777	USA	N	N	
86688	PNUSAC002385	USA	Y	N	
86700	PNUSAC001957	USA	N	N	DE
86701	PNUSAC002882	USA	Y	N	DE
86781	PNUSAC002147	USA	Y	N	DE
86783	PNUSAC002598	USA	Y	N	
86790	PNUSAC002363	USA	N	Y	
86801	PNUSAC002327	USA	N	Y	
86809	PNUSAC002758	USA	N	N	
86810	PNUSAC001298	USA	N	Y	
86873	PNUSAC003695	USA	Y	Y	
86902	PNUSAC003540	USA	N	N	
86969	PNUSAC003433	USA	N	Y	
86976	PNUSAC003451	USA	N	N	E
86989	PNUSAC003355	USA	Y	Y	
86991	PNUSAC003353	USA	N	Y	
86994	PNUSAC003341	USA	N	N	
87001	PNUSAC003417	USA	N	N	DE
87044	PNUSAC003185	USA	N	Y	
87061	PNUSAC003250	USA	Y	N	
87063	PNUSAC003178	USA	Y	N	DE
87073	PNUSAC003064	USA	N	N	E
87079	PNUSAC000589	USA	N	Y	
87102	PNUSAC000583	USA	Y	N	
87118	PNUSAC003176	USA	N	N	DE
87134	PNUSAC000367	USA	Y	N	
87144	PNUSAC003143	USA	N	Y	
87215	PNUSAC002989	USA	Y	N	
87217	PNUSAC002926	USA	Y	N	DE
87231	PNUSAC003096	USA	Y	N	
87266	PNUSAC002837	USA	N	N	
87299	PNUSAC002670	USA	N	N	DE
87333	PNUSAC002665	USA	Y	N	
87413	PNUSAC002475	USA	N	N	E
87420	PNUSAC002434	USA	N	Y	
87428	PNUSAC002552	USA	N	Y	

87440	PNUSAC002471	USA	N	Y	
87456	PNUSAC002321	USA	Y	N	
87468	PNUSAC002329	USA	Y	N	DE
87496	PNUSAC002282	USA	N		
87571	PNUSAC002054	USA	Y	N	
87581	PNUSAC002047	USA	N	Y	
87665	PNUSAC001550	USA	Y	Y	
87735	PNUSAC001147	USA	Y	N	
87759	PNUSAC000971	USA	N	N	
87763	PNUSAC000970	USA	N	Y	
87776	PNUSAC000868	USA	N	Y	
87781	PNUSAC000871	USA	Y	N	
87854	PNUSAC000464	USA	Y	Y	
87960	TESTWGCAMPY0004060	USA	N	Y	
88024	PNUSAC000128	USA	N	Y	
88037	TESTWGCAMPY0002438	USA	Y	N	
88051	TESTWGCAMPY0002350	USA	N	N	D
88065	TESTWGCAMPY0002110	USA	N	Y	
88117	TESTWGCAMPY0002405	USA	Y	N	
88135	PNUSAC000063	USA	N	Y	
88150	PNUSAC000005	USA	N	Y	
88226	PNUSAC008805	USA	Y	N	F
88246	PNUSAC008535	USA	N	N	DE
88253	PNUSAC008811	USA	Y	N	
88267	PNUSAC008714	USA	Y	N	DE
88275	PNUSAC008521	USA	Y	N	
88287	PNUSAC008599	USA	Y	N	DE
88340	PNUSAC008591	USA	N	Y	
88365	PNUSAC008580	USA	N	N	
88446	PNUSAC007702	USA	N	N	DE
88452	PNUSAC007679	USA	N	N	
88455	PNUSAC007668	USA	Y	N	DE
88460	PNUSAC007648	USA	N	N	DE
88483	PNUSAC007572	USA	N	Y	
88484	PNUSAC007544	USA	N	N	F
88499	PNUSAC007266	USA	N	Y	
88500	PNUSAC007559	USA	Y	N	
88550	PNUSAC007327	USA	Y	N	
88552	PNUSAC007203	USA	N	Y	
88556	PNUSAC007282	USA	N	N	DE
88626	PNUSAC008468	USA	N	N	DE
88653	PNUSAC006867	USA	Y	N	DE
88742	PNUSAC007104	USA	Y	N	DE
88743	PNUSAC007003	USA	N	N	
88753	PNUSAC007023	USA	Y	N	
88759	PNUSAC006901	USA	N	Y	
88773	PNUSAC006942	USA	N	N	

88812	PNUSAC007790	USA	N	N	DE
88872	PNUSAC006892	USA	N	N	DE
88875	PNUSAC006828	USA	Y	N	DE
88877	PNUSAC006804	USA	Y	N	DE
88878	PNUSAC006825	USA	N	N	
88885	PNUSAC006803	USA	N	N	DE
88886	PNUSAC006688	USA	Y	N	
88927	PNUSAC007376	USA	N		
88939	PNUSAC004301	USA	N	Y	
88942	PNUSAC008412	USA	N	N	
88954	PNUSAC008039	USA	N		
88958	PNUSAC008140	USA	N	N	DE
88973	PNUSAC008378	USA	N	N	
89038	PNUSAC008041	USA	N	Y	
89061	PNUSAC008134	USA	N	N	
89078	PNUSAC007851	USA	Y	Y	
89129	PNUSAC008135	USA	N	N	
89138	PNUSAC007944	USA	Y	N	
89186	PNUSAC007799	USA	N	N	DE
89204	PNUSAC008126	USA	Y	N	DE
89210	PNUSAC007485	USA	Y	N	
89237	PNUSAC007798	USA	Y	N	
89242	PNUSAC007809	USA	Y	N	
89243	PNUSAC007709	USA	N	N	
89244	PNUSAC008087	USA	N	N	DE
89313	PNUSAC007961	USA	N	Y	
89318	PNUSAC007985	USA	N	N	DE
89322	PNUSAC007999	USA	N	Y	
89335	PNUSAC007466	USA	Y	N	
89361	PNUSAC007498	USA	N		
89378	PNUSAC007462	USA	Y	N	
89399	PNUSAC007934	USA	Y	N	DE
89559	PNUSAC007262	USA	Y	N	
89591	PNUSAC007324	USA	Y	N	
89611	PNUSAC007530	USA	Y	N	
89634	PNUSAC007263	USA	Y	N	
89699	PNUSAC006903	USA	Y	N	DE
89701	PNUSAC006871	USA	Y	N	
89745	PNUSAC007130	USA	Y	N	
89777	PNUSAC007122	USA	Y	N	
89842	PNUSAC006978	USA	N	Y	
89847	PNUSAC006972	USA	N	Y	
89857	PNUSAC006977	USA	N	Y	
89866	PNUSAC006778	USA	Y	N	
89871	PNUSAC006776	USA	Y	N	E
89905	PNUSAC006819	USA	Y	N	
89961	PNUSAC006542	USA	Y	N	

89963	PNUSAC006545	USA	Y	N	
90025	PNUSAC006425	USA	N	N	
90035	PNUSAC006411	USA	Y	N	
90037	PNUSAC006419	USA	Y	N	DE
90052	PNUSAC006363	USA	Y	N	DE
90053	PNUSAC006388	USA	Y	N	DE
90057	PNUSAC006390	USA	Y	N	
90099	PNUSAC006445	USA	Y	Y	
90100	PNUSAC006349	USA	N	N	DE
90106	PNUSAC006415	USA	Y	N	DE
90171	PNUSAC006210	USA	Y	N	DE
90178	PNUSAC006182	USA	Y	N	
90186	PNUSAC006165	USA	Y	N	
90205	PNUSAC005892	USA	Y	N	
90228	PNUSAC006101	USA	N	N	DE
90237	PNUSAC006112	USA	Y	N	
90274	PNUSAC006145	USA	N	N	
90293	PNUSAC006228	USA	N	N	DE
90307	PNUSAC006225	USA	N	N	
90315	PNUSAC006238	USA	N	N	DE
90322	PNUSAC006384	USA	Y	N	
90323	PNUSAC006529	USA	N	N	DE
90326	PNUSAC006269	USA	N	N	DE
90329	PNUSAC006537	USA	N	N	DE
90384	PNUSAC006365	USA	Y	N	
90387	PNUSAC006389	USA	Y	N	
90389	PNUSAC005958	USA	N		
90413	PNUSAC006750	USA	N	Y	
90427	PNUSAC006498	USA	Y	N	
90445	PNUSAC006496	USA	Y	N	
90469	PNUSAC006730	USA	N	Y	DE
90476	PNUSAC006755	USA	Y	N	
90494	PNUSAC006709	USA	N	Y	
90524	PNUSAC006666	USA	Y	N	DE
90571	PNUSAC006261	USA	Y	N	
90572	PNUSAC006591	USA	Y	N	DE
90585	PNUSAC006597	USA	Y	N	
90602	FSIS21924683	USA	N	Y	D
90609	FSIS11922163	USA	Y	Y	
90620	FSIS31902106	USA	Y	N	DE
90648	FSIS11921568	USA	N	Y	
90655	FSIS11921791	USA	Y	N	DE
90659	FSIS11921799	USA	Y	N	DE
90710	FSIS31902120	USA	N	Y	
90778	FSIS11921537	USA	Y	N	DE
90786	FSIS31902084	USA	Y	N	
90796	FSIS21924463	USA	Y	N	

90882	FSIS21924418	USA	N	Y	
90891	FSIS11921297	USA	Y	N	
90984	FSIS31901943	USA	Y	N	
91055	FSIS21924230	USA	Y	N	
91082	FSIS21924191	USA	Y	N	
91088	FSIS21924180	USA	Y	N	DE
91100	FSIS11813653	USA	Y	Y	
91114	FSIS11813635	USA	N	Y	
91143	FSIS11813643	USA	N	Y	
91152	FSIS11813628	USA	Y	Y	DE
91180	FSIS21822001	USA	Y	N	
91193	FSIS21822000	USA	Y	N	DE
91207	FSIS11813342	USA	Y	N	DE
91236	FSIS21821908	USA	N	N	DE
91241	FSIS21821905	USA	Y	N	
91244	FSIS21821906	USA	Y	N	
91273	FSIS11813138	USA	Y	Y	
91281	FSIS11813120	USA	N	Y	
91288	FSIS11813143	USA	N	Y	
91311	FSIS11813108	USA	N		
91389	FSIS11812667	USA	Y	N	
91416	FSIS11812654	USA	N	N	
91430	FSIS31800817	USA	N	N	DE
91446	FSIS11812354	USA	Y	N	
91449	FSIS11812357	USA	N	Y	
91457	FSIS11812355	USA	N	Y	
91461	FSIS11812346	USA	N	Y	
91484	FSIS11812371	USA	N	Y	
91487	FSIS11812150	USA	N		
91493	FSIS11812375	USA	N		
91499	FSIS11812152	USA	Y	N	
91511	FSIS11812129	USA	Y	N	
91514	FSIS11812126	USA	Y	N	
91519	FSIS11812137	USA	Y	N	
91529	FSIS11812121	USA	Y	N	
91535	FSIS11812115	USA	Y	N	DE
91560	FSIS11811839	USA	Y	Y	DE
91563	FSIS11811800	USA	Y	Y	DE
91580	FSIS11811828	USA	N	N	DE
91671	FSIS11811321	USA	N	N	DE
91673	FSIS11811353	USA	N	Y	
91728	FSIS11811224	USA	N	N	
91749	FSIS11810827	USA	N		
91757	FSIS11811038	USA	N	Y	
91788	FSIS31800608	USA	Y	N	
91860	FSIS11810806	USA	N	Y	
91872	FSIS11810182	USA	N	Y	

91884	FSIS11810168	USA	Y	N	
91908	FSIS31800560	USA	Y	N	DE
91932	FSIS31800545	USA	Y	N	
91938	FSIS21821758	USA	Y	N	
92065	FSIS21924168	USA	Y	N	DE
92074	FSIS1701236	USA	Y	N	
92086	FSIS1700919	USA	Y	N	
92088	FSIS1700917	USA	Y	N	
92157	FSIS11920295	USA	Y	N	DE
92223	FSIS1710996	USA	Y	N	
92230	FSIS1710834	USA	N	N	
92231	FSIS1700373	USA	Y	N	DE
92233	FSIS1700206	USA	N	Y	
92242	FSIS1710999	USA	Y	N	
92290	FSIS11920076	USA	Y	N	DE
92317	FSIS21924022	USA	N	Y	
92319	FSIS21924141	USA	N	Y	
92339	FSIS21923952	USA	N	Y	
92340	FSIS21923964	USA	Y	N	
92341	FSIS21924083	USA	N	N	DE
92366	FSIS21924091	USA	N	Y	E
92369	FSIS31901835	USA	N	Y	
92379	FSIS11918860	USA	N	N	
92391	FSIS11920069	USA	Y	Y	
92420	FSIS11919363	USA	N	Y	
92436	FSIS1710695	USA	N	Y	DE
92455	FSIS1710706	USA	Y	N	DE
92472	FSIS1609690	USA	N	N	
92474	FSIS1703347	USA	Y	N	
92532	FSIS1607853	USA	Y	N	
92573	FSIS1703024	USA	N	Y	X
92583	FSIS1702913	USA	N	N	DE
92597	FSIS21820901	USA	Y	Y	
92625	FSIS11705196	USA	Y	N	DE
92632	FSIS11808250	USA	Y	N	
92640	FSIS1608924	USA	Y	N	R
92653	FSIS1609523	USA	N	Y	
92706	FSIS11704848	USA	Y	Y	
92718	FSIS31800460	USA	N	N	DE
92739	FSIS1609736	USA	Y	N	
92750	FSIS1609374	USA	N	Y	
92761	FSIS1609205	USA	Y	N	DE
92795	FSIS1608913	USA	N	Y	
92843	FSIS21720586	USA	Y	N	
92849	FSIS1710531	USA	Y	N	DE
92858	FSIS1710333	USA	Y	N	
92939	FSIS11918846	USA	Y	N	DE

92951	FSIS1709833	USA	N	N	DE
92953	FSIS1709945	USA	N	N	
92959	FSIS1609357	USA	Y	N	DE
92973	FSIS31901885	USA	Y	N	DE
93048	FSIS11919629	USA	Y	N	
93110	FSIS1609739	USA	N	Y	
93123	FSIS1608758	USA	Y	N	
93158	FSIS1608343	USA	Y	N	
93159	FSIS1608209	USA	N	N	DE
93235	FSIS11918662	USA	Y	N	
93287	FSIS11919395	USA	N	Y	
93301	FSIS11919145	USA	N	N	DE
93317	FSIS11918895	USA	Y	N	
93328	FSIS11919633	USA	Y	N	DE
93333	FSIS11919597	USA	N	N	DE
93363	FSIS31800314	USA	Y	N	
93380	FSIS31800225	USA	Y	Y	
93401	FSIS31800462	USA	N	Y	
93405	FSIS1710995	USA	Y	N	
93442	FSIS31800410	USA	N	N	DE
93468	FSIS21821541	USA	N	Y	
93493	FSIS21720686	USA	Y	N	
93520	FSIS31800272	USA	N	Y	
93530	FSIS11807971	USA	Y	N	
93536	FSIS11807307	USA	Y	N	
93548	FSIS11706119	USA	Y	N	
93553	FSIS11704847	USA	N	N	
93559	FSIS21720654	USA	N	N	
93564	FSIS21720232	USA	N	Y	
93587	FSIS11704003	USA	Y	N	DE
93594	FSIS1700744	USA	Y	N	DE
93623	FSIS1710700	USA	Y	N	DE
93630	FSIS1703177	USA	Y	N	
93697	FSIS1703025	USA	Y	N	
93708	FSIS31800185	USA	Y	N	DE
93736	FSIS11705500	USA	N		
93753	FSIS11704929	USA	Y	Y	DE
93757	FSIS21720818	USA	Y	N	
93765	FSIS21720820	USA	N	Y	
93773	FSIS21720655	USA	N	N	D
93789	FSIS21720461	USA	Y	N	
93804	FSIS21720316	USA	Y	N	DE
93884	FSIS1701995	USA	Y	N	E
93961	FSIS1608029	USA	Y	N	
94037	FSIS1501560	USA	Y	Y	
94053	FSIS21923794	USA	N	N	DE
94072	FSIS21923799	USA	Y	N	

94111	FSIS11918412	USA	Y	N	
94113	FSIS11918420	USA	N		
94137	FSIS21923734	USA	N	N	DE
94238	FSIS11815845	USA	N	Y	
94251	FSIS11815630	USA	Y	N	
94252	FSIS11918270	USA	Y	N	
94256	FSIS11918272	USA	Y	N	
94273	FSIS21923187	USA	N	Y	
94309	FSIS11816242	USA	Y	N	DE
94336	FSIS31801230	USA	N	Y	
94338	FSIS21822789	USA	Y	N	
94386	FSIS31901461	USA	N	N	D
94406	FSIS11815436	USA	N	Y	
94413	FSIS31801198	USA	Y	Y	
94436	FSIS21923633	USA	N	N	DE
94444	FSIS21923643	USA	Y	N	DE
94482	FSIS11917902	USA	Y	N	
94491	FSIS21923604	USA	N	N	DE
94532	FSIS11917702	USA	N	N	DE
94549	FSIS11917455	USA	Y	N	
94586	FSIS11917467	USA	N	Y	
94591	FSIS11917470	USA	Y	Y	
94602	FSIS11917306	USA	Y	N	
94625	FSIS11917272	USA	N	N	
94681	FSIS21923462	USA	N	N	
94692	FSIS21923515	USA	Y	Y	DE
94699	FSIS21923470	USA	N	Y	
94707	FSIS21923457	USA	Y	N	
94748	FSIS11917023	USA	Y	Y	DE
94749	FSIS11816822	USA	Y	N	DE
94759	FSIS21923406	USA	N		
94763	FSIS21923377	USA	Y	N	
94811	FSIS11816841	USA	Y	N	
94817	FSIS31901495	USA	Y	N	
94826	FSIS11917039	USA	Y	N	
94874	FSIS11816584	USA	Y	N	DE
94879	FSIS11816589	USA	N	Y	
94901	FSIS11816610	USA	Y	N	
94903	FSIS21923358	USA	N	Y	
94907	FSIS21923279	USA	Y	N	
94948	FSIS21923254	USA	Y	Y	
94954	FSIS21923198	USA	N		
94975	FSIS21923178	USA	N	Y	
95121	FSIS11816239	USA	Y	N	
95128	FSIS11816264	USA	N	Y	
95188	FSIS21822926	USA	N	Y	
95194	FSIS11815847	USA	Y	N	

95251	FSIS21822899	USA	Y	N	
95338	FSIS21822792	USA	Y	N	
95345	FSIS21822799	USA	N	N	DE
95352	FSIS21822586	USA	Y	N	DE
95354	FSIS21822600	USA	N	Y	
95374	FSIS21822571	USA	Y	Y	
95396	FSIS11815399	USA	N	N	D
95430	FSIS21822407	USA	Y	Y	
95462	FSIS21822385	USA	N	Y	
95473	FSIS21822371	USA	Y	Y	
95499	FSIS11814564	USA	Y	N	DE
95535	FSIS11814390	USA	Y	N	
95570	FSIS21822127	USA	N	N	
95575	FSIS21822209	USA	N	N	DE
95617	FSIS11814186	USA	Y	N	
95816	FSIS11814971	USA	N	N	D
95833	FSIS11814800	USA	Y	N	DE
95867	FSIS11815200	USA	Y	N	
95874	FSIS11814992	USA	Y	N	DE
95876	FSIS31801175	USA	N	N	DE
95887	FSIS21822453	USA	N	Y	
95890	FSIS21822450	USA	Y	N	DE
96048	CVM N17C829	USA	Y	N	
96049	CVM N17C823	USA	N	N	D
96062	CVM N17C570	USA	N	Y	
96063	CVM N17C578	USA	Y	N	DE
96081	CVM N17C560	USA	Y	N	
96092	CVM N17C753	USA	Y	N	
96096	CVM N17C751	USA	Y	N	
96119	CVM N17C460	USA	Y	N	
96126	CVM N17C476	USA	Y	N	DE
96131	CVM N17C795	USA	Y	Y	
96149	CVM N17C731	USA	N	N	DE
96156	CVM N17C459	USA	Y	N	
96163	CVM N17C793	USA	Y	Y	
96166	CVM N17C830	USA	Y	N	
96172	CVM N17C779	USA	Y	N	
96187	CVM N17C722	USA	N	Y	
96204	CVM N17C692	USA	Y	N	
96284	CVM N17C755	USA	Y	N	
96291	CVM N17C359	USA	N	Y	
96292	CVM N17C754	USA	Y	N	
96298	CVM N17C257	USA	Y	N	
96304	CVM N17C281	USA	N	N	
96317	CVM N17C756	USA	Y	N	
96345	CVM N17C752	USA	Y	N	
96361	CVM N17C654	USA	N	N	

96381	CVM N17C631	USA	Y	N	
96452	CVM N17C589	USA	N	N	
96532	CVM N17C641	USA	Y	N	
96590	CVM N17C178	USA	Y	N	
96599	CVM N17C181	USA	N	Y	
96658	CVM N17C643	USA	Y	Y	
96675	CVM N17C265	USA	N	Y	
96743	CVM N17C564	USA	Y	Y	DE
96792	CVM N17C565	USA	Y	Y	DE
96836	CVM N59387	USA	Y	Y	
96862	CVM N59351	USA	N	N	DE
96866	CVM N59348	USA	N	N	DE
96892	CVM N58779	USA	Y	Y	
96908	CVM N59404	USA	N	N	DE
96921	CVM N59365	USA	Y	N	
96958	CVM N58709	USA	Y	Y	
96976	CVM N58128	USA	N	Y	
97034	CVM N56285	USA	N	Y	
97035	CVM N56265	USA	N	Y	
97047	CVM N56270	USA	Y	N	
97048	CVM N55764	USA	Y	N	F
97104	CVM N16C099	USA	N	Y	
97127	CVM N16C548	USA	Y	N	DE
97128	CVM N16C568	USA	Y	N	
97143	CVM N62984	USA	Y	Y	
97144	CVM N16C178	USA	N	Y	
97149	CVM N62988	USA	Y	Y	
97161	CVM N62658	USA	N		
97172	CVM N16C496	USA	Y	N	DE
97221	CVM N62586	USA	Y	Y	DE
97228	CVM N62611	USA	Y	Y	
97259	CVM N16C435	USA	Y	Y	
97273	CVM N16C237	USA	Y	N	
97293	CVM N16C369	USA	Y	N	
97310	CVM N16C368	USA	N	N	
97338	CVM N58102	USA	Y	Y	
97410	CVM N16C343	USA	Y	N	
97503	CVM N56604	USA	Y	N	
97526	CVM N62980	USA	Y	Y	
97552	CVM N16C027	USA	N	Y	
97554	CVM N16C042	USA	N	N	DE
97570	CVM N16C117	USA	N	Y	
97595	CVM N62641	USA	Y	N	
97615	CVM N55920	USA	N	Y	
97635	CVM N16C507	USA	N	N	DE
97646	CVM N16C485	USA	N	N	
97666	CVM N16C389	USA	N	N	

97673	CVM N16C375	USA	Y	N	DE
97685	CVM N16C558	USA	Y	N	
97715	CVM N16C365	USA	Y	N	
97721	CVM N16C364	USA	Y	N	
97798	CVM N16C060	USA	Y	N	
97838	CVM N16C028	USA	N	Y	
97867	CVM N62987	USA	Y	Y	
97884	CVM N62589	USA	N	Y	
97894	CVM N62578	USA	N	N	
97943	CVM N55763	USA	Y	N	F
97967	CVM N18C006	USA	N	N	
97975	CVM N18C015	USA	Y	N	DE
97977	CVM N18C014	USA	Y	N	DE
97995	CVM N18C080	USA	N	Y	
97999	CVM N18C077	USA	Y	N	
98015	CVM N18C081	USA	Y	N	DE
98040	CVM N18C065	USA	Y	N	
98051	CVM N18C064	USA	Y	N	
98060	CVM N18C049	USA	N		
98089	CVM N18C051	USA	N		
98334	CVM N18C223	USA	Y	N	
98343	FSIS11922137	USA	N	Y	
98452	FSIS11921474	USA	N	Y	
98801	FSIS11813162	USA	Y	N	
98905	FSIS11812590	USA	Y	N	DE
98915	FSIS11812592	USA	Y	N	DE
98975	FSIS11812589	USA	Y	N	
98987	FSIS11812063	USA	N	Y	
98989	FSIS11812061	USA	N		
99040	FSIS11811883	USA	Y	N	
99148	FSIS11811270	USA	Y	N	
99214	FSIS11810554	USA	N	Y	
99248	FSIS11810409	USA	N	Y	
99443	FSIS1607146	USA	Y	Y	
100444	FSIS11919764	USA	N	N	D
100647	FSIS11918820	USA	Y	N	
100768	FSIS11809509	USA	N	Y	
101135	FSIS11808045	USA	N	Y	
101239	FSIS11807497	USA	Y	N	DE
101660	FSIS11919107	USA	Y	Y	
101788	FSIS11919302	USA	Y	N	DE
102072	FSIS1504654	USA	Y	Y	
102112	FSIS11919568	USA	Y	N	
102263	FSIS11808595	USA	Y	Y	
102381	FSIS11808742	USA	N	Y	
102657	FSIS11809682	USA	N	N	DE
103002	FSIS11706720	USA	N	N	DE

103252	FSIS1701497	USA			Y	N	
103320	FSIS1703269	USA			Y	N	
103551	FSIS11706472	USA			Y	Y	DE
104379	FSIS11918488	USA			Y	N	
104456	FSIS11918425	USA			Y	Y	
104567	FSIS11918239	USA			Y	N	
104675	FSIS11917691	USA			N	N	DE
104678	FSIS11917690	USA			N	N	DE
104732	FSIS11917669	USA			N	N	DE
104816	FSIS11816890	USA			Y	N	
105028	FSIS11816680	USA			N	N	
105109	FSIS11815680	USA			Y	N	
105169	FSIS11814751	USA			Y	Y	DE
105200	FSIS11814428	USA			N	N	DE
106329	SFBRC-21	USA	2013	waters	Y	N	DE
106388	RM3405	Canada	1980		N	N	RF
106393	RM3412	Canada	1979		N	N	
108905	KKC292	USA	2018	human stool	N	Y	
111590	CVM N17C578	USA	2017	chicken	Y	N	DE
111713	R1S3-12	Canada	2020	broiler environ	Y	N	
111714	R1S3-16	Canada	2020	broiler environ	Y	N	
111715	R1S3-17A	Canada	2020	broiler environ	Y	N	
111716	R1S3-17B	Canada	2020	broiler environ	Y	N	
111717	R1S3-18A	Canada	2020	broiler environ	Y	N	
111718	R1S3-18B	Canada	2020	broiler environ	Y	N	
111719	R1S3-22	Canada	2020	broiler environ	Y	N	
111720	R1S3-23B	Canada	2020	broiler environ	Y	N	
111728	R1S3-W2A	Canada	2020	chicken	**Y**	**N**	
2605	H142940464	UK [England]	2014	other animal	Y	N	
2670	H140620012	UK [England]	2014	human stool	Y	N	
2671	H140940807	UK [England]	2014	human stool	Y	N	
5942	OXC85	UK [England]	2003	human stool	N	Y	
5949	OXC92	UK [England]	2003	human stool	Y	Y	
5955	OXC98	UK [England]	2003	human stool	Y	N	
5963	OXC106	UK [England]	2003	human stool	Y	N	
6001	OXC144	UK [England]	2003	human stool	Y	N	
6016	OXC159	UK [England]	2003	human stool	Y	N	
6022	OXC165	UK [England]	2003	human stool	Y	N	
6056	OXC199	UK [England]	2004	human stool	Y	N	
6103	OXC246	UK [England]	2004	human stool	Y	N	
6158	OXC301	UK [England]	2004	human stool	Y	N	
6189	OXC332	UK [England]	2004	human stool	Y	N	
6192	OXC335	UK [England]	2004	human stool	Y	Y	
6214	OXC357	UK [England]	2004	human stool	N	Y	
6236	OXC379	UK [England]	2004	human stool	Y	Y	
6237	OXC380	UK [England]	2004	human stool	Y	Y	
6269	OXC412	UK [England]	2004	human stool	Y	N	

6281	OXC424	UK [England]	2004	human stool	Y	N
6310	OXC453	UK [England]	2004	human stool	Y	N
6326	OXC469	UK [England]	2004	human stool	Y	N
6415	OXC558	UK [England]	2004	human stool	N	N
6463	OXC606	UK [England]	2004	human stool	Y	N
12900	OXC6565	UK [England]	2011	human stool	N	N
12906	OXC6571	UK [England]	2011	human stool	Y	Y
14425	OXC2061	UK [England]	2006	human stool	Y	Y
14452	OXC2092	UK [England]	2006	human stool	Y	Y
14484	OXC2129	UK [England]	2006	human stool	Y	N
14575	OXC2284	UK [England]	2007	human stool	Y	N
14629	OXC2368	UK [England]	2007	human stool	N	N
14645	OXC2406	UK [England]	2007	human stool	Y	Y
14648	OXC2409	UK [England]	2007	human stool	Y	N
14660	OXC2422	UK [England]	2007	human stool	Y	N
14715	OXC2498	UK [England]	2007	human stool	Y	N
14727	OXC2512	UK [England]	2007	human stool	Y	N
14792	OXC2595	UK [England]	2007	human stool	Y	N
14803	OXC2608	UK [England]	2007	human stool	N	N
14856	OXC2677	UK [England]	2007	human stool	Y	Y
14908	OXC2746	UK [England]	2007	human stool	Y	N
14942	OXC2790	UK [England]	2007	human stool	Y	Y
15841	OXC4249	UK [England]	2009	human stool	Y	N
16063	OXC6266	UK [England]	2011	human stool	Y	Y
16074	OXC6277	UK [England]	2011	human stool	N	N
16083	OXC6286	UK [England]	2011	human stool	Y	Y
16086	OXC6289	UK [England]	2011	human stool	Y	Y
16089	OXC6292	UK [England]	2011	human stool	Y	Y
16123	OXC6326	UK [England]	2011	human stool	Y	N
16128	OXC6331	UK [England]	2011	human stool	Y	N
16144	OXC6347	UK [England]	2011	human stool	Y	N
16242	OXC6449	UK [England]	2011	human stool	Y	N
16249	OXC6457	UK [England]	2011	human stool	Y	Y
16251	OXC6459	UK [England]	2011	human stool	Y	N
16253	OXC6461	UK [England]	2011	human stool	Y	N
16281	OXC6489	UK [England]	2011	human stool	Y	N
16285	OXC6493	UK [England]	2011	human stool	Y	N
16294	OXC6502	UK [England]	2011	human stool	Y	N
16316	OXC6524	UK [England]	2011	human stool	Y	N
16319	OXC6527	UK [England]	2011	human stool	Y	N
16322	OXC6530	UK [England]	2011	human stool	Y	N
16323	OXC6531	UK [England]	2011	human stool	N	N
16335	OXC6543	UK [England]	2011	human stool	Y	N
16341	OXC6590	UK [England]	2011	human stool	Y	N
16349	OXC6598	UK [England]	2011	human stool	N	N
16351	OXC6600	UK [England]	2011	human stool	Y	N
16363	OXC6613	UK [England]	2011	human stool	Y	N

16365	OXC6615	UK [England]	2011	human stool	Y	N
16385	OXC6636	UK [England]	2011	human stool	Y	N
18221	OXC6664	UK [England]	2011	human stool	Y	N
18222	OXC6665	UK [England]	2011	human stool	Y	N
18223	OXC6666	UK [England]	2011	human stool	Y	N
18230	OXC6673	UK [England]	2011	human stool	Y	N
18234	OXC6677	UK [England]	2011	human stool	Y	N
18270	OXC6713	UK [England]	2011	human stool	Y	N
18271	OXC6714	UK [England]	2011	human stool	Y	N
18320	OXC6763	UK [England]	2011	human stool	Y	N
18377	OXC6820	UK [England]	2012	human stool	Y	N
18466	OXC4776	UK [England]	2009	human stool	Y	N
21106	OXC6932	UK [England]	2012	human stool	N	N
21120	OXC6946	UK [England]	2012	human stool	Y	N
21123	OXC6949	UK [England]	2012	human stool	Y	N
21130	OXC6956	UK [England]	2012	human stool	unk*	N
21138	OXC6964	UK [England]	2012	human stool	Y	Y
21165	OXC6994	UK [England]	2012	human stool	Y	N
21213	OXC7132	UK [England]	2012	human stool	Y	Y
21317	OXC5333	UK [England]	2010	human stool	Y	N
21319	OXC5335	UK [England]	2010	human stool	Y	Y
21329	OXC5349	UK [England]	2010	human stool	Y	Y
21353	OXC5393	UK [England]	2010	human stool	Y	N
21368	OXC5414	UK [England]	2010	human stool	Y	N
21381	OXC5435	UK [England]	2010	human stool	Y	Y
21389	OXC5445	UK [England]	2010	human stool	Y	N
21390	OXC5451	UK [England]	2010	human stool	Y	N
21398	OXC5462	UK [England]	2010	human stool	Y	N
21414	OXC5664	UK [England]	2010	human stool	Y	N
21434	OXC5691	UK [England]	2010	human stool	N	Y
21453	OXC5720	UK [England]	2010	human stool	Y	Y
21457	OXC5725	UK [England]	2010	human stool	Y	N
21461	OXC5731	UK [England]	2010	human stool	Y	N
21487	OXC5766	UK [England]	2011	human stool	Y	Y
21491	OXC5771	UK [England]	2011	human stool	Y	N
21555	OXC6836	UK [England]	2012	human stool	Y	N
21579	OXC6860	UK [England]	2012	human stool	Y	N
21580	OXC6861	UK [England]	2012	human stool	Y	N
21584	OXC6865	UK [England]	2012	human stool	Y	Y
21587	OXC6868	UK [England]	2012	human stool	Y	N
22095	OXC6900	UK [England]	2012	human stool	Y	Y
22117	OXC6922	UK [England]	2012	human stool	Y	N
22138	OXC4797	UK [England]	2010	human stool	Y	N
22141	OXC4801	UK [England]	2010	human stool	Y	N
22144	OXC4807	UK [England]	2010	human stool	Y	N
22182	OXC4877	UK [England]	2010	human stool	Y	N
22185	OXC4880	UK [England]	2010	human stool	Y	N

22202	OXC7015	UK [England]	2012	human stool	Y	N
22207	OXC7020	UK [England]	2012	human stool	Y	N
22215	OXC7029	UK [England]	2012	human stool	Y	N
22226	OXC7041	UK [England]	2012	human stool	Y	N
22249	OXC7065	UK [England]	2012	human stool	unk	Y
22255	OXC7071	UK [England]	2012	human stool	Y	N
22264	OXC7080	UK [England]	2012	human stool	Y	N
22288	OXC7246	UK [England]	2012	human stool	Y	Y
22301	OXC7260	UK [England]	2012	human stool	Y	N
22305	OXC7264	UK [England]	2012	human stool	Y	N
22311	OXC7270	UK [England]	2012	human stool	Y	Y
22334	OXC7293	UK [England]	2012	human stool	Y	N
22343	OXC7302	UK [England]	2012	human stool	Y	N
22346	OXC7305	UK [England]	2012	human stool	Y	N
22679	OXC7188	UK [England]	2012	human stool	Y	N
22705	OXC7214	UK [England]	2012	human stool	Y	Y
22711	OXC7220	UK [England]	2012	human stool	Y	N
23514	OXC5628	UK [England]	2010	human stool	Y	Y
23534	OXC5651	UK [England]	2010	human stool	Y	N
23549	OXC5799	UK [England]	2011	human stool	Y	N
23575	OXC5829	UK [England]	2011	human stool	Y	Y
23612	OXC5879	UK [England]	2011	human stool	Y	Y
23631	OXC5905	UK [England]	2011	human stool	Y	N
23632	OXC5906	UK [England]	2011	human stool	Y	N
23647	OXC5925	UK [England]	2011	human stool	Y	N
23648	OXC5926	UK [England]	2011	human stool	Y	N
23677	OXC5902	UK [England]	2011	human stool	Y	Y
23889	OXC7349	UK [England]	2012	human stool	Y	N
23901	OXC7362	UK [England]	2012	human stool	Y	N
23951	OXC7417	UK [England]	2012	human stool	Y	N
23961	OXC7428	UK [England]	2012	human stool	Y	N
24006	OXC7477	UK [England]	2012	human stool	Y	Y
24010	OXC7481	UK [England]	2012	human stool	Y	N
24016	OXC7488	UK [England]	2012	human stool	Y	N
24026	OXC7499	UK [England]	2012	human stool	Y	Y
24037	OXC7512	UK [England]	2012	human stool	Y	Y
24044	OXC4567	UK [England]	2009	human stool	Y	Y
24052	OXC4578	UK [England]	2009	human stool	Y	N
24061	OXC4590	UK [England]	2009	human stool	Y	N
24068	OXC4600	UK [England]	2009	human stool	Y	Y
24078	OXC4628	UK [England]	2009	human stool	N	N
24119	OXC7514	UK [England]	2012	human stool	Y	Y
24133	OXC7529	UK [England]	2012	human stool	Y	Y
24312	CJ6946_R	UK [England]			Y	N
24319	ARI_916_R	UK			Y	Y
24325	ARI_1130_R	UK			Y	N
24342	CJ7015_R	UK [England]			Y	N

24478	OXC7535	UK [England]	2012	human stool	Y	Y
24536	OXC7606	UK [England]	2012	human stool	Y	N
24542	OXC7613	UK [England]	2012	human stool	Y	N
24543	OXC7614	UK [England]	2012	human stool	Y	N
24556	OXC7627	UK [England]	2012	human stool	Y	N
24566	OXC7637	UK [England]	2012	human stool	Y	N
24568	OXC7639	UK [England]	2012	human stool	Y	Y
24608	OXC7810	UK [England]	2013	human stool	Y	N
24632	OXC7835	UK [England]	2013	human stool	Y	N
24633	OXC7836	UK [England]	2013	human stool	Y	N
24634	OXC7837	UK [England]	2013	human stool	Y	N
24642	OXC7845	UK [England]	2013	human stool	Y	N
24684	OXC5010	UK [England]	2010	human stool	N	N
24708	OXC5045	UK [England]	2010	human stool	Y	N
24711	OXC5049	UK [England]	2010	human stool	Y	Y
24733	OXC5094	UK [England]	2010	human stool	Y	N
24753	OXC5131	UK [England]	2010	human stool	Y	N
24770	OXC5169	UK [England]	2010	human stool	Y	N
24898	OXC7641	UK [England]	2012	human stool	N	N
24902	OXC7645	UK [England]	2012	human stool	Y	N
24912	OXC7657	UK [England]	2012	human stool	Y	N
24915	OXC7660	UK [England]	2012	human stool	Y	Y
24924	OXC7669	UK [England]	2012	human stool	Y	N
24925	OXC7670	UK [England]	2012	human stool	Y	Y
24943	OXC7695	UK [England]	2012	human stool	Y	N
24947	OXC7699	UK [England]	2012	human stool	Y	Y
24958	OXC7716	UK [England]	2013	human stool	Y	Y
24962	OXC7720	UK [England]	2013	human stool	Y	N
24981	OXC7746	UK [England]	2013	human stool	Y	N
24989	OXC7854	UK [England]	2013	human stool	Y	N
25001	OXC7866	UK [England]	2013	human stool	Y	Y
25002	OXC7867	UK [England]	2013	human stool	Y	N
25006	OXC7871	UK [England]	2013	human stool	Y	N
25093	OXC4930	UK [England]	2010	human stool	Y	N
25112	OXC7805	UK [England]	2013	human stool	Y	N
25117	OXC7930	UK [England]	2013	human stool	Y	N
25381	OXC7966	UK [England]	2013	human stool	Y	Y
25409	OXC8022	UK [England]	2013	human stool	Y	Y
25464	OXC8138	UK [England]	2013	human stool	Y	N
25478	OXC7958	UK [England]	2013	human stool	Y	N
25479	OXC7961	UK [England]	2013	human stool	Y	N
25513	OXC8063	UK [England]	2013	human stool	Y	N
25520	OXC8082	UK [England]	2013	human stool	Y	N
25527	OXC8099	UK [England]	2013	human stool	Y	N
25530	OXC8103	UK [England]	2013	human stool	Y	N
25569	OXC7949	UK [England]	2013	human stool	Y	N
25585	OXC8015	UK [England]	2013	human stool	Y	N

25645	OXC8183	UK [England]	2013	human stool	Y	N
25854	E60490	Luxembourg	2006	cattle	Y	N
26041	Dg370	UK		other animal	Y	Y
27866	OXC7714	UK [England]	2013	human stool	Y	Y
27868	OXC7740	UK [England]	2013	human stool	Y	N
27869	OXC7753	UK [England]	2013	human stool	Y	N
27884	OXC7804	UK [England]	2013	human stool	Y	N
27897	OXC8192	UK [England]	2013	human stool	Y	Y
27902	OXC8197	UK [England]	2013	human stool	Y	N
27929	OXC8227	UK [England]	2013	human stool	Y	N
27932	OXC8230	UK [England]	2013	human stool	Y	Y
27933	OXC8231	UK [England]	2013	human stool	Y	N
27952	OXC8254	UK [England]	2013	human stool	Y	Y
27955	OXC8257	UK [England]	2013	human stool	Y	Y
27960	OXC8264	UK [England]	2013	human stool	Y	N
27971	OXC8278	UK [England]	2013	human stool	Y	Y
27974	OXC8282	UK [England]	2013	human stool	N	N
27982	OXC8291	UK [England]	2013	human stool	N	N
27987	OXC8296	UK [England]	2013	human stool	Y	N
27992	OXC8301	UK [England]	2013	human stool	Y	N
27993	OXC8302	UK [England]	2013	human stool	Y	N
28011	OXC8320	UK [England]	2013	human stool	Y	N
28016	OXC8325	UK [England]	2013	human stool	Y	Y
28022	OXC8331	UK [England]	2013	human stool	Y	Y
28026	OXC8335	UK [England]	2013	human stool	Y	N
28033	OXC8342	UK [England]	2013	human stool	Y	N
28047	OXC8359	UK [England]	2013	human stool	Y	N
28054	OXC8367	UK [England]	2013	human stool	Y	N
28567	OXC8364	UK [England]	2013	human stool	Y	Y
28569	OXC8368	UK [England]	2013	human stool	Y	Y
28572	OXC8379	UK [England]	2013	human stool	Y	N
28574	OXC8381	UK [England]	2013	human stool	Y	N
28618	OXC8427	UK [England]	2013	human stool	unk	N
28646	OXC8455	UK [England]	2013	human stool	Y	N
28647	OXC8456	UK [England]	2013	human stool	Y	N
28648	OXC8457	UK [England]	2013	human stool	Y	N
28654	OXC8462	UK [England]	2013	human stool	N	N
28665	OXC8472	UK [England]	2013	human stool	Y	N
28671	OXC8478	UK [England]	2013	human stool	Y	N
28691	OXC8500	UK [England]	2013	human stool	Y	N
28692	OXC8501	UK [England]	2013	human stool	Y	N
28693	OXC8502	UK [England]	2013	human stool	Y	N
28705	OXC8514	UK [England]	2013	human stool	Y	N
28719	OXC8528	UK [England]	2013	human stool	Y	N
28729	OXC8539	UK [England]	2013	human stool	Y	N
28731	OXC8541	UK [England]	2013	human stool	N	N
28874	E110057	Luxembourg	2011	waters	Y	Y

28875	E120350	Luxembourg	2012	waters	Y	Y
28876	C101794	Luxembourg	2010	human stool	Y	Y
28878	C120949	Luxembourg	2012	chicken	Y	Y
28879	C121664	Luxembourg	2012	human stool	Y	Y
28880	C130520	Luxembourg	2013	cattle	Y	Y
28896	OXC8699	UK [England]	2014	human stool	Y	N
28903	OXC8564	UK [England]	2013	human stool	Y	N
28922	OXC8586	UK [England]	2013	human stool	Y	N
28942	OXC8614	UK [England]	2014	human stool	Y	N
28960	OXC8646	UK [England]	2014	human stool	Y	N
28972	OXC8658	UK [England]	2014	human stool	Y	N
28974	OXC8318R	UK [England]	2013	human stool	Y	Y
28981	OXC8405	UK [England]	2013	human stool	Y	N
28989	OXC8620	UK [England]	2014	human stool	Y	N
29000	OXC8665	UK [England]	2014	human stool	Y	N
29052	OXC8727	UK [England]	2014	human stool	Y	N
29067	OXC8405R	UK [England]	2013	human stool	Y	N
29309	OXC6106	UK [England]	2011	human stool	Y	Y
29453	H140940806	UK [England]	2014	human stool	Y	N
29625	OXC5409	UK [England]	2010	human stool	Y	Y
29681	OXC5935	UK [England]	2011	human stool	Y	N
30423	OXC9004	UK [England]	2014	human stool	Y	N
30424	OXC8884	UK [England]	2014	human stool	Y	Y
30434	OXC8743	UK [England]	2014	human stool	Y	N
30440	OXC8749	UK [England]	2014	human stool	Y	N
30472	OXC8783	UK [England]	2014	human stool	Y	N
30474	OXC8785	UK [England]	2014	human stool	Y	N
30540	OXC8854	UK [England]	2014	human stool	Y	Y
30553	OXC8867	UK [England]	2014	human stool	Y	N
30569	OXC8885	UK [England]	2014	human stool	Y	Y
30582	OXC8899	UK [England]	2014	human stool	Y	N
30584	OXC8901	UK [England]	2014	human stool	Y	Y
30595	OXC8912	UK [England]	2014	human stool	Y	Y
30605	OXC8922	UK [England]	2014	human stool	Y	N
30660	OXC8978	UK [England]	2014	human stool	Y	N
30700	OXC9021	UK [England]	2014	human stool	Y	Y
30711	OXC9032	UK [England]	2014	human stool	Y	N
30714	OXC9035	UK [England]	2014	human stool	Y	N
30757	OXC9078	UK [England]	2014	human stool	Y	N
30798	OXC9119	UK [England]	2014	human stool	Y	N
30812	OXC9133	UK [England]	2014	human stool	Y	N
30836	OXC9157	UK [England]	2014	human stool	Y	Y
30852	OXC9173	UK [England]	2014	human stool	Y	N
30855	OXC9176	UK [England]	2014	human stool	Y	Y
30861	OXC9182	UK [England]	2014	human stool	Y	Y
30870	OXC9191	UK [England]	2014	human stool	Y	N
30933	OXC9260	UK [England]	2014	human stool	Y	N

30975	OXC9302	UK [England]	2014	human stool	Y	N
31017	OXC8756	UK [England]	2014	human stool	Y	Y
31023	OXC8831	UK [England]	2014	human stool	Y	N
31052	OXC8966	UK [England]	2014	human stool	Y	N
31062	OXC8980	UK [England]	2014	human stool	Y	N
31066	OXC8984	UK [England]	2014	human stool	Y	N
31089	OXC8992	UK [England]	2014	human stool	Y	Y
31129	H144320568	UK [England]	2014	human stool	Y	N
31137	OXC9318	UK [England]	2014	human stool	Y	N
31147	OXC9328	UK [England]	2014	human stool	Y	N
31148	OXC9329	UK [England]	2014	human stool	Y	N
31149	OXC9330	UK [England]	2014	human stool	Y	N
31171	OXC9352	UK [England]	2014	human stool	Y	N
31194	OXC9375	UK [England]	2014	human stool	Y	N
31241	OXC9422	UK [England]	2015	human stool	Y	N
31259	OXC9440	UK [England]	2015	human stool	Y	N
31265	OXC9446	UK [England]	2015	human stool	Y	N
31272	OXC9453	UK [England]	2015	human stool	Y	N
31277	OXC9458	UK [England]	2015	human stool	Y	N
31279	OXC9460	UK [England]	2015	human stool	Y	N
31283	OXC9464	UK [England]	2015	human stool	Y	N
31296	OXC9477	UK [England]	2015	human stool	Y	N
31305	OXC9486	UK [England]	2015	human stool	Y	N
31312	OXC9493	UK [England]	2015	human stool	Y	N
31323	OXC9504	UK [England]	2015	human stool	Y	N
31326	OXC9507	UK [England]	2015	human stool	Y	N
31333	OXC9514	UK [England]	2015	human stool	Y	N
31356	OXC9537	UK [England]	2015	human stool	Y	N
31384	OXC9565	UK [England]	2015	human stool	Y	N
31387	OXC9568	UK [England]	2015	human stool	Y	N
31390	OXC9571	UK [England]	2015	human stool	Y	N
31397	OXC9578	UK [England]	2015	human stool	Y	N
31402	OXC9583	UK [England]	2015	human stool	Y	N
31403	OXC9584	UK [England]	2015	human stool	Y	N
31404	OXC9585	UK [England]	2015	human stool	Y	N
31418	OXC9599	UK [England]	2015	human stool	Y	N
31438	OXC9619	UK [England]	2015	human stool	Y	N
31439	OXC9620	UK [England]	2015	human stool	N	N
31461	OXC9642	UK [England]	2015	human stool	Y	N
31462	OXC9643	UK [England]	2015	human stool	Y	N
31478	OXC9659	UK [England]	2015	human stool	Y	N
31492	OXC9673	UK [England]	2015	human stool	Y	N
31566	H145020743	UK [England]	2014	human stool	Y	N
31862	5B	Denmark	2015		Y	N
31864	MP18	Denmark	2015		Y	N
32544	H150620634	UK [England]	2015	human stool	Y	N
32570	H150820519	UK	2015		Y	N

32573	H150820523	UK	2015		Y	N
32588	H150920714	UK [England]	2015	human stool	Y	Y
32590	H150920716	UK [England]	2015	human stool	Y	N
32596	H151020734	UK [England]	2015		Y	N
32707	H151160574	UK	2015		Y	N
32775	OXC9719	UK [England]	2015	human stool	Y	Y
32810	OXC9754	UK [England]	2015	human stool	Y	N
32853	OXC9797	UK [England]	2015	human stool	Y	Y
32854	OXC9798	UK [England]	2015	human stool	Y	Y
32855	OXC9799	UK [England]	2015	human stool	Y	N
32866	OXC9810	UK [England]	2015	human stool	Y	N
32893	OXC9837	UK [England]	2015	human stool	Y	Y
32901	OXC9845	UK [England]	2015	human stool	Y	N
32935	OXC9879	UK [England]	2015	human stool	Y	N
33086	H151220565	UK	2015		Y	N
33087	H151220566	UK	2015		Y	N
33096	H151340582	UK [England]	2015	human stool	Y	N
33133	OXC6187X	UK [England]	2011	human stool	Y	N
33278	RL15000562	UK			Y	N
33286	H151920575	UK [England]	2015	human stool	Y	N
33289	H151920579	UK [England]	2015	human stool	Y	Y
33914	H152040481	UK	2015		Y	Y
33917	H152040485	UK	2015		Y	Y
33989	H150460541	UK	2015		Y	N
34058	H152580498	UK	2015		Y	N
34061	H151020732	UK [England]	2015		Y	N
34149	H121820083	Jersey	2012	human stool	Y	Y
34152	H122580779	UK	2012		Y	N
34160	H133640222	UK [England]	2013	human stool	Y	N
34161	H133640223	UK [England]	2013	human stool	Y	N
34162	H133640225	UK [England]	2013	human stool	Y	N
34163	H133640226	UK [England]	2013	human stool	Y	N
34164	H133640228	UK [England]	2013	human stool	Y	N
34175	H140740343	UK [England]	2014	human stool	Y	N
34433	OXC9901	UK [England]	2015	human stool	Y	N
34481	OXC9949	UK [England]	2015	human stool	Y	N
34507	OXC9975	UK [England]	2015	human stool	Y	N
34511	OXC9979	UK [England]	2015	human stool	Y	N
35296	Cj2008-1025	France		human	Y	N
35302	Cj2008-831	France		human	Y	N
37259	Seal113	UK [Scotland]	2011	other animal	Y	N
37566	Seal174	UK [Scotland]	2011	other animal	Y	N
37569	Seal181	UK [Scotland]	2011	other animal	Y	N
37570	Seal183	UK [Scotland]	2011	other animal	Y	N
37590	Seal48	UK [Scotland]	2011	other animal	Y	N
37594	Seal56	UK [Scotland]	2011	other animal	Y	N
37606	Seal82	UK [Scotland]	2011	other animal	Y	N

37608	Seal87	UK [Scotland]	2011	other animal	Y	N
38423	ARI1491	UK [Scotland]	2012	human stool	Y	N
38427	ARI1495	UK [Scotland]	2012	human stool	Y	N
38439	ARI1507	UK [Scotland]	2012	human stool	Y	Y
38442	ARI1511	UK [Scotland]	2012	human stool	Y	N
38451	ARI1521	UK [Scotland]	2012	human stool	Y	N
38462	ARI1533	UK [Scotland]	2012	human stool	Y	Y
38486	ARI1562	UK [Scotland]	2012	human stool	Y	N
38490	ARI1566	UK [Scotland]	2012	human stool	Y	N
38491	ARI1567	UK [Scotland]	2012	human stool	Y	N
38506	ARI1583	UK [Scotland]	2012	human stool	Y	N
38508	ARI1585	UK [Scotland]	2012	human stool	Y	N
38529	ARI1607	UK [Scotland]	2012	human stool	Y	N
38574	ARI1664	UK [Scotland]	2012	human stool	Y	N
38616	ARI1713	UK [Scotland]	2012	human stool	Y	N
38654	ARI1757	UK [Scotland]	2012	human stool	Y	N
38684	ARI1790	UK [Scotland]	2012	human stool	Y	N
38706	ARI1814	UK [Scotland]	2012	human stool	Y	N
38708	ARI1816	UK [Scotland]	2012	human stool	Y	N
38716	ARI1824	UK [Scotland]	2012	human stool	Y	Y
38735	ARI1847	UK [Scotland]	2012	human stool	Y	Y
38761	ARI1875	UK [Scotland]	2012	human stool	Y	N
38780	ARI1894	UK [Scotland]	2012	human stool	Y	N
38781	ARI1895	UK [Scotland]	2012	human stool	Y	N
38782	ARI1896	UK [Scotland]	2012	human stool	Y	N
38812	ARI1927	UK [Scotland]	2012	human stool	Y	Y
38825	ARI1940	UK [Scotland]	2012	human stool	Y	Y
38833	ARI1949	UK [Scotland]	2012	human stool	Y	N
38872	ARI1991	UK [Scotland]	2012	human stool	Y	Y
38884	ARI2003_1	UK [Scotland]	2012	human stool	Y	Y
38896	ARI2015_1	UK [Scotland]	2012	human stool	Y	N
38935	ARI2054	UK [Scotland]	2012	human stool	Y	Y
38949	ARI2068_1	UK [Scotland]	2012	human stool	Y	N
38968	ARI2088_1	UK [Scotland]	2012	human stool	Y	N
38973	ARI2093_1	UK [Scotland]	2012	human stool	Y	N
38982	ARI2103_1	UK [Scotland]	2012	human stool	Y	N
38984	ARI2105_1	UK [Scotland]	2012	human stool	N	N
38985	ARI2106_1	UK [Scotland]	2012	human stool	Y	N
38994	ARI2115_1	UK [Scotland]	2012	human stool	Y	Y
39024	ARI2147	UK [Scotland]	2012	human stool	Y	N
39038	ARI2161_1	UK [Scotland]	2012	human stool	Y	N
39064	ARI2187	UK [Scotland]	2012	human stool	Y	N
39072	ARI2195	UK [Scotland]	2012	human stool	Y	N
39076	ARI2199	UK [Scotland]	2012	human stool	Y	Y
39082	ARI2205	UK [Scotland]	2012	human stool	Y	N
39089	ARI2212	UK [Scotland]	2012	human stool	Y	N
39095	ARI2218	UK [Scotland]	2012	human stool	Y	Y

39105	ARI2228	UK [Scotland]	2012	human stool	Y	N
39106	ARI2229	UK [Scotland]	2012	human stool	Y	N
39114	ARI2241	UK [Scotland]	2013	human stool	Y	N
39138	ARI2289	UK [Scotland]	2013	human stool	Y	N
39179	ARI2348	UK [Scotland]	2013	human stool	Y	Y
39184	ARI2355	UK [Scotland]	2013	human stool	Y	N
39197	ARI2371	UK [Scotland]	2013	human stool	Y	N
39200	ARI2376	UK [Scotland]	2013	human stool	Y	Y
39201	ARI2377	UK [Scotland]	2013	human stool	Y	Y
39212	ARI2392	UK [Scotland]	2013	human stool	Y	N
39219	ARI2399	UK [Scotland]	2013	human stool	Y	N
39268	ARI2455	UK [Scotland]	2013	human stool	Y	N
39274	ARI2464	UK [Scotland]	2013	human stool	Y	N
39280	ARI2471	UK [Scotland]	2013	human stool	Y	N
39286	ARI2481	UK [Scotland]	2013	human stool	Y	Y
39330	ARI2525	UK [Scotland]	2013	human stool	Y	N
39368	ARI2575	UK [Scotland]	2013	human stool	Y	N
39378	ARI2588	UK [Scotland]	2013	human stool	Y	N
39380	ARI2590	UK [Scotland]	2013	human stool	Y	N
39388	ARI2598	UK [Scotland]	2013	human stool	Y	N
39392	ARI2603	UK [Scotland]	2013	human stool	Y	N
39417	ARI2629	UK [Scotland]	2013	human stool	Y	N
39454	ARI2666	UK [Scotland]	2013	human stool	Y	N
39485	ARI2700	UK [Scotland]	2013	human stool	N	N
39488	ARI2703	UK [Scotland]	2013	human stool	Y	N
39493	ARI2708	UK [Scotland]	2013	human stool	Y	N
39494	ARI2709	UK [Scotland]	2013	human stool	Y	N
39506	ARI2722	UK [Scotland]	2013	human stool	Y	Y
39515	ARI2731	UK [Scotland]	2013	human stool	Y	Y
39521	ARI2737	UK [Scotland]	2013	human stool	Y	N
39527	ARI2743	UK [Scotland]	2013	human stool	Y	N
39546	ARI2762	UK [Scotland]	2013	human stool	Y	N
39551	ARI2767	UK [Scotland]	2013	human stool	Y	N
39573	ARI2789	UK [Scotland]	2013	human stool	Y	N
39588	ARI2804	UK [Scotland]	2013	human stool	Y	N
39637	ARI2853	UK [Scotland]	2013	human stool	Y	Y
39640	ARI2856	UK [Scotland]	2013	human stool	Y	N
39643	ARI2859	UK [Scotland]	2013	human stool	Y	Y
39659	ARI2877	UK [Scotland]	2013	human stool	Y	N
39670	ARI2888	UK [Scotland]	2013	human stool	Y	N
39691	ARI2910	UK [Scotland]	2013	human stool	Y	N
39698	ARI2917	UK [Scotland]	2013	human stool	Y	N
39707	ARI2926	UK [Scotland]	2013	human stool	Y	N
39737	B0050	UK [Scotland]	2013	cattle	Y	Y
39812	C0476	UK [Scotland]	2013	chicken	Y	Y
39816	C0481	UK [Scotland]	2013	chicken	Y	Y
39821	C0487	UK [Scotland]	2013	chicken	Y	Y

39827	C0496	UK [Scotland]	2013	chicken	N	N
39830	C0502	UK [Scotland]	2013	chicken	Y	Y
39832	C0504	UK [Scotland]	2013	chicken	Y	N
39843	C0523	UK [Scotland]	2013	turkey	Y	N
39875	C0584	UK [Scotland]	2013	chicken	Y	Y
39877	C0586	UK [Scotland]	2013	chicken	Y	Y
39882	C0592	UK [Scotland]	2013	chicken	Y	N
39897	C0607	UK [Scotland]	2013	chicken	Y	Y
39916	C0629	UK [Scotland]	2013	chicken	Y	Y
39927	C0647	UK [Scotland]	2013	chicken	Y	Y
39951	C0676	UK [Scotland]	2013	chicken	Y	N
39963	C0688	UK [Scotland]	2013	chicken	Y	N
39971	C0700	UK [Scotland]	2013	chicken	Y	N
39972	C0701	UK [Scotland]	2013	chicken	Y	N
39974	C0704	UK [Scotland]	2013	chicken	Y	N
40270	ARI2252	UK [Scotland]	2013	human stool	Y	N
40282	ARI2276	UK [Scotland]	2013	human stool	Y	N
40291	ARI2296	UK [Scotland]	2013	human stool	Y	N
40292	ARI2297	UK [Scotland]	2013	human stool	Y	N
40296	ARI2312	UK [Scotland]	2013	human stool	Y	N
40317	ARI2435	UK [Scotland]	2013	human stool	Y	N
40321	ARI2457	UK [Scotland]	2013	human stool	Y	N
40331	ARI2941	UK [Scotland]	2013	human stool	Y	N
40357	ARI2972	UK [Scotland]	2013	human stool	Y	N
40359	ARI2974	UK [Scotland]	2013	human stool	Y	N
40361	ARI2976	UK [Scotland]	2013	human stool	Y	N
40366	ARI2981	UK [Scotland]	2013	human stool	Y	N
40372	ARI2987	UK [Scotland]	2013	human stool	Y	N
40398	ARI3076	UK [Scotland]	2013	human stool	Y	N
40403	ARI3084	UK [Scotland]	2013	human stool	Y	N
40414	ARI3107	UK [Scotland]	2014	human stool	Y	N
40464	C0563	UK [Scotland]	2013	chicken	Y	Y
40487	C0751	UK [Scotland]	2013	chicken	Y	N
40490	C0754	UK [Scotland]	2013	chicken	Y	N
40491	C0755	UK [Scotland]	2013	chicken	Y	N
40493	C0757	UK [Scotland]	2013	chicken	Y	N
40507	C0778	UK [Scotland]	2013	turkey	Y	N
40513	C0788	UK [Scotland]	2013	turkey	Y	N
40514	C0789	UK [Scotland]	2013	turkey	Y	N
40515	C0790	UK [Scotland]	2013	turkey	Y	N
40516	C0791	UK [Scotland]	2013	turkey	Y	N
40517	C0792	UK [Scotland]	2013	turkey	Y	N
40518	C0793	UK [Scotland]	2013	turkey	Y	N
40519	C0795	UK [Scotland]	2013	turkey	N	N
40520	C0798	UK [Scotland]	2013	turkey	Y	N
40524	C0804	UK [Scotland]	2013	chicken	Y	Y
40525	C0805	UK [Scotland]	2013	turkey	Y	Y

40627	ARI3003	UK [Scotland]	2013	human stool	Y	Y
40632	ARI3009	UK [Scotland]	2013	human stool	Y	N
40651	ARI3032	UK [Scotland]	2013	human stool	Y	N
40670	ARI3053	UK [Scotland]	2013	human stool	Y	N
40671	ARI3054	UK [Scotland]	2013	human stool	Y	N
40674	ARI3057	UK [Scotland]	2013	human stool	Y	N
40694	ARI3097	UK [Scotland]	2014	human stool	Y	N
40701	ARI3113	UK [Scotland]	2014	human stool	Y	N
40721	ARI3133	UK [Scotland]	2014	human stool	Y	N
40723	ARI3135	UK [Scotland]	2014	human stool	Y	N
40738	ARI3150	UK [Scotland]	2014	human stool	Y	N
40739	ARI3151	UK [Scotland]	2014	human stool	Y	N
40753	ARI3165	UK [Scotland]	2014	human stool	Y	N
40755	ARI3167	UK [Scotland]	2014	human stool	Y	N
40761	ARI3173	UK [Scotland]	2014	human stool	Y	N
40763	ARI3175	UK [Scotland]	2014	human stool	Y	N
40781	ARI3193	UK [Scotland]	2014	human stool	Y	N
40786	ARI3198	UK [Scotland]	2014	human stool	Y	Y
40792	ARI3204	UK [Scotland]	2014	human stool	Y	N
40794	ARI3206	UK [Scotland]	2014	human stool	Y	N
40801	ARI3213	UK [Scotland]	2014	human stool	Y	N
40809	ARI3221	UK [Scotland]	2014	human stool	Y	N
40830	ARI3243	UK [Scotland]	2014	human stool	Y	N
40841	ARI3256	UK [Scotland]	2014	human stool	Y	N
40844	ARI3259	UK [Scotland]	2014	human stool	Y	N
40846	ARI3261	UK [Scotland]	2014	human stool	Y	N
40849	ARI3264	UK [Scotland]	2014	human stool	Y	N
40855	ARI3270	UK [Scotland]	2014	human stool	Y	N
40856	ARI3271	UK [Scotland]	2014	human stool	Y	N
40881	ARI3296	UK [Scotland]	2014	human stool	Y	N
40894	ARI3309	UK [Scotland]	2014	human stool	Y	N
40898	ARI3314	UK [Scotland]	2014	human stool	Y	N
40907	ARI3323	UK [Scotland]	2014	human stool	Y	N
41034	C0858	UK [Scotland]	2014	chicken	Y	N
41038	C0862	UK [Scotland]	2014	chicken	Y	N
41039	C0863	UK [Scotland]	2014	chicken	Y	N
41040	C0864	UK [Scotland]	2014	chicken	Y	N
41052	C0878	UK [Scotland]	2014	chicken	Y	N
41065	C0892	UK [Scotland]	2014	chicken	Y	N
41068	C0895	UK [Scotland]	2014	chicken	Y	N
41118	C0950	UK [Scotland]	2014	chicken	Y	N
41122	C0954	UK [Scotland]	2014	chicken	Y	N
41125	C0957	UK [Scotland]	2014	chicken	Y	N
41277	ARI3330	UK [Scotland]	2014	human stool	Y	N
41282	ARI3337	UK [Scotland]	2014	human stool	Y	N
41325	ARI3382	UK [Scotland]	2014	human stool	Y	N
41377	ARI3435	UK [Scotland]	2014	human stool	Y	Y

41403	ARI3471	UK [Scotland]	2014	human stool	Y	N
41406	ARI3475	UK [Scotland]	2014	human stool	Y	N
41431	ARI3508	UK [Scotland]	2014	human stool	Y	N
41442	ARI3520	UK [Scotland]	2014	human stool	Y	N
41481	ARI3563	UK [Scotland]	2014	human stool	Y	N
41486	ARI3569	UK [Scotland]	2014	human stool	Y	N
41507	ARI3591	UK [Scotland]	2014	human stool	Y	N
41511	ARI3595	UK [Scotland]	2014	human stool	Y	N
41518	ARI3602	UK [Scotland]	2014	human stool	Y	N
41640	C1002	UK [Scotland]	2014	chicken	Y	N
41644	C1011	UK [Scotland]	2014	chicken	Y	N
41649	C1017	UK [Scotland]	2014	chicken	Y	N
41656	C1025	UK [Scotland]	2014	chicken	Y	N
41675	C1049	UK [Scotland]	2014	chicken	Y	N
41681	C1056	UK [Scotland]	2014	chicken	Y	N
41702	C1084	UK [Scotland]	2014	chicken	Y	N
41715	C1099	UK [Scotland]	2014	chicken	Y	N
41743	C1133	UK [Scotland]	2014	turkey	Y	N
41760	C1168	UK [Scotland]	2014	chicken	Y	N
41827	S1176	UK [Scotland]	2014	sheep	Y	N
41883	ARI3478	UK [Scotland]	2014	human stool	Y	N
41889	ARI3512	UK [Scotland]	2014	human stool	Y	N
41908	ARI3621	UK [Scotland]	2014	human stool	Y	Y
41917	ARI3630	UK [Scotland]	2014	human stool	Y	N
41919	ARI3632	UK [Scotland]	2014	human stool	Y	Y
41947	ARI3660	UK [Scotland]	2014	human stool	Y	N
41983	ARI3696	UK [Scotland]	2014	human stool	Y	N
42005	ARI3718	UK [Scotland]	2014	human stool	Y	N
42014	ARI3727	UK [Scotland]	2014	human stool	Y	N
42016	ARI3729	UK [Scotland]	2014	human stool	Y	N
42019	ARI3732	UK [Scotland]	2014	human stool	Y	N
42060	ARI3774	UK [Scotland]	2015	human stool	Y	N
42065	ARI3779	UK [Scotland]	2015	human stool	Y	N
42066	ARI3780	UK [Scotland]	2015	human stool	Y	N
42074	ARI3789	UK [Scotland]	2015	human stool	Y	N
42076	ARI3792	UK [Scotland]	2015	human stool	Y	N
42080	ARI3796	UK [Scotland]	2015	human stool	Y	N
42084	ARI3800	UK [Scotland]	2015	human stool	Y	N
42112	ARI3828	UK [Scotland]	2015	human stool	Y	N
42115	ARI3831	UK [Scotland]	2015	human stool	Y	N
42119	ARI3835	UK [Scotland]	2015	human stool	Y	N
42121	ARI3837	UK [Scotland]	2015	human stool	Y	N
42122	ARI3838	UK [Scotland]	2015	human stool	Y	N
42124	ARI3840	UK [Scotland]	2015	human stool	Y	N
42127	ARI3843	UK [Scotland]	2015	human stool	Y	N
42129	ARI3845	UK [Scotland]	2015	human stool	Y	N
42130	ARI3846	UK [Scotland]	2015	human stool	Y	N

42151	ARI3869	UK [Scotland]	2015	human stool	Y	N
42152	ARI3870	UK [Scotland]	2015	human stool	Y	N
42153	ARI3871	UK [Scotland]	2015	human stool	Y	N
42177	C0474	UK [Scotland]	2013	chicken	Y	Y
42195	C1182	UK [Scotland]	2014	turkey	Y	Y
42208	C1211	UK [Scotland]	2014	chicken	Y	N
42230	C1253	UK [Scotland]	2014	chicken	Y	N
42257	C1297	UK [Scotland]	2015	chicken	Y	N
42269	C1322	UK [Scotland]	2015	chicken	Y	N
42277	C1334	UK [Scotland]	2015	chicken	Y	N
42285	C1345	UK [Scotland]	2015	turkey	Y	Y
42288	C1349	UK [Scotland]	2015	turkey	Y	Y
42295	C1358	UK [Scotland]	2015	chicken	Y	N
42299	C1362	UK [Scotland]	2015	chicken	Y	N
42300	C1363	UK [Scotland]	2015	chicken	Y	N
42314	C1387	UK [Scotland]	2015	chicken	Y	N
42334	C1408	UK [Scotland]	2015	chicken	Y	Y
42344	C1420	UK [Scotland]	2015	chicken	Y	N
42346	C1422	UK [Scotland]	2015	chicken	unk	N
42350	C1426	UK [Scotland]	2015	chicken	Y	N
42351	C1427	UK [Scotland]	2015	chicken	Y	N
42353	C1429	UK [Scotland]	2015	chicken	Y	N
42355	C1431	UK [Scotland]	2015	chicken	Y	N
42363	C1441	UK [Scotland]	2015	chicken	Y	N
42364	C1442	UK [Scotland]	2015	chicken	Y	N
42369	C1447	UK [Scotland]	2015	chicken	Y	N
42382	C1461	UK [Scotland]	2015	chicken	Y	N
42394	C1473	UK [Scotland]	2015	chicken	Y	N
42403	C1482	UK [Scotland]	2015	chicken	Y	Y
42420	C1499	UK [Scotland]	2015	chicken	Y	N
42427	C1506	UK [Scotland]	2015	chicken	Y	N
42447	C1526	UK [Scotland]	2015	chicken	Y	Y
42467	C1546	UK [Scotland]	2015	chicken	Y	N
42469	C1548	UK [Scotland]	2015	chicken	Y	N
42470	C1549	UK [Scotland]	2015	chicken	N	N
42471	C1550	UK [Scotland]	2015	chicken	N	N
42473	C1552	UK [Scotland]	2015	chicken	N	N
42554	ARI1419	UK [Scotland]	2011	human stool	Y	N
42563	ARI1428	UK [Scotland]	2012	human stool	N	Y
42569	ARI1435	UK [Scotland]	2012	human stool	Y	N
42574	ARI1441	UK [Scotland]	2012	human stool	N	N
42586	ARI1455	UK [Scotland]	2012	human stool	Y	N
42594	ARI1464	UK [Scotland]	2012	human stool	Y	N
42657	ARI0909	UK [Scotland]	2011	human stool	Y	N
42661	ARI0913	UK [Scotland]	2011	human stool	N	N
42664	ARI0916	UK [Scotland]	2011	human stool	Y	Y
42678	ARI0930	UK [Scotland]	2011	human stool	Y	N

42682	ARI0934	UK [Scotland]	2011	human stool	Y	N
42683	ARI0935	UK [Scotland]	2011	human stool	Y	N
42691	ARI0945	UK [Scotland]	2011	human stool	Y	N
42718	ARI0976	UK [Scotland]	2011	human stool	N	Y
42726	ARI0986	UK [Scotland]	2011	human stool	Y	N
42821	ARI1091	UK [Scotland]	2011	human stool	Y	N
42823	ARI1093	UK [Scotland]	2011	human stool	Y	N
42824	ARI1094	UK [Scotland]	2011	human stool	Y	N
42952	ARI1130	UK [Scotland]	2011	human stool	Y	N
42973	ARI1153	UK [Scotland]	2011	human stool	Y	N
42984	ARI1165	UK [Scotland]	2011	human stool	Y	N
42992	ARI1173	UK [Scotland]	2011	human stool	Y	N
42996	ARI1177	UK [Scotland]	2011	human stool	Y	N
43001	ARI1182	UK [Scotland]	2011	human stool	N	N
43023	ARI1205	UK [Scotland]	2011	human stool	Y	N
43027	ARI1209	UK [Scotland]	2011	human stool	Y	N
43043	ARI1227	UK [Scotland]	2011	human stool	Y	N
43071	ARI1256	UK [Scotland]	2011	human stool	Y	Y
43079	ARI1264	UK [Scotland]	2011	human stool	Y	N
43100	ARI1287	UK [Scotland]	2011	human stool	Y	N
43108	ARI1295	UK [Scotland]	2011	human stool	Y	N
43111	ARI1298	UK [Scotland]	2011	human stool	Y	Y
43142	ARI1330	UK [Scotland]	2011	human stool	Y	N
43145	ARI1333	UK [Scotland]	2011	human stool	Y	N
43146	ARI1334	UK [Scotland]	2011	human stool	Y	N
43152	ARI1340	UK [Scotland]	2011	human stool	Y	N
43177	ARI1365	UK [Scotland]	2011	human stool	Y	N
43295	ARI0872	UK [Scotland]	2011	human stool	Y	N
43299	ARI0877	UK [Scotland]	2011	human stool	Y	N
43306	ARI0884	UK [Scotland]	2011	human stool	Y	Y
43308	ARI0886	UK [Scotland]	2011	human stool	Y	N
43309	ARI0887	UK [Scotland]	2011	human stool	Y	N
43339	C0261	UK [Scotland]	2011	chicken	Y	N
43340	C0262	UK [Scotland]	2011	chicken	Y	N
43363	C0285	UK [Scotland]	2011	chicken	Y	N
43365	C0287	UK [Scotland]	2011	chicken	Y	N
43366	C0288	UK [Scotland]	2011	chicken	Y	N
43369	C0291	UK [Scotland]	2011	chicken	Y	N
43396	C0318	UK [Scotland]	2011	chicken	Y	N
43434	C0393	UK [Scotland]	2012	chicken	Y	N
43435	C0394	UK [Scotland]	2012	chicken	Y	N
43444	C0407	UK [Scotland]	2012	chicken	Y	N
43445	C0408	UK [Scotland]	2012	chicken	Y	N
43478	C0452	UK [Scotland]	2012	chicken	Y	N
43517	S0625	UK [Scotland]	2011	sheep	Y	N
43587	NWC10	UK [England]	2015	human stool	Y	N
43594	NWC3	UK [England]	2015	human stool	Y	N

43596	NWC2	UK [England]	2015	human stool	Y	Y
43603	OXC10002	UK [England]	2015	human stool	Y	N
43617	OXC10001	UK [England]	2015	human stool	Y	N
43644	NWC44	UK [England]	2015	human stool	Y	Y
43647	NWC58	UK [England]	2015	human stool	Y	Y
43650	NWC56	UK [England]	2015	human stool	Y	Y
43657	NWC36	UK [England]	2015	human stool	Y	N
43667	NWC66	UK [England]	2015	human stool	N	N
43672	NWC67	UK [England]	2015	human stool	N	N
43688	NWC71	UK [England]	2015	human stool	N	N
43692	OXC10026	UK [England]	2015	human stool	Y	N
43695	OXC10016	UK [England]	2015	human stool	Y	N
43738	NWC109	UK [England]	2015	human stool	Y	N
43784	OXC10071	UK [England]	2015	human stool	Y	N
43798	NWC723	UK [England]	2016	human stool	Y	Y
43802	ACP758	UK	2016	chicken	Y	N
43807	OXC10078	UK [England]	2015	human stool	Y	N
43810	OXC10082	UK [England]	2015	human stool	Y	N
43834	NWC87	UK [England]	2015	human stool	Y	N
43836	OXC10039	UK [England]	2015	human stool	Y	N
43838	NWC183	UK [England]	2016	human stool	Y	Y
43849	OXC10093	UK [England]	2015	human stool	Y	N
43866	NWC189	UK [England]	2016	human stool	Y	Y
43883	NWC181	UK [England]	2016	human stool	Y	N
43889	NWC173	UK [England]	2015	human stool	Y	Y
43890	NWC186	UK [England]	2016	human stool	Y	N
47259	FI38	Lithuania	2012	chicken	Y	N
47260	FI01	Estonia	2013	human	Y	N
47261	FI43	Lithuania	2012	chicken	Y	N
47262	FI32	Lithuania	2012	chicken	Y	N
47263	FI42	Lithuania	2012	chicken	Y	N
47264	FI46	Lithuania	2012	chicken	Y	N
47299	NWC211	UK [England]	2015	human stool	Y	N
47309	NWC213	UK [England]	2016	human stool	Y	N
47311	NWC197	UK [England]	2016	human stool	Y	Y
47314	NWC199	UK [England]	2016	human stool	Y	Y
47318	NWC212	UK [England]	2016	human stool	Y	Y
47323	NWC207	UK [England]	2015	human stool	Y	Y
47333	OXC10146	UK [England]	2016	human stool	Y	N
47345	OXC10149	UK [England]	2016	human stool	N	N
47350	NWC219	UK [England]	2016	human stool	Y	N
47362	NWC225	UK [England]	2016	human stool	Y	N
47516	APHAP50	UK		chicken	Y	N
47517	APHAP51	UK		chicken	Y	N
47519	APHAP53	UK		chicken	Y	N
47533	APHAP71	UK		chicken	Y	N
47612	APHAP140	UK		chicken	Y	N

47682	ACP780	UK	2016	chicken	N	Y
47689	NWC240	UK [England]	2016	human stool	Y	N
47694	NWC253	UK [England]	2016	human stool	Y	N
47705	NWC234	UK [England]	2016	human stool	Y	Y
47735	NWC301	UK [England]	2015	human stool	Y	N
47741	NWC286	UK [England]	2015	human stool	Y	Y
47750	NWC302	UK [England]	2015	human stool	Y	N
47752	OXC10173	UK [England]	2015	human stool	Y	N
47780	NWC297	UK [England]	2015	human stool	Y	Y
47787	OXC10168	UK [England]	2015	human stool	Y	N
47837	ACP191	UK	2016	chicken	Y	N
47851	ACP1089	UK	2016	chicken	Y	N
47863	NWC310	UK [England]	2016	human stool	Y	N
47870	NWC312	UK [England]	2016	human stool	Y	Y
47888	NWC320	UK [England]	2016	human stool	Y	Y
47889	NWC315	UK [England]	2016	human stool	Y	Y
47897	NWC325	UK [England]	2016	human stool	Y	Y
47916	NWC333	UK [England]	2016	human stool	Y	Y
47927	ACP657	UK	2016	chicken	Y	N
48278	ARI4142	UK [Scotland]	2015	human stool	Y	N
48287	ARI4155	UK [Scotland]	2015	human stool	Y	Y
48298	ARI4166	UK [Scotland]	2015	human stool	Y	Y
48299	ARI4167	UK [Scotland]	2015	human stool	Y	Y
48307	ARI4175	UK [Scotland]	2015	human stool	Y	Y
48314	ARI4182	UK [Scotland]	2015	human stool	N	Y
48322	ARI4190	UK [Scotland]	2015	human stool	Y	N
48334	ARI4202	UK [Scotland]	2015	human stool	Y	N
48355	ARI3955	UK [Scotland]	2015	human stool	Y	N
48402	ARI4066	UK [Scotland]	2015	human stool	Y	N
48438	ARI4134	UK [Scotland]	2015	human stool	Y	N
48439	ARI4135	UK [Scotland]	2015	human stool	Y	N
50125	NWC348	UK [England]	2016	human stool	Y	Y
50132	NWC357	UK [England]	2016	human stool	Y	N
50142	NWC369	UK [England]	2016	human stool	Y	N
50167	NWC380	UK [England]	2016	human stool	Y	N
50181	NWC401	UK [England]	2016	human stool	Y	N
50215	NWC386	UK [England]	2016	human stool	Y	N
50231	NWC448	UK [England]	2016	human stool	N	N
50237	NWC455	UK [England]	2016	human stool	Y	N
50268	NWC510	UK [England]	2016	human stool	Y	N
50274	NWC485	UK [England]	2016	human stool	Y	Y
50278	NWC515	UK [England]	2016	human stool	Y	N
50311	NWC518	UK [England]	2016	human stool	Y	N
50318	NWC505	UK [England]	2016	human stool	Y	N
50325	NWC466	UK [England]	2016	human stool	Y	Y
50327	NWC468	UK [England]	2016	human stool	Y	N
50341	OXC10259	UK [England]	2016	human stool	Y	N

50344	NWC479	UK [England]	2016	human stool	Y	N
50347	NWC486	UK [England]	2016	human stool	Y	Y
50411	APHAC63	UK		chicken	Y	N
50479	NWC584	UK [England]	2016	human stool	Y	N
50480	NWC580	UK [England]	2016	human stool	Y	N
50488	NWC544	UK [England]	2016	human stool	Y	N
50503	OXC10284	UK [England]	2016	human stool	N	N
50527	OXC10305	UK [England]	2016	human stool	Y	N
50530	OXC10307	UK [England]	2016	human stool	Y	N
50549	NWC563	UK [England]	2016	human stool	N	N
50550	NWC564	UK [England]	2016	human stool	Y	N
50563	OXC10314	UK [England]	2016	human stool	Y	Y
50570	NWC621	UK [England]	2016	human stool	Y	N
50575	NWC607	UK [England]	2016	human stool	Y	N
50608	OXC10318	UK [England]	2016	human stool	Y	N
50616	NWC651	UK [England]	2016	human stool	Y	N
50637	OXC10325	UK [England]	2016	human stool	Y	Y
50642	ACP1202	UK	2016	chicken	Y	Y
50688	NWC658	UK [England]	2016	human stool	Y	N
50694	ACP74	UK	2016	chicken	Y	Y
50714	ACP719	UK	2016	chicken	Y	N
50735	ACP146	UK	2016	chicken	Y	N
50746	NWC666	UK [England]	2016	human stool	Y	N
50749	OXC10328	UK [England]	2016	human stool	Y	Y
50750	OXC10329	UK [England]	2016	human stool	Y	N
50763	ACP709	UK	2016	chicken	Y	N
50768	ACP718	UK	2016	chicken	Y	N
50777	ACP740	UK	2016	chicken	Y	N
50873	ACP706	UK	2016	chicken	Y	N
50886	ACP466	UK	2016	chicken	Y	Y
50895	ACP492	UK	2016	chicken	Y	N
50898	ACP50	UK	2016	chicken	Y	Y
50901	ACP59	UK	2016	chicken	Y	Y
50989	NWC679	UK [England]	2016	human stool	Y	N
51020	ACP1068	UK	2016	chicken	Y	N
51023	ACP1083	UK	2016	chicken	Y	N
51073	ACP296	UK	2016	chicken	Y	N
51097	ACP328	UK	2016	chicken	Y	N
51117	ACP1124	UK	2016	chicken	Y	N
51123	ACP1132	UK	2016	chicken	Y	N
51127	ACP1140	UK	2016	chicken	Y	N
51145	ACP669	UK	2016	chicken	Y	N
51147	ACP619	UK	2016	chicken	Y	N
51186	ACP250	UK	2016	chicken	Y	N
51210	ACP278	UK	2016	chicken	Y	N
51221	ACP752	UK	2016	chicken	Y	Y
51241	ACP618	UK	2016	chicken	Y	N

51242	ACP673	UK	2016	chicken	Y	N
51264	ACP1023	UK	2016	chicken	N	Y
51267	ACP1029	UK	2016	chicken	Y	Y
51273	ACP620	UK	2016	chicken	Y	N
51334	ACP1199	UK	2016	chicken	Y	N
51364	ACP934	UK	2016	chicken	Y	Y
51365	ACP935	UK	2016	chicken	Y	Y
51383	OXC10355	UK [England]	2016	human stool	Y	N
51384	OXC10356	UK [England]	2016	human stool	Y	Y
51386	OXC10358	UK [England]	2016	human stool	Y	Y
51389	NWC683	UK [England]	2016	human stool	Y	N
51390	NWC684	UK [England]	2016	human stool	N	N
51403	NWC693	UK [England]	2016	human stool	Y	N
51404	NWC694	UK [England]	2016	human stool	Y	N
51424	ACP886	UK	2016	chicken	Y	N
51432	NWC699	UK [England]	2016	human stool	Y	N
51444	NWC711	UK [England]	2016	human stool	Y	N
51456	ACP1091	UK	2016	chicken	Y	Y
51460	ACP1287	UK	2016	chicken	Y	Y
51465	ACP256	UK	2016	chicken	Y	N
51481	ACP292	UK	2016	chicken	Y	N
51486	ACP1118	UK	2016	chicken	Y	N
51487	ACP757	UK	2016	chicken	Y	N
51490	ACP1284	UK	2016	chicken	Y	N
51510	ACP1282	UK	2016	chicken	Y	N
51514	ACP434	UK	2016	chicken	Y	N
51518	ACP665	UK	2016	chicken	Y	N
51531	ACP793	UK	2016	chicken	Y	N
51553	11_S10	Spain	2010	chicken	Y	N
51604	64_S62	Spain	2010	waters	Y	N
51656	RC291	UK	2011	chicken	Y	N
51697	ACP818	UK	2016	chicken	Y	Y
51709	OXC10378	UK [England]	2016	human stool	Y	N
51718	OXC10385	UK [England]	2016	human stool	Y	N
51720	NWC728	UK [England]	2016	human stool	Y	N
51744	ACP897	UK	2016	chicken	Y	N
51833	C1580	UK [Scotland]	2015	chicken	Y	Y
51852	ACP45	UK	2016	chicken	Y	N
51853	ACP46	UK	2016	chicken	Y	N
51854	ACP47	UK	2016	chicken	Y	Y
51856	ACP66	UK	2016	chicken	Y	N
51866	C1581	UK [Scotland]	2016	chicken	Y	Y
51867	C1582	UK [Scotland]	2016	chicken	Y	Y
51873	NWC743	UK [England]	2016	human stool	Y	N
51875	ACP1177	UK	2016	chicken	Y	N
56222	ARI4245	UK [Scotland]	2015	human stool	Y	Y
56233	ARI4256	UK [Scotland]	2015	human stool	Y	Y

56239	ARI4262	UK [Scotland]	2015	human stool	Y	Y
56242	ARI4265	UK [Scotland]	2015	human stool	Y	Y
56243	ARI4266	UK [Scotland]	2015	human stool	Y	Y
56248	ARI4271	UK [Scotland]	2015	human stool	Y	Y
56251	ARI4274	UK [Scotland]	2015	human stool	Y	Y
56265	ARI4290	UK [Scotland]	2016	human stool	Y	N
56280	ARI4306	UK [Scotland]	2016	human stool	Y	Y
56283	ARI4309	UK [Scotland]	2016	human stool	Y	Y
56284	ARI4310	UK [Scotland]	2016	human stool	Y	Y
56285	ARI4311	UK [Scotland]	2016	human stool	Y	Y
56293	ARI4316	UK [Scotland]	2016	human stool	Y	N
56318	ARI4342	UK [Scotland]	2016	human stool	Y	Y
56335	ARI4360	UK [Scotland]	2016	human stool	Y	Y
56338	ARI4364	UK [Scotland]	2016	human stool	Y	Y
56364	ARI4391	UK [Scotland]	2016	human stool	Y	Y
56370	ARI4397	UK [Scotland]	2016	human stool	Y	Y
56372	ARI4400	UK [Scotland]	2016	human stool	N	Y
56380	ARI4408	UK [Scotland]	2016	human stool	Y	N
56385	ARI4341	UK [Scotland]	2016	human stool	Y	N
56394	ARI4420	UK [Scotland]	2016	human stool	Y	N
56397	ARI4423	UK [Scotland]	2016	human stool	N	N
56408	ARI4435	UK [Scotland]	2016	human stool	N	N
56426	ARI4454	UK [Scotland]	2016	human stool	N	N
56445	ARI4473	UK [Scotland]	2016	human stool	N	N
56462	C1556	UK [Scotland]	2015	chicken	N	Y
56463	C1557	UK [Scotland]	2015	chicken	N	Y
56467	C1561	UK [Scotland]	2015	chicken	N	Y
56469	C1565	UK [Scotland]	2015	chicken	N	Y
56470	C1566	UK [Scotland]	2015	chicken	N	Y
56471	C1567	UK [Scotland]	2015	chicken	N	Y
56472	C1568	UK [Scotland]	2015	chicken	N	Y
56474	C1572	UK [Scotland]	2015	chicken	N	Y
56478	C1576	UK [Scotland]	2015	chicken	N	Y
56479	C1577	UK [Scotland]	2015	chicken	N	Y
56481	ARI4441	UK [Scotland]	2016	human stool	N	N
56520	NWC756	UK [England]	2016	human stool	N	N
56548	OXC10394	UK [England]	2016	human stool	N	N
56578	NWC784	UK [England]	2016	human stool	N	Y
56580	NWC786	UK [England]	2016	human stool	N	N
56602	C1583	UK [Scotland]	2016	chicken	N	Y
56604	C1585	UK [Scotland]	2016	chicken	N	Y
56606	C1587	UK [Scotland]	2016	chicken	N	Y
56607	C1605	UK [Scotland]	2016	chicken	N	Y
56609	C1613	UK [Scotland]	2016	chicken	N	Y
56611	C1621	UK [Scotland]	2016	chicken	N	Y
56612	C1590	UK [Scotland]	2016	chicken	N	Y
56614	C1591	UK [Scotland]	2016	chicken	N	Y

56616	C1592	UK [Scotland]	2016	chicken	N	Y
56618	C1593	UK [Scotland]	2016	chicken	N	Y
56619	C1637	UK [Scotland]	2016	chicken	N	Y
56620	C1594	UK [Scotland]	2016	chicken	N	Y
56621	C1641	UK [Scotland]	2016	chicken	N	Y
56624	C1596	UK [Scotland]	2016	chicken	N	Y
56625	C1646	UK [Scotland]	2016	chicken	N	Y
56626	C1597	UK [Scotland]	2016	chicken	N	Y
56628	C1598	UK [Scotland]	2016	chicken	N	Y
56630	C1599	UK [Scotland]	2016	chicken	N	Y
56632	C1600	UK [Scotland]	2016	chicken	N	Y
56639	C1672	UK [Scotland]	2016	chicken	N	Y
56641	C1673	UK [Scotland]	2016	chicken	N	Y
56642	C1606	UK [Scotland]	2016	chicken	N	Y
56646	C1610	UK [Scotland]	2016	chicken	N	Y
56647	C1611	UK [Scotland]	2016	chicken	N	Y
56648	C1612	UK [Scotland]	2016	chicken	N	Y
56649	C1614	UK [Scotland]	2016	chicken	N	Y
56658	C1624	UK [Scotland]	2016	chicken	N	Y
56662	C1630	UK [Scotland]	2016	chicken	N	Y
56663	C1631	UK [Scotland]	2016	chicken	N	Y
56664	C1632	UK [Scotland]	2016	chicken	N	Y
56665	C1634	UK [Scotland]	2016	chicken	N	Y
56668	C1639	UK [Scotland]	2016	chicken	N	Y
56669	C1640	UK [Scotland]	2016	chicken	N	Y
56670	C1642	UK [Scotland]	2016	chicken	N	Y
56671	C1643	UK [Scotland]	2016	chicken	N	N
56673	C1647	UK [Scotland]	2016	chicken	N	Y
56676	C1651	UK [Scotland]	2016	chicken	N	Y
56677	C1652	UK [Scotland]	2016	chicken	N	Y
56687	C1666	UK [Scotland]	2016	chicken	N	Y
56691	C1671	UK [Scotland]	2016	chicken	N	Y
56692	C1674	UK [Scotland]	2016	chicken	N	Y
56693	C1638	UK [Scotland]	2016	chicken	N	Y
56698	C1680	UK [Scotland]	2016	chicken	N	Y
56699	C1682	UK [Scotland]	2016	chicken	N	Y
56701	C1685	UK [Scotland]	2016	chicken	N	Y
56702	C1686	UK [Scotland]	2016	chicken	N	Y
56703	C1690	UK [Scotland]	2016	chicken	N	Y
56713	C1700	UK [Scotland]	2016	chicken	N	Y
56715	C1678	UK [Scotland]	2016	chicken	N	Y
56716	C1703	UK [Scotland]	2016	chicken	N	Y
56717	C1681	UK [Scotland]	2016	chicken	N	Y
56719	C1683	UK [Scotland]	2016	chicken	N	Y
56721	C1687	UK [Scotland]	2016	chicken	N	Y
56722	C1706	UK [Scotland]	2016	chicken	N	Y
56723	C1688	UK [Scotland]	2016	chicken	N	Y

56725	C1702	UK [Scotland]	2016	chicken	N	Y
56727	C1709	UK [Scotland]	2016	chicken	N	Y
56728	C1731	UK [Scotland]	2016	chicken	N	Y
56730	C1732	UK [Scotland]	2016	chicken	N	Y
56731	C1711	UK [Scotland]	2016	chicken	N	Y
56732	C1736	UK [Scotland]	2016	chicken	N	Y
56733	C1712	UK [Scotland]	2016	chicken	N	Y
56734	C1737	UK [Scotland]	2016	chicken	N	Y
56738	C1774	UK [Scotland]	2016	chicken	N	Y
56744	C1720	UK [Scotland]	2016	chicken	N	Y
56747	C1723	UK [Scotland]	2016	chicken	N	Y
56758	C1739	UK [Scotland]	2016	chicken	N	Y
56781	C1769	UK [Scotland]	2016	chicken	N	Y
56785	C1773	UK [Scotland]	2016	chicken	N	Y
56802	ARI4502	UK [Scotland]	2016	human stool	N	Y
56873	C1822	UK [Scotland]	2016	chicken	N	Y
56874	C1823	UK [Scotland]	2016	chicken	N	Y
56875	C1824	UK [Scotland]	2016	chicken	N	Y
56886	C1835	UK [Scotland]	2016	chicken	N	Y
56956	C1906	UK [Scotland]	2016	chicken	N	Y
56966	C1916	UK [Scotland]	2016	chicken	N	Y
56968	C1918	UK [Scotland]	2016	chicken	N	Y
56988	NWC804	UK [England]	2016	human stool	N	Y
57019	NWC826	UK [England]	2016	human stool	N	N
57025	NWC832	UK [England]	2016	human stool	N	N
57028	NWC795	UK [England]	2016	human stool	N	N
57043	APHAC125	UK		chicken	N	N
57065	APHAC147	UK		chicken	N	N
57102	APHAC186	UK		chicken	N	N
57221	NWC856	UK [England]	2016	human stool	N	N
57241	OXC10438	UK [England]	2016	human stool	N	N
57296	NWC901	UK [England]	2016	human stool	N	N
57349	OXC10479	UK [England]	2016	human stool	N	N
57367	NWC953	UK [England]	2016	human stool	N	N
57404	OXC10502	UK [England]	2016	human stool	N	Y
57652	OXC10513	UK [England]	2015	human stool	N	N
57769	chick1086	UK	2009	chicken	N	Y
57770	chick1360	UK	2009	chicken	N	Y
57783	chick50	UK	2009	chicken	N	Y
57867	SS_108	UK	2008	broiler environ	N	N
57868	SS_109	UK	2008	broiler environ	N	Y
57882	SS_139	UK	2008	chicken	N	Y
57893	SS_156	UK	2008	broiler environ	N	N
57910	SS_181	UK	2008	broiler environ	N	Y
57914	SS_187	UK	2009	chicken	N	Y
57918	SS_191	UK	2008	broiler environ	N	N
57938	SS_222	UK	2008	broiler environ	N	N

57971	SS_278	UK	2005	chicken	N	N
57982	SS_297	UK	2007	chicken	N	Y
58044	SS_592	UK	2012	human stool	N	N
58065	NWC1026	UK [England]	2016	human stool	N	N
58077	NWC1015	UK [England]	2016	human stool	N	Y
58083	OXC10538	UK [England]	2016	human stool	N	Y
58093	NWC1011	UK [England]	2017	human stool	N	N
58096	NWC1036	UK [England]	2016	human stool	N	N
58168	ARI4000	UK [Scotland]	2015	human stool	N	N
58183	ARI4022	UK [Scotland]	2015	human stool	N	N
58246	NWC1063	UK [England]	2016	human stool	N	N
58252	OXC10560	UK [England]	2016	human stool	N	N
58253	NWC1068	UK [England]	2016	human stool	N	Y
58264	NWC1075	UK [England]	2016	human stool	N	Y
58274	NWC1082	UK [England]	2016	human stool	N	N
58289	NWC1087	UK [England]	2016	human stool	N	N
58348	NWC1115	UK [England]	2016	human stool	N	Y
58349	NWC1116	UK [England]	2016	human stool	N	Y
58352	OXC10590	UK [England]	2016	human stool	N	N
58355	NWC1125	UK [England]	2016	human stool	N	N
58390	NWC1165	UK [England]	2016	human stool	N	Y
58437	OXC10624	UK [England]	2016	human stool	N	N
58441	NWC1171	UK [England]	2016	human stool	N	N
58515	ARI4533	UK [Scotland]	2016	human stool	N	N
58528	ARI4547	UK [Scotland]	2016	human stool	N	N
58532	ARI4551	UK [Scotland]	2016	human stool	N	Y
59004	NWC1193	UK [England]	2017	human stool	N	N
59011	NWC1198	UK [England]	2017	human stool	N	N
59015	OXC10648	UK [England]	2017	human stool	N	N
59029	OXC10651	UK [England]	2017	human stool	N	Y
59066	ARI4656	UK [Scotland]	2016	human stool	N	N
59085	ARI4675	UK [Scotland]	2016	human stool	N	N
59096	ARI4686	UK [Scotland]	2016	human stool	N	N
59151	ARI4087	UK [Scotland]	2015	human stool	N	N
59166	ARI4108	UK [Scotland]	2015	human stool	N	N
59216	ARI4283	UK [Scotland]	2016	human stool	N	Y
59218	ARI4292	UK [Scotland]	2016	human stool	N	Y
59243	C1558	UK [Scotland]	2015	chicken	N	Y
59303	ARI4650	UK [Scotland]	2016	human stool	N	N
59321	ARI3910	UK [Scotland]	2015	human stool	N	N
59343	ARI4592	UK [Scotland]	2016	human stool	N	Y
59414	NWC1235	UK [England]	2016	human stool	N	Y
59423	OXC10684	UK [England]	2016	human stool	N	N
59478	OXC10754	UK [England]	2017	human stool	N	N
59496	NWC1367	UK [England]	2017	human stool	N	N
59500	NWC1339	UK [England]	2017	human stool	N	N
59507	OXC10744	UK [England]	2017	human stool	N	N

59515	NWC1348	UK [England]	2017	human stool	N	N
59551	NWC1283	UK [England]	2017	human stool	N	N
59552	NWC1284	UK [England]	2017	human stool	N	N
59557	OXC10725	UK [England]	2017	human stool	N	N
59564	OXC10726	UK [England]	2017	human stool	N	N
59567	NWC1294	UK [England]	2017	human stool	N	N
59568	NWC1295	UK [England]	2017	human stool	N	Y
59577	NWC1305	UK [England]	2017	human stool	N	N
59594	NWC1300	UK [England]	2017	human stool	N	Y
59595	NWC1301	UK [England]	2017	human stool	N	Y
59602	OXC10703	UK [England]	2016	human stool	N	N
59603	OXC10715	UK [England]	2017	human stool	N	N
59607	NWC1271	UK [England]	2017	human stool	N	Y
59611	NWC1274	UK [England]	2017	human stool	N	N
59616	NWC1279	UK [England]	2017	human stool	N	N
59636	OXC10706	UK [England]	2016	human stool	N	N
59640	OXC10695	UK [England]	2016	human stool	N	N
59644	NWC1259	UK [England]	2016	human stool	N	N
59662	NWC1383	UK [England]	2017	human stool	N	N
59672	OXC10774	UK [England]	2017	human stool	N	N
59673	OXC10775	UK [England]	2017	human stool	N	N
59694	NWC1397	UK [England]	2017	human stool	N	N
59731	NWC1430	UK [England]	2017	human stool	N	N
59742	NWC1411	UK [England]	2017	human stool	N	N
59744	OXC10792	UK [England]	2017	human stool	N	N
59753	NWC1417	UK [England]	2017	human stool	N	Y
59761	NWC1422	UK [England]	2017	human stool	N	N
59766	NWC1424	UK [England]	2017	human stool	N	N
59783	OXC10819	UK [England]	2017	human stool	N	N
59784	OXC10827	UK [England]	2017	human stool	N	N
59786	OXC10820	UK [England]	2017	human stool	N	Y
59793	NWC1435	UK [England]	2017	human stool	N	N
59808	NWC1439	UK [England]	2017	human stool	N	N
60436	508_3754	UK	2009	chicken	N	N
60437	507-0761	UK	2009	chicken	N	Y
60441	508-2543	UK	2009	chicken	N	Y
60442	508-2574	UK	2009	chicken	N	Y
60637	chick19	UK	2009	chicken	N	N
60902	CJ003CC21	Finland	1998	human blood	N	N
60903	CJ035CC21	Finland	2003	human blood	N	N
60904	CJ098CC21	Finland	2001	human blood	N	N
60906	CJ069CC21	Finland	2000	human blood	N	Y
60907	CJ502CC21	Finland	2006	human stool	N	N
60908	CJ505CC21	Finland	2006	human stool	N	Y
60909	CJ006CC21	Finland	2001	human blood	N	N
60912	CJ076CC21	Finland	2005	human blood	N	Y
60913	CJ500CC21	Finland	2006	human stool	N	Y

60914	CJ501CC21	Finland	2006	human stool	N	Y
60915	CJ503CC21	Finland	2006	human stool	N	N
60916	CJ504CC21	Finland	2006	human stool	N	Y
60917	CJ506CC21	Finland	2006	human stool	N	N
61091	OXC10882	UK [England]	2017	human stool	N	N
61096	NWC1467	UK [England]	2017	human stool	N	N
61098	NWC1468	UK [England]	2017	human stool	N	N
61102	OXC10883	UK [England]	2017	human stool	N	Y
61118	OXC10884	UK [England]	2017	human stool	N	N
61142	OXC10858	UK [England]	2017	human stool	N	N
61175	OXC10875	UK [England]	2017	human stool	N	N
61180	OXC10850	UK [England]	2017	human stool	N	N
61181	OXC10851	UK [England]	2017	human stool	N	N
61183	OXC10852	UK [England]	2017	human stool	N	N
61185	NWC1512	UK [England]	2017	human stool	N	N
61193	NWC1498	UK [England]	2017	human stool	N	N
61229	OXC10907	UK [England]	2017	human stool	N	N
61262	NWC1518	UK [England]	2017	human stool	N	N
61293	NWC1551	UK [England]	2017	human stool	N	N
61304	NWC1534	UK [England]	2017	human stool	N	N
61305	NWC1541	UK [England]	2017	human stool	N	N
61314	NWC1544	UK [England]	2017	human stool	N	N
61329	NWC1576	UK [England]	2017	human stool	N	N
61336	NWC1577	UK [England]	2017	human stool	N	N
61409	FDI14	UK	2015	chicken	N	N
61413	FDI18	UK	2015	chicken	N	Y
61438	FDI41	UK	2016	chicken	N	Y
61449	FDI51	UK	2016	chicken	N	N
61499	FDI79	UK	2015	chicken	N	N
61505	FDI128	UK	2016	chicken	N	Y
61514	FDI137	UK	2016	chicken	N	Y
61533	FDI124	UK	2015	chicken	N	N
61561	FDI179	UK	2015	chicken	N	N
61575	FDI193	UK	2015	chicken	N	N
61577	FDI195	UK	2015	chicken	N	Y
61582	FDI155	UK	2016	chicken	N	Y
61583	FDI156	UK	2016	chicken	N	Y
61597	FDI198	UK	2015	chicken	N	N
61599	FDI200	UK	2015	chicken	N	N
61601	FDI169	UK	2015	chicken	N	Y
61602	FDI170	UK	2015	chicken	N	Y
61626	FDI238	UK	2016	chicken	N	Y
61635	FDI248	UK	2015	chicken	N	N
61646	FDI205	UK	2015	chicken	N	N
61648	FDI207	UK	2015	chicken	N	N
61660	FDI295	UK	2017	lamb	N	N
61682	FDI264	UK	2016	chicken	N	N

61733	FDI343	UK	2015	chicken	N	N
62368	AC0745	France		chicken	N	Y
62378	AC3201	France		chicken	N	N
62442	CTA093	France		other animal	N	N
62471	CTA693	France		other animal	N	N
62607	NWC1635	UK [England]	2017	human stool	N	N
62608	OXC11005	UK [England]	2017	human stool	N	N
62609	NWC1641	UK [England]	2017	human stool	N	N
62622	NWC1645	UK [England]	2017	human stool	N	Y
62645	NWC1666	UK [England]	2017	human stool	N	N
62673	NWC1710	UK [England]	2017	human stool	N	Y
62743	NWC1774	UK [England]	2017	human stool	N	N
62751	NWC1786	UK [England]	2017	human stool	N	N
62756	OXC11093	UK [England]	2017	human stool	N	N
62758	NWC1782	UK [England]	2017	human stool	N	N
62767	NWC1816	UK [England]	2017	human stool	N	Y
62770	NWC1799	UK [England]	2017	human stool	N	N
62775	OXC11117	UK [England]	2017	human stool	N	N
62776	NWC1801	UK [England]	2017	human stool	N	N
62779	NWC1802	UK [England]	2017	human stool	N	N
62781	NWC1791	UK [England]	2017	human stool	N	N
62789	NWC1787	UK [England]	2017	human stool	N	N
62790	OXC11107	UK [England]	2017	human stool	N	N
62793	OXC11106	UK [England]	2017	human stool	N	Y
62794	OXC11103	UK [England]	2017	human stool	N	N
62797	NWC1796	UK [England]	2017	human stool	N	Y
62798	NWC1811	UK [England]	2017	human stool	N	N
62799	NWC1793	UK [England]	2017	human stool	N	N
62810	OXC11123	UK [England]	2017	human stool	N	N
62815	OXC11126	UK [England]	2017	human stool	N	N
62819	OXC11122	UK [England]	2017	human stool	N	N
62828	OXC11121	UK [England]	2017	human stool	N	Y
62897	OXC11176	UK [England]	2017	human stool	N	N
62925	NWC1879	UK [England]	2017	human stool	N	N
63411	F50	UK [Scotland]	2001	chicken	N	N
63507	F1202	UK [Scotland]	2001	human stool	N	N
63525	F1244	UK [Scotland]	2001	human stool	N	N
63528	F1258	UK [Scotland]	2001	human stool	N	Y
63603	F1131	UK [Scotland]	2001	human stool	N	N
63606	F1135	UK [Scotland]	2001	human stool	N	N
63658	3316	UK [Scotland]	2006	human stool	N	Y
63671	1929	UK [Scotland]	2005	human stool	N	N
63779	985	UK [Scotland]	2005	human stool	N	N
63780	986	UK [Scotland]	2005	human stool	N	N
63900	3928	UK [Scotland]	2006	human stool	N	N
63906	3984	UK [Scotland]	2006	human stool	N	N
63917	4458	UK [Scotland]	2006	human stool	N	N

63922	4607	UK [Scotland]	2006	human stool	N	N
63936	4907	UK [Scotland]	2006	human stool	N	N
63941	5034	UK [Scotland]	2006	human stool	N	N
63943	5117	UK [Scotland]	2006	human stool	N	Y
63944	5119	UK [Scotland]	2006	human stool	N	Y
63960	ARI0674	UK [Scotland]	2011	human stool	N	N
64027	ARI0787	UK [Scotland]	2011	human stool	N	N
64029	ARI0744	UK [Scotland]	2011	human stool	N	N
64040	OXC11391	UK [England]	2018	human stool	N	N
69226	NWC2055	UK [England]	2017	human stool	N	N
69239	NWC2065	UK [England]	2017	human stool	N	N
69297	OXC11315	UK [England]	2017	human stool	N	Y
69323	OXC11287	UK [England]	2017	human stool	N	N
69324	OXC11278	UK [England]	2017	human stool	N	N
69326	NWC2011	UK [England]	2017	human stool	N	N
69327	NWC2000	UK [England]	2017	human stool	N	N
69328	OXC11277	UK [England]	2017	human stool	N	N
69340	NWC2002	UK [England]	2017	human stool	N	N
69343	NWC2013	UK [England]	2017	human stool	N	N
69345	NWC2001	UK [England]	2017	human stool	N	N
69353	NWC2010	UK [England]	2017	human stool	N	N
69359	NWC2014	UK [England]	2017	human stool	N	Y
69361	NWC2004	UK [England]	2017	human stool	N	Y
69364	OXC11285	UK [England]	2017	human stool	N	N
69373	OXC11249	UK [England]	2017	human stool	N	N
69423	OXC11243	UK [England]	2017	human stool	N	Y
69428	NWC1971	UK [England]	2017	human stool	N	N
69435	OXC11247	UK [England]	2017	human stool	N	N
69460	NWC1963	UK [England]	2017	human stool	N	N
69479	OXC11213	UK [England]	2017	human stool	N	N
69484	OXC11226	UK [England]	2017	human stool	N	Y
69508	NWC1932	UK [England]	2017	human stool	N	N
69511	OXC11209	UK [England]	2017	human stool	N	N
69659	OXC11280	UK [England]	2017	human stool	N	N
69670	OXC11208	UK [England]	2017	human stool	N	Y
69676	NWC1984	UK [England]	2017	human stool	N	N
69840	NWC2119	UK [England]	2017	human stool	N	Y
69846	NWC2101	UK [England]	2017	human stool	N	Y
69860	NWC2084	UK [England]	2017	human stool	N	N
69881	NWC2105	UK [England]	2017	human stool	N	N
69882	NWC2093	UK [England]	2017	human stool	N	Y
69894	OXC11365	UK	2017	human stool	N	N
69898	NWC2134	UK [England]	2017	human stool	N	N
69902	OXC11374	UK [England]	2017	human stool	N	N
69965	FDI375	UK	2017	beef	N	N
70022	OXC11414	UK [England]	2018	human stool	N	N
70047	NWC2177	UK [England]	2017	human stool	N	Y

70048	NWC2178	UK [England]	2017	human stool	N	Y
70053	OXC11410	UK [England]	2017	human stool	N	N
70075	NWC2206	UK [England]	2018	human stool	N	N
70097	OXC11436	UK [England]	2018	human stool	N	N
70100	OXC11438	UK [England]	2017	human stool	N	N
70106	NWC2227	UK [England]	2018	human stool	N	N
70108	NWC2226	UK [England]	2018	human stool	N	N
70110	FDI452	UK	2018	beef	N	N
70113	NWC2224	UK [England]	2018	human stool	N	N
70116	OXC11441	UK [England]	2018	human stool	N	N
70126	NWC2229	UK [England]	2018	human stool	N	N
70127	NWC2222	UK [England]	2018	human stool	N	N
70128	OXC11439	UK [England]	2018	human stool	N	N
70137	OXC11433	UK [England]	2018	human stool	N	N
70165	NWC2232	UK [England]	2018	human stool	N	Y
70560	NWC2273	UK [England]	2018	human stool	N	N
70663	NWC2249	UK [England]	2018	human stool	N	N
70687	NWC2262	UK [England]	2018	human stool	N	N
70693	NWC2255	UK [England]	2018	human stool	N	N
70761	NWC2325	UK [England]	2018	human stool	N	N
70763	OXC11518	UK [England]	2018	human stool	N	N
70807	OXC11509	UK [England]	2018	human stool	N	N
70813	NWC2352	UK [England]	2018	human stool	N	N
70814	OXC11537	UK [England]	2018	human stool	N	N
70868	NWC2355	UK [England]	2018	human stool	N	N
70870	OXC11538	UK [England]	2018	human stool	N	N
70877	2-5	UK	2014	human stool	N	N
70878	2-10	UK	2014	human stool	N	N
70885	4-52	UK	2014	human stool	N	N
70886	4-51	UK	2014	human stool	N	N
70891	2-9	UK	2014	human stool	N	N
70899	D1-81	UK	2014	human stool	N	N
70900	D2-11	UK	2014	human stool	N	N
70903	D2-36	UK	2014	human stool	N	N
70904	D2-9	UK	2014	human stool	N	N
70905	D3-17	UK	2014	human stool	N	Y
70909	D4-51	UK	2014	human stool	N	N
70914	D4-7	UK	2014	human stool	N	N
70916	D4-79	UK	2014	human stool	N	N
70919	D5-80	UK	2014	human stool	N	N
70923	D6-57	UK	2014	human stool	N	N
70930	D7-62	UK	2014	human stool	N	N
70943	Po_1	Finland	2002	human stool	N	N
70944	Po_2	Finland	2002	human stool	N	N
70945	Ma_1	Finland	2002	cows milk	N	N
70946	Ma_B	Finland	2003	cows milk	N	N
70947	Le_204R	Finland	2003	cattle	N	N

70948	Le_755	Finland	2003	cattle	N	N
76522	OXC11551	UK	2016	human stool	N	N
76523	NWC2374	UK [England]	2016	human stool	N	N
76528	OXC11555	UK	2016	human stool	N	N
76535	NWC2385	UK [England]	2018	human stool	N	N
76579	NWC2404	UK [England]	2018	human stool	N	N
76581	NWC2398	UK [England]	2018	human stool	N	N
76597	NWC2430	UK [England]	2018	human stool	N	N
76608	NWC2419	UK [England]	2018	human stool	N	N
76667	NWC2508	UK [England]	2018	human stool	N	N
76679	OXC11646	UK [England]	2018	human stool	N	N
76689	NWC2502	UK [England]	2018	human stool	N	Y
76701	NWC2491	UK [England]	2018	human stool	N	N
76725	OXC11654	UK [England]	2018	human stool	N	N
76743	NWC2520	UK [England]	2018	human stool	N	N
76754	NWC2527	UK [England]	2018	human stool	N	N
76808	OXC11673	UK [England]	2018	human stool	N	Y
76814	NWC2579	UK [England]	2018	human stool	N	N
76823	NWC2580	UK [England]	2018	human stool	N	Y
76843	NWC2570	UK [England]	2018	human stool	N	N
76847	NWC2577	UK [England]	2016	human stool	N	N
76855	NWC2578	UK [England]	2018	human stool	N	N
76856	NWC2575	UK [England]	2018	human stool	N	N
76858	OXC11705	UK [England]	2018	human stool	N	N
76902	FDI580	UK	2018	chicken	N	N
76950	OXC11754	UK [England]	2018	human stool	N	Y
76954	NWC2640	UK [England]	2016	human stool	N	N
76980	NWC2651	UK [England]	2018	human stool	N	N
76992	NWC2663	UK [England]	2018	human stool	N	N
77003	NWC2679	UK [England]	2018	human stool	N	Y
77008	OXC11774	UK [England]	2018	human stool	N	N
77026	OXC11785	UK [England]	2018	human stool	N	N
77103	NWC2725	UK [England]	2016	human stool	N	N
77118	FDI603	UK	2017	chicken	N	N
77119	OXC11816	UK [England]	2018	human stool	N	N
77145	NWC2737	UK [England]	2018	human stool	N	N
77158	OXC11844	UK [England]	2018	human stool	N	N
77159	NWC2756	UK [England]	2018	human stool	N	N
77174	NWC2765	UK [England]	2018	human stool	N	Y
77225	NWC2811	UK [England]	2018	human stool	N	Y
77235	OXC11877	UK [England]	2018	human stool	N	N
77259	NWC2442	UK [England]	2018	human stool	N	Y
77279	NWC2446	UK [England]	2018	human stool	N	N
77283	NWC2453	UK [England]	2018	human stool	N	N
77285	OXC11618	UK [England]	2018	human stool	N	N
77288	NWC2456	UK [England]	2018	human stool	N	N
77290	OXC11612	UK [England]	2018	human stool	N	N

77291	NWC2448	UK [England]	2018	human stool	N	N
77298	OXC11616	UK [England]	2018	human stool	N	N
77445	WTCHG_507474_201107	UK			N	N
77447	OXC2089	UK	2006	human stool	N	Y
77474	OXC2062	UK	2006	human stool	N	Y
77530	PHEHIST005	UK	1997	human stool	N	N
77608	PHEHIST045	UK	1997	human stool	N	N
77676	PHEHIST094	UK	1997	human stool	N	Y
77678	PHEHIST096	UK	1997	human stool	N	N
77753	PHEHIST163	UK	1997	human stool	N	N
77763	PHEHIST171	UK	1997	human stool	N	N
77768	PHEHIST175	UK	1997	human stool	N	N
77817	PHEHIST215	UK		human stool	N	N
77909	PHEHIST291	UK	1997	human stool	N	N
77923	PHEHIST304	UK	1997	human stool	N	Y
77965	OXC155	UK [England]	2003	human stool	N	Y
77970	OXC53	UK [England]	2003	human stool	N	Y
77974	PHEHIST339	UK	1997	human stool	N	N
77981	PHEHIST346	UK	1997	human stool	N	Y
78014	OXC30	UK [England]	2003	human stool	N	Y
78040	PHEHIST395	UK	1998	human stool	N	Y
78137	NWC2816	UK [England]	2018	human stool	N	N
78162	OXC11893	UK [England]	2018	human stool	N	N
78166	NWC2827	UK [England]	2018	human stool	N	N
78171	OXC11898	UK [England]	2018	human stool	N	N
78175	OXC11896	UK [England]	2018	human stool	N	N
78225	FDI697	UK	2018	chicken	N	N
78235	FDI704	UK	2017	chicken	N	N
78251	NWC2838	UK [England]	2018	human stool	N	N
78259	NWC2846	UK [England]	2018	human stool	N	N
78261	NWC2851	UK [England]	2018	human stool	N	N
78266	FDI719	UK	2018	lamb	N	N
78270	OXC11920	UK [England]	2018	human stool	N	N
78348	OXC11942	UK [England]	2018	human stool	N	N
78389	OXC11937	UK [England]	2018	human stool	N	N
78391	NWC2880	UK [England]	2018	human stool	N	Y
78412	FDI784	UK	2018	chicken	N	N
78442	OXC11957	UK [England]	2018	human stool	N	N
78443	FDI790	UK	2018	chicken	N	N
78452	NWC2898	UK [England]	2018	human stool	N	N
78458	FDI812	UK	2018	chicken	N	N
78466	FDI814	UK	2018	chicken	N	N
78473	FDI829	UK	2018	chicken	N	N
78871	PHEHIST464	UK	1998	human stool	N	N
79837	RL16000343	UK [England]	2016	chicken	N	N
79849	RL16003794	UK [England]	2016	chicken	N	N
79980	OXC286	UK			N	Y

80227	14S	Poland		human stool	N	N
80239	37S	Poland		human stool	N	N
80293	RL17000157	UK [England]	2017	chicken	N	N
107059	8359	Finland	2014	wild bird	N	N
107060	8360	Finland	2014	wild bird	N	Y
108789	6H	Estonia	2018	human	N	N
108827	7.17H	Estonia	2017	human	N	N
110009	LNS1271330	Luxembourg	2020	human stool	N	N
110016	LNS1881298	Luxembourg	2019	human stool	N	N
110024	LNS2680632	Luxembourg	2020	human stool	N	N
110031	LNS3878735	Luxembourg	2018	human stool	N	N
110052	LNS4808088	Luxembourg	2019	human stool	N	N
110054	LNS4847690	Luxembourg	2019	human stool	N	Y
110065	LNS5952364	Luxembourg	2016	chicken	N	N
110094	LNS9143194	Luxembourg	2021	human stool	N	Y
110975	KF017	Poland	2010	human stool	N	N
111135	Cj-4207	Portugal	2021	human stool	N	Y
111137	Cj-4199	Portugal	2021	human stool	N	Y
111141	Cj-4096	Portugal	2020	human stool	N	N
111149	Cj-4137	Portugal	2020	human stool	N	N
111231	CAM3	Spain	2020	human stool	N	N

[1] unk: an incomplete gene sequence and could not determine if it was functional; Y: present ; N: no ge

[2] unk: an incomplete gene sequence and could not determine if it was functional; Y: present ; N: no ge

[3] wt: wt *cts* genes; D: *ctsD* mutation; DE: ctsDE mutation; E: *ctsE* mutation; F: ctsF mutation
