# Supplementary material for: Genomic Analysis Points to Multiple Genetic Mechanisms for Non-Transformable Campylobacter jejuni ST-50
Source: Microorganisms. 2024 Feb 4;12(2):327. doi: 10.3390/microorganisms12020327 (PMC10893306; doi:10.3390/microorganisms12020327)
Supplement: Supplementary file 1 [file microorganisms-12-00327-s001.zip › Table S3-Parker_et_al2024.pdf]

Table S3. Campylobacter jejuni ST-50 strains from Australia

Isolate name	Year	Bioproject	Accession No.	***dns*** [1]	***dns2*** [2]	***dns3*** [3]	***cts*** [4]
17N3175F1	2017	PRJNA591966	SRR10537747	N	Y	N	E
17N3205F1	2017	PRJNA591966	SRR10537741	N	Y	N	E
17Q3056F1	2017	PRJNA591966	SRR10537529	Y	N	N	wt
17Q3062F1	2017	PRJNA591966	SRR10537522	N	Y	N	wt
17Q3066F1	2017	PRJNA591966	SRR10537518	N	N	N	wt
17Q3071F1	2017	PRJNA591966	SRR10537513	N	N	N	wt
17Q3074F1	2017	PRJNA591966	SRR10537509	N	Y	N	E
17Q3082F1	2017	PRJNA591966	SRR10537500	N	Y	N	wt
17Q3097F1	2017	PRJNA591966	SRR10537477	N	Y	N	E
17Q3102F1	2017	PRJNA591966	SRR10537472	N	Y	N	E
17Q3108F1	2017	PRJNA591966	SRR10537466	N	Y	N	wt
17Q3113F1	2017	PRJNA591966	SRR10537461	N	Y	N	wt
17Q3136F1	2017	PRJNA591966	SRR10538054	Y	N	N	wt
18A3021F1	2018	PRJNA591966	SRR10537601	N	N	N	wt
18A3025F1	2018	PRJNA591966	SRR10537545	N	N	N	wt
18N3050F1	2018	PRJNA591966	SRR10537683	Y	N	N	wt
18Q3012F1	2018	PRJNA591966	SRR10538035	Y	N	N	wt
18Q3020F1	2018	PRJNA591966	SRR10538026	N	Y	N	E
18Q3030F1	2018	PRJNA591966	SRR10538016	N	Y	N	E
18Q3034F1	2018	PRJNA591966	SRR10538012	N	Y	N	E
18V3025F1	2018	PRJNA591966	SRR10537908	Y	N	N	wt
18V3038F1	2018	PRJNA591966	SRR10537893	N	N	N	wt
18V3046F1	2018	PRJNA591966	SRR10537883	N	N	N	wt

[1] Y: present ; N: no gene

[2] Y: present ; N: no gene

[3] Y: present ; N: no gene

[4] wt: wt *cts* genes; E: *ctsE* mutation
