# Supplementary material for: Genomic Analysis Points to Multiple Genetic Mechanisms for Non-Transformable Campylobacter jejuni ST-50
Source: Microorganisms. 2024 Feb 4;12(2):327. doi: 10.3390/microorganisms12020327 (PMC10893306; doi:10.3390/microorganisms12020327)
Supplement: Supplementary file 1 [file microorganisms-12-00327-s001.zip › TableS4-Parker_et_al2024.pdf]

Table S4. *Campylobacter jejuni* ST-8 strains from North America from PubMLST.

PubMLST id	Isolate	country	*dns* [1]	*dns2 or 3* [2]	*cts* [3]
1793	D2643	USA	N	N	wt
1795	D2641	USA	N	N	wt
1796	D2651	USA	N	N	wt
1797	D2763	USA	Y	N	E
1798	D2770	USA	Y	N	E
1799	D2769	USA	Y	N	E
5293	IA3902	USA	N	N	wt
34104	01-1512	Canada	Y	Y	wt
34105	00-0949	Canada	Y	N	wt
57181	BCW_6920	USA	N	N	wt
57182	BCW_6921	USA	N	N	wt
57183	BCW_6922	USA	N	N	wt
57184	BCW_6924	USA	N	N	D
57185	BCW_6925	USA	N	N	wt
57186	BCW_6926	USA	N	N	D
57187	BCW_6927	USA	N	N	wt
57188	BCW_6928	USA	N	N	wt
57190	BCW_6930	USA	N	N	wt
57191	BCW_6931	USA	N	N	wt
57192	BCW_6932	USA	N	N	wt
57193	BCW_6933	USA	N	N	wt
57194	BCW_6934	USA	N	N	wt
60558	W14	Canada	N	N	nd
60559	W15	Canada	N	N	wt
60561	W17	Canada	N	N	nd
60610	RM8893	USA	N	Y	wt
60969	F15M01918	USA	N	Y	E
60970	F15M01909	USA	N	Y	E
60971	F15M01873-C	USA	N	Y	E
60972	F15M01873-B	USA	N	Y	E
60973	F15M01873-A	USA	N	Y	E
60974	F15M01862-D	USA	N	Y	E
60975	F15M01862-C	USA	N	Y	E
60976	F15M01862-B	USA	N	Y	E
60977	F15M01862-A	USA	N	Y	E
60989	F15M00600	USA	N	N	wt
61917	FSIS1702389	USA	N	N	wt
61932	FSIS1605807	USA	N	N	wt
61933	FSIS1710268	USA	N	N	wt
61934	FSIS1710481	USA	N	N	wt
61935	FSIS1702877	USA	N	N	wt
61936	FSIS1501426	USA	N	N	wt
61950	FSIS1504073	USA	N	N	nd

61971	FSIS1501093	USA	N	N	wt
61984	FSIS1606853	USA	N	N	nd
61987	FSIS1607071	USA	N	N	nd
62012	FSIS1608586	USA	N	N	nd
70322	2004392	USA	N	N	wt
70323	2010D-8347	USA	N	N	wt
70324	2014D-0067	USA	N	N	wt
70325	2014D-0068	USA	N	N	wt
70326	2014D-0070	USA	N	N	wt
70327	D4823	USA	N	Y	wt
70328	20060911	USA	N	N	wt
70329	2014D-0187	USA	N	N	wt
70330	2014D-0056	USA	N	N	wt
70331	2014D-0175	USA	N	N	wt
70332	2014D-0184	USA	N	N	wt
70333	D6249	USA	N	N	wt
70334	2014D-0189	USA	N	N	wt
70335	PNUSAC000138	USA	N	N	wt
70336	PNUSAC000139	USA	N	N	wt
70337	PNUSAC000140	USA	N	N	wt
70338	PNUSAC000145	USA	N	N	nd
70339	PNUSAC000144	USA	N	N	wt
70340	PNUSAC000196	USA	N	Y	wt
70341	2014D-0071	USA	N	N	wt
70342	2015D-0043	USA	N	N	wt
78545	SKS6846_26_S18	USA	N	N	wt
78570	SKS6873_53_S57	USA	N	N	wt
79215	PS00239	USA	N	N	nd
81083	PNUSAC009408	USA	N	N	wt
81136	PNUSAC009369	USA	N	N	wt
81201	PNUSAC006157	USA	N	N	wt
81248	PNUSAC005921	USA	N	Y	wt
81369	PNUSAC005782	USA	N	N	nd
81380	PNUSAC005725	USA	N	N	wt
81520	PNUSAC005641	USA	N	N	wt
81547	PNUSAC005589	USA	N	N	wt
81549	PNUSAC005587	USA	N	Y	wt
81559	PNUSAC005513	USA	N	N	wt
81577	PNUSAC005506	USA	N	N	wt
81581	PNUSAC004292	USA	N	Y	wt
81585	PNUSAC005584	USA	N	N	wt
81617	PNUSAC005466	USA	N	Y	wt
81624	PNUSAC005470	USA	Y	Y	wt
81628	PNUSAC005671	USA	N	N	wt
81682	PNUSAC005432	USA	N	N	wt
81690	PNUSAC005410	USA	N	N	wt
81725	PNUSAC005414	USA	N	N	nd

81742	PNUSAC005336	USA	N	N	wt
81785	PNUSAC005498	USA	N	Y	wt
81792	PNUSAC005480	USA	N	N	wt
81795	PNUSAC005477	USA	N	N	wt
81842	PNUSAC005394	USA	N	N	wt
81907	PNUSAC005365	USA	N	N	wt
81917	PNUSAC005320	USA	N	N	wt
81951	PNUSAC005299	USA	N	N	wt
82012	PNUSAC005110	USA	N	N	wt
82080	PNUSAC005258	USA	N	N	wt
82085	PNUSAC005201	USA	N	N	wt
82131	PNUSAC005075	USA	N	N	wt
82135	PNUSAC005062	USA	N	N	wt
82250	PNUSAC004961	USA	N	N	F
82251	PNUSAC004725	USA	N	N	wt
82308	PNUSAC000080	USA	N	N	wt
82312	PNUSAC000079	USA	N	N	wt
82316	PNUSAC004917	USA	N	N	wt
82345	PNUSAC004904	USA	N	N	wt
82371	PNUSAC004839	USA	N	Y	nd
82372	PNUSAC004837	USA	N	N	wt
82389	PNUSAC004851	USA	N	Y	wt
82395	PNUSAC004846	USA	N	N	nd
82425	PNUSAC004815	USA	N	Y	wt
82432	PNUSAC004800	USA	N	Y	wt
82440	PNUSAC004803	USA	N	Y	wt
82451	PNUSAC004763	USA	N	Y	wt
82454	PNUSAC004855	USA	N	N	wt
82465	PNUSAC004796	USA	N	Y	wt
82477	PNUSAC004797	USA	N	Y	wt
82483	PNUSAC004670	USA	N	N	wt
82494	PNUSAC004736	USA	N	Y	wt
82608	D4808	USA	N	N	wt
82636	TESTWGCAMPY0003969	USA	N	N	wt
82644	TESTWGCAMPY0003785	USA	N	N	wt
82659	PNUSAC000649	USA	N	N	wt
82677	PNUSAC000149	USA	N	N	wt
82741	PNUSAC002005	USA	N	N	wt
82744	PNUSAC002009	USA	N	N	wt
82752	PNUSAC001749	USA	N	N	wt
82762	PNUSAC001126	USA	N	N	E
82766	PNUSAC001125	USA	N	N	wt
82799	PNUSAC001818	USA	N	Y	wt
82816	PNUSAC009233	USA	N	N	nd
82837	PNUSAC001674	USA	N	N	wt
82903	PNUSAC001666	USA	N	Y	wt
82906	PNUSAC001731	USA	N	N	wt

82916	PNUSAC001543	USA	N	N	wt
82917	PNUSAC001498	Unknown	N	N	wt
82961	PNUSAC000916	USA	N	N	nd
82981	PNUSAC000489	USA	N	N	wt
82982	PNUSAC000602	USA	N	N	wt
82986	PNUSAC000536	USA	N	N	wt
83027	PNUSAC000914	USA	N	N	nd
83028	PNUSAC000920	USA	N	N	wt
83033	TESTWGCAMPY0003839	USA	N	N	wt
83037	TESTWGCAMPY0004093	USA	N	N	wt
83140	PNUSAC008839	USA	N	Y	nd
83156	PNUSAC002972	USA	N	Y	wt
83187	PNUSAC003696	USA	N	N	wt
83205	PNUSAC001964	USA	N	N	wt
83266	PNUSAC002892	USA	N	N	wt
83472	PNUSAC001541	USA	N	N	wt
83476	PNUSAC002631	USA	N	N	wt
83578	PNUSAC001961	USA	N	N	wt
83582	PNUSAC004129	USA	N	N	nd
83593	PNUSAC002901	USA	N	N	wt
83631	PNUSAC003634	USA	N	N	wt
83700	PNUSAC003478	USA	N	N	wt
83703	PNUSAC003480	USA	N	N	wt
83801	PNUSAC001647	USA	N	N	wt
83817	PNUSAC001732	USA	N	N	wt
83821	PNUSAC000911	USA	N	N	wt
83877	PNUSAC001733	USA	N	N	wt
83903	PNUSAC004383	USA	N	N	wt
83913	PNUSAC004508	USA	N	Y	wt
83915	PNUSAC004359	USA	N	N	wt
83927	PNUSAC003968	USA	N	Y	nd
84084	PNUSAC000745	USA	N	Y	wt
84109	PNUSAC003390	USA	N	N	E
84167	PNUSAC000900	Unknown	N	N	wt
84249	PNUSAC003139	USA	N	N	wt
84343	PNUSAC000657	Unknown	N	N	wt
84363	PNUSAC000624	USA	N	Y	wt
84365	PNUSAC000625	USA	N	Y	wt
84377	PNUSAC000411	USA	N	Y	F
84497	TESTWGCAMPY0003838	USA	N	N	wt
84502	TESTWGCAMPY0003783	USA	N	N	wt
84531	PNUSAC000256	USA	N	N	wt
84544	TESTWGCAMPY10002532	USA	N	N	wt
84549	TESTWGCAMPY0002162	USA	N	N	wt
84550	PNUSAC000171	USA	N	N	wt
84575	TESTWGCAMPY0001792	USA	N	N	wt
84620	TESTWGCAMPY10002535	USA	N	N	wt

84636	PNUSAC000150	USA	N	N	wt
84642	TESTWGCAMPY0001711	USA	N	N	wt
84644	TESTWGCAMPY0001708	USA	N	N	wt
84674	TESTWGCAMPY0002208	USA	N	Y	wt
84675	TESTWGCAMPY0002174	USA	Y	N	wt
84677	PNUSAC000142	USA	N	N	wt
84702	PNUSAC000060	USA	N	N	wt
84747	PNUSAC007759	USA	N	Y	wt
84774	PNUSAC001412	USA	N	N	wt
84808	PNUSAC008854	USA	N	N	wt
84872	PNUSAC001706	USA	N	N	wt
84876	PNUSAC001734	USA	N	N	wt
84899	2016D-0216	USA	N	N	nd
84942	PNUSAC001052	USA	N	N	wt
84984	PNUSAC000934	Unknown	N	N	nd
84995	PNUSAC000924	USA	N	Y	wt
84996	PNUSAC000925	USA	N	Y	wt
85010	PNUSAC000917	USA	N	N	nd
85011	PNUSAC000915	USA	N	N	wt
85013	PNUSAC000912	USA	N	N	nd
85044	PNUSAC000361	USA	N	Y	wt
85045	PNUSAC000366	USA	N	Y	wt
85049	PNUSAC000369	USA	N	Y	wt
85081	PNUSAC000665	USA	N	N	wt
85213	PNUSAC000534	Unknown	N	N	wt
85232	PNUSAC000446	USA	N	N	wt
85269	PNUSAC000419	USA	N	Y	wt
85307	TESTWGCAMPY1000266[illegible]	USA	N	N	wt
85324	TESTWGCAMPY0002250	USA	N	N	wt
85326	TESTWGCAMPY1000252[illegible]	USA	N	N	wt
85367	TESTWGCAMPY0002164	USA	N	N	wt
85382	TESTWGCAMPY0001541	USA	N	N	wt
85404	PNUSAC000042	USA	N	N	wt
85407	PNUSAC000012	USA	N	Y	wt
85410	TESTWGCAMPY0002404	USA	N	N	wt
85448	PNUSAC008992	USA	N	N	nd
85534	PNUSAC000147	USA	N	N	wt
85575	PNUSAC004492	USA	N	N	nd
85587	PNUSAC004459	USA	N	N	wt
85595	PNUSAC004293	USA	N	N	nd
85599	PNUSAC004205	USA	N	Y	E
85634	PNUSAC004405	USA	N	N	nd
85643	PNUSAC004099	USA	Y	Y	wt
85648	PNUSAC003934	USA	N	Y	wt
85666	PNUSAC003933	USA	N	N	wt
85689	PNUSAC003970	USA	N	N	nd
85709	PNUSAC004493	USA	N	N	nd

85810	PNUSAC000792	USA	N	N	wt
85833	PNUSAC004015	USA	N	N	nd
85841	PNUSAC003469	USA	N	Y	wt
85914	PNUSAC003856	USA	N	Y	nd
85928	PNUSAC004209	USA	N	N	nd
85998	PNUSAC004248	USA	N	N	wt
86053	PNUSAC003925	USA	N	N	wt
86110	PNUSAC003424	USA	N	N	wt
86114	PNUSAC003419	USA	N	N	wt
86200	PNUSAC004043	USA	N	Y	wt
86264	PNUSAC000538	USA	N	Y	wt
86287	PNUSAC003705	USA	N	N	wt
86294	PNUSAC003591	USA	Y	N	wt
86297	PNUSAC000788	USA	N	N	nd
86309	PNUSAC003567	USA	N	N	nd
86328	PNUSAC000575	USA	N	N	wt
86329	PNUSAC003541	USA	N	N	wt
86397	PNUSAC002715	USA	N	Y	wt
86501	PNUSAC002482	USA	N	N	wt
86510	PNUSAC001912	USA	N	N	wt
86567	PNUSAC002378	USA	N	Y	wt
86610	PNUSAC001917	USA	N	N	wt
86678	PNUSAC002699	USA	N	N	wt
86705	PNUSAC002773	USA	Y	N	wt
86733	PNUSAC001542	USA	N	N	wt
86843	PNUSAC003739	USA	N	N	wt
86858	PNUSAC003719	USA	N	Y	wt
87018	PNUSAC003422	USA	N	N	wt
87081	PNUSAC000584	USA	N	N	wt
87088	PNUSAC000855	USA	N	N	wt
87094	PNUSAC000401	USA	N	Y	F
87117	PNUSAC003211	USA	N	N	wt
87123	PNUSAC003164	USA	N	N	wt
87125	PNUSAC003161	USA	N	Y	wt
87224	PNUSAC002935	USA	N	N	wt
87305	PNUSAC002688	USA	N	N	wt
87317	PNUSAC002635	USA	N	N	wt
87322	PNUSAC002617	USA	N	N	wt
87430	PNUSAC002476	USA	N	N	wt
87529	PNUSAC002226	USA	N	N	wt
87593	PNUSAC001911	USA	N	N	wt
87597	PNUSAC001952	USA	N	N	wt
87654	PNUSAC001713	USA	N	N	nd
87660	PNUSAC001554	USA	N	Y	F
87675	PNUSAC001497	Unknown	N	N	wt
87695	PNUSAC001544	USA	N	N	wt
87749	PNUSAC001065	USA	N	N	wt

87764	PNUSAC000957	USA	N	N	nd
87766	PNUSAC000953	USA	N	N	wt
87773	PNUSAC000918	USA	N	N	nd
87774	PNUSAC000913	USA	N	N	nd
87821	PNUSAC000650	USA	N	N	wt
87829	PNUSAC000634	USA	N	N	wt
87837	PNUSAC000628	USA	N	Y	wt
87861	PNUSAC000553	USA	N	N	wt
87863	PNUSAC000405	USA	N	Y	F
87886	PNUSAC000418	USA	N	Y	wt
87903	PNUSAC000629	USA	N	N	wt
87963	TESTWGCAMPY0003840	USA	N	N	wt
87985	PNUSAC000225	USA	N	N	nd
88009	PNUSAC000143	USA	N	N	wt
88011	PNUSAC000141	USA	N	N	wt
88013	PNUSAC000137	USA	N	N	wt
88031	TESTWGCAMPY0001699	USA	N	N	wt
88041	TESTWGCAMPY0002418	USA	N	N	wt
88060	TESTWGCAMPY0002163	USA	N	N	wt
88068	TESTWGCAMPY0001766	USA	Y	N	E
88075	TESTWGCAMPY0001767	USA	Y	N	E
88112	TESTWGCAMPY0001709	USA	N	N	wt
88187	PNUSAC008873	USA	N	Y	wt
88207	PNUSAC008819	USA	N	N	wt
88238	PNUSAC008830	USA	N	N	wt
88241	PNUSAC008825	USA	N	N	wt
88295	PNUSAC008331	USA	N	N	nd
88299	PNUSAC008182	USA	N	N	wt
88301	PNUSAC008333	USA	N	N	nd
88307	PNUSAC008181	USA	N	N	nd
88324	PNUSAC008329	USA	N	Y	nd
88325	PNUSAC008312	USA	N	N	wt
88330	PNUSAC008332	USA	N	N	nd
88337	PNUSAC008694	USA	N	N	nd
88348	PNUSAC008311	USA	N	N	wt
88357	PNUSAC008307	USA	N	N	nd
88360	PNUSAC008310	USA	N	N	wt
88408	PNUSAC008306	USA	N	N	nd
88414	PNUSAC008308	USA	N	N	nd
88442	PNUSAC007696	USA	N	N	nd
88445	PNUSAC007697	USA	N	N	wt
88464	PNUSAC007411	USA	N	N	wt
88479	PNUSAC007355	USA	Y	Y	wt
88493	PNUSAC007328	USA	N	N	wt
88528	PNUSAC007258	USA	N	N	wt
88663	PNUSAC006851	USA	N	Y	wt
88684	PNUSAC006860	USA	N	N	nd

88715	PNUSAC008516	USA	N	Y	wt
88808	PNUSAC007850	USA	N	N	wt
88813	PNUSAC007704	USA	N	N	nd
88820	PNUSAC007883	USA	N	N	wt
88826	20110057	USA	N	N	wt
88843	PNUSAC007884	USA	N	N	wt
89122	PNUSAC008154	USA	N	N	wt
89184	PNUSAC007522	USA	N	N	nd
89345	PNUSAC007476	USA	N	N	wt
89388	PNUSAC007882	USA	N	N	wt
89443	PNUSAC007390	USA	N	N	F
89498	PNUSAC007706	USA	N	N	nd
89518	PNUSAC007678	USA	N	N	wt
89551	PNUSAC007351	USA	Y	Y	wt
89553	PNUSAC007644	USA	N	N	wt
89584	PNUSAC007354	USA	Y	Y	wt
89799	PNUSAC007039	USA	N	N	nd
89896	PNUSAC006768	USA	N	N	wt
89899	PNUSAC004730	USA	N	Y	wt
90021	PNUSAC006370	USA	N	Y	wt
90059	PNUSAC006443	USA	N	Y	wt
90177	PNUSAC006172	USA	Y	Y	wt
90192	PNUSAC006057	USA	N	N	wt
90261	PNUSAC006237	USA	N	N	wt
90336	PNUSAC006896	USA	N	N	wt
90350	PNUSAC006171	USA	Y	N	wt
90353	PNUSAC006254	USA	N	N	nd
90473	PNUSAC006697	USA	N	Y	nd
90525	PNUSAC006604	USA	N	Y	wt
90552	PNUSAC006639	USA	N	N	wt
90878	FSIS11921295	USA	N	N	wt
91189	FSIS31800973	USA	N	N	wt
91197	FSIS11811797	USA	N	Y	wt
91694	FSIS31800677	USA	N	N	nd
91945	FSIS11810346	USA	N	Y	wt
92266	FSIS1710818	USA	N	N	wt
92834	FSIS1701579	USA	N	N	wt
93139	FSIS1608397	USA	N	N	wt
93586	FSIS1700402	USA	N	N	wt
93639	FSIS1700904	USA	N	N	wt
93921	FSIS1700405	USA	N	N	wt
93971	FSIS1607781	USA	N	N	wt
96141	CVM N17C393	USA	Y	Y	wt
96337	CVM N17C392	USA	N	Y	wt
96732	CVM N17C120	USA	N	N	wt
97318	CVM N16C157	USA	N	N	wt
98036	CVM N18C414	USA	N	Y	nd

98246	CVM N18C145	USA	N	Y	wt
98454	FSIS11921469	USA	N	N	wt
98479	FSIS11921502	USA	N	N	wt
98507	FSIS11921534	USA	N	N	nd
98509	FSIS1606913	USA	N	Y	wt
98525	FSIS11921271	USA	N	N	nd
98533	FSIS11921269	USA	N	N	wt
98534	FSIS11921261	USA	N	Y	F
98535	FSIS11921264	USA	Y	N	wt
98559	FSIS11920894	USA	N	N	wt
98581	FSIS11921065	USA	Y	Y	nd
98583	FSIS11921060	USA	Y	Y	wt
98591	FSIS11921105	USA	Y	N	wt
98597	FSIS11921098	USA	N	N	wt
98601	FSIS11921061	USA	Y	Y	nd
98627	FSIS11921097	USA	N	N	wt
98676	FSIS11920886	USA	N	N	wt
98677	FSIS11920891	USA	N	N	wt
98703	FSIS11813560	USA	N	N	nd
98727	FSIS11813809	USA	N	N	wt
98763	FSIS11813361	USA	N	N	wt
98775	FSIS11813368	USA	N	Y	nd
98780	FSIS11813379	USA	N	Y	wt
98785	FSIS11813395	USA	N	Y	wt
98792	FSIS11813179	USA	N	N	nd
98795	FSIS11813172	USA	N	Y	wt
98851	FSIS11810133	USA	N	Y	wt
98859	FSIS11810986	USA	N	Y	wt
98862	FSIS11810132	USA	N	N	wt
98863	FSIS11811562	USA	N	N	wt
98874	FSIS11705603	USA	Y	N	nd
98879	FSIS11812625	USA	N	N	nd
98881	FSIS11812620	USA	N	N	wt
98907	FSIS11812596	USA	N	N	wt
98911	FSIS11812588	USA	N	N	wt
98990	FSIS11812058	USA	N	N	E
98996	FSIS11812057	USA	N	N	E
99012	FSIS11812059	USA	N	Y	nd
99065	FSIS11811842	USA	N	N	nd
99086	FSIS11811301	USA	N	N	nd
99098	FSIS11811569	USA	N	N	nd
99111	FSIS11810765	USA	Y	N	nd
99120	FSIS11811275	USA	Y	Y	wt
99127	FSIS11811010	USA	N	N	F
99134	FSIS11810995	USA	N	N	wt
99136	FSIS11811013	USA	N	Y	wt
99138	FSIS11811000	USA	N	Y	nd

99157	FSIS11811283	USA	N	N	wt
99183	FSIS11810786	USA	N	N	nd
99227	FSIS11810415	USA	N	N	wt
99231	FSIS11810778	USA	N	N	nd
99238	FSIS11810403	USA	N	N	nd
99249	FSIS11810385	USA	N	N	nd
99260	FSIS11810412	USA	Y	Y	wt
99273	FSIS11809049	USA	N	N	wt
99343	FSIS11810115	USA	N	N	wt
99352	FSIS11810143	USA	N	N	wt
99359	FSIS11810150	USA	N	N	wt
99367	FSIS11810138	USA	N	N	nd
99368	FSIS11810147	USA	N	N	wt
99370	FSIS11810149	USA	N	N	wt
99382	FSIS1501987	USA	N	N	wt
99386	FSIS1607148	USA	N	Y	nd
99393	FSIS1607230	USA	N	Y	nd
99403	FSIS1607227	USA	N	N	nd
99408	FSIS1607092	USA	N	N	wt
99415	FSIS1607152	USA	N	Y	nd
99417	FSIS1607139	USA	N	N	wt
99425	FSIS1607095	USA	N	Y	wt
99435	FSIS1607089	USA	N	Y	wt
99444	FSIS1607134	USA	N	N	wt
99484	FSIS1606948	USA	N	N	wt
99500	FSIS1606949	USA	N	N	wt
99507	FSIS1606843	USA	N	N	wt
99512	FSIS1606906	USA	N	N	wt
99519	FSIS1606914	USA	N	N	wt
99545	FSIS1606817	USA	N	N	wt
99546	FSIS1606844	USA	N	Y	F
99547	FSIS1606856	USA	N	Y	wt
99561	FSIS1606805	USA	N	N	wt
99582	FSIS1606794	USA	N	Y	wt
99604	FSIS1606460	USA	N	Y	wt
99606	FSIS1606734	USA	N	N	wt
99615	FSIS1606727	USA	N	Y	wt
99618	FSIS1606720	USA	Y	Y	nd
99624	FSIS1606662	USA	N	N	wt
99696	FSIS1606588	USA	N	N	wt
99721	FSIS1606356	USA	N	N	wt
99722	FSIS1606358	USA	N	N	wt
99739	FSIS1606386	USA	N	N	nd
99746	FSIS1606381	USA	N	Y	wt
99749	FSIS1606319	USA	N	N	wt
99775	FSIS1606218	USA	N	N	wt
99824	FSIS1606233	USA	N	N	wt

99826	FSIS1606227	USA	N	N	wt
99832	FSIS1605864	USA	N	N	wt
99833	FSIS1605855	USA	Y	Y	wt
99852	FSIS1606107	USA	N	Y	nd
99878	FSIS1606100	USA	N	N	E
99899	FSIS1605988	USA	N	N	wt
99908	FSIS1605876	USA	N	Y	wt
99911	FSIS1605847	USA	N	N	wt
99914	FSIS1605944	USA	N	N	wt
99919	FSIS1606010	USA	N	Y	wt
99929	FSIS1605935	USA	N	N	wt
99939	FSIS1605880	USA	N	Y	wt
99959	FSIS1605862	USA	N	N	wt
99972	FSIS1605996	USA	N	Y	F
99976	FSIS1605924	USA	N	Y	wt
99978	FSIS1605922	USA	N	N	wt
99986	FSIS1605840	USA	N	Y	wt
99995	FSIS1605727	USA	N	N	nd
100012	FSIS1605777	USA	Y	N	nd
100039	FSIS1605793	USA	N	N	wt
100067	FSIS1605637	USA	N	N	wt
100096	FSIS1605593	USA	N	N	wt
100100	FSIS1605595	USA	N	N	wt
100108	FSIS1700163	USA	N	Y	wt
100124	FSIS1700137	USA	Y	Y	F
100126	FSIS1700118	USA	N	Y	wt
100138	FSIS1711102	USA	N	N	wt
100149	FSIS11920697	USA	N	Y	nd
100155	FSIS11920705	USA	N	N	wt
100170	FSIS11920687	USA	N	N	wt
100217	FSIS1701645	USA	N	N	wt
100223	FSIS1701646	USA	N	N	nd
100227	FSIS1701654	USA	N	N	wt
100228	FSIS1701641	USA	N	N	wt
100234	FSIS1701507	USA	Y	Y	F
100241	FSIS1701515	USA	N	N	wt
100244	FSIS1701506	USA	Y	Y	F
100252	FSIS1701503	USA	N	N	nd
100263	FSIS11920440	USA	N	N	nd
100279	FSIS11920454	USA	N	N	nd
100282	FSIS11920458	USA	N	Y	nd
100312	FSIS11920455	USA	Y	N	wt
100319	FSIS1700327	USA	N	N	wt
100335	FSIS1700305	USA	N	N	wt
100354	FSIS1710455	USA	N	N	wt
100376	FSIS1608467	USA	N	N	nd
100378	FSIS1608593	USA	N	N	wt

100383	FSIS1607944	USA	N	N	wt
100386	FSIS1607954	USA	N	N	wt
100403	FSIS1609478	USA	Y	Y	nd
100448	FSIS11918631	USA	N	N	wt
100468	FSIS11920219	USA	Y	Y	F
100500	FSIS1702074	USA	N	Y	wt
100542	FSIS1711096	USA	N	N	wt
100544	FSIS1702734	USA	N	N	wt
100551	FSIS11920007	USA	N	N	wt
100564	FSIS11808180	USA	Y	Y	nd
100606	FSIS1710910	USA	N	N	nd
100632	FSIS11919331	USA	N	N	wt
100635	FSIS1702556	USA	Y	N	wt
100639	FSIS11918843	USA	N	N	nd
100649	FSIS1700964	USA	N	Y	nd
100665	FSIS11919317	USA	Y	Y	wt
100735	FSIS11807799	USA	Y	N	wt
100746	FSIS11705793	USA	N	N	wt
100754	FSIS11808183	USA	N	N	wt
100762	FSIS11807986	USA	Y	N	wt
100763	FSIS11809038	USA	N	N	nd
100803	FSIS1609289	USA	N	Y	wt
100804	FSIS1702399	USA	N	Y	wt
100814	FSIS1700131	USA	N	N	nd
100816	FSIS1711112	USA	N	Y	wt
100817	FSIS11703895	USA	N	N	nd
100824	FSIS1700976	USA	N	N	wt
100837	FSIS1702549	USA	N	N	wt
100845	FSIS11705227	USA	N	Y	wt
100848	FSIS11705224	USA	N	N	wt
100858	FSIS11706909	USA	N	N	wt
100860	FSIS11807088	USA	N	N	wt
100872	FSIS11705199	USA	N	Y	nd
100881	FSIS11807177	USA	N	N	nd
100896	FSIS11807833	USA	N	N	F
100946	FSIS11704504	USA	N	N	wt
100953	FSIS11705763	USA	N	N	wt
100992	FSIS11705921	USA	N	Y	nd
101005	FSIS11807517	USA	N	N	wt
101008	FSIS11809013	USA	N	Y	F
101009	FSIS11706468	USA	N	N	wt
101037	FSIS11706215	USA	N	N	wt
101047	FSIS11807040	USA	N	N	wt
101076	FSIS1710622	USA	N	N	wt
101117	FSIS1608609	USA	Y	Y	1
101146	FSIS11705395	USA	N	Y	wt
101172	FSIS11809823	USA	N	N	wt

101175	FSIS11808751	USA	Y	Y	wt
101189	FSIS11807502	USA	Y	Y	F
101195	FSIS11809337	USA	N	Y	wt
101196	FSIS11807843	USA	N	Y	nd
101208	FSIS11706255	USA	N	N	wt
101242	FSIS11706261	USA	N	N	wt
101249	FSIS11704327	USA	N	N	wt
101278	FSIS11704011	USA	N	N	E
101281	FSIS1710625	USA	N	N	wt
101302	FSIS1608618	USA	N	Y	wt
101341	FSIS1710047	USA	N	Y	wt
101346	FSIS1609788	USA	N	Y	E
101349	FSIS1609775	USA	Y	Y	wt
101360	FSIS1608182	USA	N	N	wt
101366	FSIS1609662	USA	N	N	wt
101387	FSIS1607377	USA	Y	Y	F
101403	FSIS1701191	USA	N	N	wt
101423	FSIS1703611	USA	N	Y	nd
101478	FSIS1505093	USA	N	N	wt
101486	FSIS1504900	USA	N	Y	nd
101499	FSIS1501966	USA	N	Y	nd
101507	FSIS1503662	USA	N	N	wt
101511	FSIS1502068	USA	N	N	wt
101520	FSIS1502092	USA	Y	Y	wt
101534	FSIS1501962	USA	N	N	wt
101542	FSIS1502924	USA	N	N	wt
101551	FSIS1504648	USA	Y	Y	wt
101557	FSIS1501338	USA	N	Y	wt
101564	FSIS1501327	USA	N	N	wt
101567	FSIS1501333	USA	Y	N	wt
101573	FSIS1501798	USA	N	N	wt
101590	FSIS11919802	USA	Y	N	wt
101614	FSIS11919775	USA	N	N	wt
101635	FSIS11918839	USA	N	N	wt
101642	FSIS11918822	USA	N	Y	wt
101697	FSIS1609784	USA	Y	Y	wt
101715	FSIS11920241	USA	N	Y	wt
101742	FSIS11920041	USA	N	Y	nd
101800	FSIS1609136	USA	N	N	wt
101803	FSIS1710153	USA	N	N	wt
101816	FSIS1710601	USA	N	N	wt
101850	FSIS1609278	USA	N	Y	E
101853	FSIS1609277	USA	N	N	nd
101876	FSIS1608472	USA	N	Y	wt
101877	FSIS1608607	USA	N	N	wt
101894	FSIS1608461	USA	N	N	wt
101899	FSIS1608178	USA	N	N	wt

101942	FSIS1607435	USA	N	N	wt
101962	FSIS1607365	USA	N	N	wt
101967	FSIS1607315	USA	N	Y	wt
101969	FSIS1607251	USA	N	N	wt
101976	FSIS1504912	USA	N	Y	wt
102053	FSIS1501976	USA	N	Y	wt
102079	FSIS1504642	USA	N	Y	wt
102084	FSIS1501974	USA	N	N	wt
102086	FSIS1501983	USA	N	N	wt
102087	FSIS1501343	USA	N	N	wt
102090	FSIS11919770	USA	N	Y	wt
102116	FSIS11919547	USA	N	Y	wt
102120	FSIS11919558	USA	N	N	nd
102144	FSIS11918596	USA	N	N	wt
102189	FSIS11918830	USA	N	N	F
102248	FSIS11809507	USA	N	Y	wt
102252	FSIS11809636	USA	N	N	nd
102253	FSIS11809036	USA	N	Y	wt
102256	FSIS11807667	USA	N	N	wt
102258	FSIS11809508	USA	N	Y	wt
102260	FSIS11809689	USA	N	Y	wt
102291	FSIS11808209	USA	N	Y	nd
102313	FSIS11809688	USA	N	N	wt
102314	FSIS11808608	USA	N	Y	nd
102319	FSIS11809833	USA	N	N	wt
102338	FSIS11706599	USA	N	Y	wt
102341	FSIS11809532	USA	N	N	nd
102344	FSIS11807062	USA	N	N	wt
102367	FSIS11809324	USA	N	N	wt
102380	FSIS11809322	USA	N	N	wt
102383	FSIS11808452	USA	Y	Y	wt
102386	FSIS11808456	USA	N	Y	wt
102440	FSIS11704505	USA	N	N	nd
102442	FSIS11704487	USA	N	Y	nd
102448	FSIS11704715	USA	N	Y	nd
102458	FSIS11809830	USA	N	N	wt
102464	FSIS11809346	USA	N	Y	wt
102469	FSIS11809156	USA	N	Y	wt
102471	FSIS11809042	USA	N	Y	wt
102504	FSIS11809505	USA	N	N	wt
102509	FSIS11809516	USA	N	N	wt
102542	FSIS11705914	USA	N	N	wt
102548	FSIS11706866	USA	N	N	wt
102556	FSIS11809539	USA	N	Y	wt
102563	FSIS11808197	USA	N	Y	nd
102584	FSIS11705920	USA	N	N	wt
102664	FSIS11705019	USA	N	N	wt

102667	FSIS11809187	USA	N	N	wt
102668	FSIS11809185	USA	N	N	wt
102672	FSIS11808427	USA	Y	Y	wt
102677	FSIS11807317	USA	N	N	wt
102720	FSIS11808603	USA	N	N	wt
102743	FSIS11809024	USA	N	Y	nd
102756	FSIS11706917	USA	N	N	wt
102758	FSIS11706426	USA	N	N	nd
102771	FSIS11807176	USA	N	N	nd
102793	FSIS11807093	USA	N	N	nd
102796	FSIS11706911	USA	N	Y	nd
102798	FSIS11807200	USA	N	Y	wt
102800	FSIS11704535	USA	N	Y	wt
102822	FSIS11706729	USA	N	Y	wt
102838	FSIS11809847	USA	N	N	nd
102881	FSIS11705404	USA	Y	Y	nd
102888	FSIS11809008	USA	N	Y	nd
102893	FSIS11808622	USA	N	Y	wt
102933	FSIS11706882	USA	N	Y	nd
102956	FSIS11807996	USA	N	N	wt
102963	FSIS11808215	USA	N	Y	nd
102987	FSIS11706440	USA	N	N	wt
103010	FSIS11706427	USA	N	N	wt
103012	FSIS11706900	USA	N	N	nd
103023	FSIS11705802	USA	N	Y	wt
103038	FSIS11704200	USA	N	N	wt
103041	FSIS11704546	USA	N	N	nd
103050	FSIS11704172	USA	N	N	wt
103063	FSIS11705419	USA	N	N	nd
103113	FSIS11703902	USA	N	N	nd
103157	FSIS1702888	USA	N	N	wt
103189	FSIS1700676	USA	N	Y	wt
103191	FSIS1702095	USA	N	N	wt
103220	FSIS1701488	USA	N	N	wt
103221	FSIS1700638	USA	N	N	wt
103228	FSIS1702260	USA	N	N	wt
103244	FSIS1700154	USA	N	N	wt
103245	FSIS1700124	USA	Y	Y	F
103275	FSIS1703441	USA	N	N	nd
103278	FSIS1702875	USA	Y	N	wt
103285	FSIS1710468	USA	N	Y	wt
103287	FSIS1700990	USA	N	N	wt
103325	FSIS1702560	USA	N	N	wt
103331	FSIS1710458	USA	N	N	wt
103356	FSIS11705017	USA	N	N	nd
103357	FSIS11807846	USA	N	Y	nd
103399	FSIS11807513	USA	N	Y	wt

103473	FSIS11706867	USA	N	N	nd
103475	FSIS11706740	USA	N	Y	wt
103477	FSIS11706873	USA	N	N	wt
103480	FSIS11706764	USA	N	N	wt
103490	FSIS11706880	USA	Y	Y	F
103502	FSIS11706929	USA	N	N	nd
103505	FSIS11807184	USA	N	Y	nd
103516	FSIS11706073	USA	N	N	nd
103517	FSIS11706879	USA	N	N	nd
103519	FSIS11807039	USA	N	N	wt
103539	FSIS11706891	USA	N	N	wt
103540	FSIS11807049	USA	N	N	wt
103556	FSIS11706601	USA	N	Y	wt
103596	FSIS11706259	USA	N	N	wt
103636	FSIS11705423	USA	N	Y	nd
103638	FSIS11705804	USA	N	N	wt
103648	FSIS11706400	USA	N	N	nd
103679	FSIS11704748	USA	N	N	nd
103692	FSIS11704713	USA	N	Y	nd
103703	FSIS11704743	USA	N	N	wt
103745	FSIS11704514	USA	N	Y	wt
103789	FSIS11703918	USA	N	N	wt
103808	FSIS11703899	USA	N	N	E
103817	FSIS1703794	USA	N	N	nd
103820	FSIS1703628	USA	N	N	wt
103856	FSIS1703251	USA	N	Y	wt
103902	FSIS1702873	USA	N	N	F
103903	FSIS1702857	USA	N	N	nd
103910	FSIS1702713	USA	N	N	nd
103998	FSIS1701494	USA	N	Y	F
104006	FSIS1700981	USA	N	N	wt
104013	FSIS1700968	USA	N	N	wt
104039	FSIS1700485	USA	N	N	wt
104053	FSIS1700670	USA	N	Y	wt
104061	FSIS1700826	USA	N	Y	wt
104084	FSIS1700486	USA	N	Y	wt
104096	FSIS1711071	USA	N	N	wt
104100	FSIS1711070	USA	N	Y	wt
104115	FSIS1609024	USA	N	N	nd
104117	FSIS1710457	USA	N	N	wt
104140	FSIS1710059	USA	N	Y	wt
104152	FSIS1609657	USA	N	N	wt
104154	FSIS1609023	USA	N	N	wt
104158	FSIS1609480	USA	N	N	nd
104198	FSIS1607843	USA	N	Y	F
104209	FSIS1607734	USA	N	N	wt
104225	FSIS1607462	USA	N	N	D

104240	FSIS1607378	USA	N	N	wt
104279	FSIS1505125	USA	N	N	wt
104336	FSIS1502920	USA	N	N	wt
104358	FSIS1504655	USA	N	Y	wt
104378	FSIS11816281	USA	N	N	nd
104389	FSIS11918472	USA	N	Y	wt
104400	FSIS11918477	USA	N	N	wt
104463	FSIS11816480	USA	N	N	wt
104473	FSIS11816453	USA	N	Y	wt
104475	FSIS11816677	USA	N	N	wt
104490	FSIS11918171	USA	N	N	nd
104500	FSIS11918193	USA	N	N	nd
104551	FSIS11918168	USA	N	Y	F
104561	FSIS11815690	USA	N	N	wt
104571	FSIS11918235	USA	N	N	wt
104586	FSIS11918163	USA	N	Y	nd
104601	FSIS11918236	USA	N	N	wt
104620	FSIS11816035	USA	N	N	wt
104626	FSIS11816037	USA	N	N	wt
104638	FSIS11815861	USA	Y	N	nd
104673	FSIS11917957	USA	N	N	wt
104697	FSIS11815245	USA	N	Y	wt
104700	FSIS11815256	USA	N	N	wt
104704	FSIS11815264	USA	Y	Y	wt
104726	FSIS11917653	USA	N	N	wt
104729	FSIS11917663	USA	N	N	F
104743	FSIS11917915	USA	N	N	nd
104766	FSIS11917646	USA	N	Y	F
104787	FSIS11917923	USA	N	N	wt
104793	FSIS11917491	USA	N	N	wt
104839	FSIS11917199	USA	Y	Y	wt
104847	FSIS11917145	USA	N	N	wt
104848	FSIS11917146	USA	N	N	wt
104859	FSIS11917150	USA	N	Y	wt
104862	FSIS11917159	USA	N	N	wt
104863	FSIS11917180	USA	N	Y	wt
104865	FSIS11917148	USA	N	N	wt
104876	FSIS11917194	USA	N	Y	wt
104883	FSIS11917196	USA	Y	N	wt
104913	FSIS11816867	USA	N	Y	nd
104918	FSIS11816866	USA	N	Y	nd
104946	FSIS11816880	USA	N	Y	wt
104954	FSIS11816489	USA	N	N	nd
104988	FSIS11816437	USA	N	Y	wt
105050	FSIS11815672	USA	N	N	wt
105057	FSIS11815698	USA	N	Y	wt
105071	FSIS11816047	USA	N	Y	wt

105080	FSIS11815881	USA	N	N	nd
105113	FSIS11815467	USA	N	N	wt
105118	FSIS11815455	USA	N	N	wt
105164	FSIS11814758	USA	N	Y	wt
105186	FSIS11814594	USA	N	N	wt
105225	FSIS11814012	USA	Y	N	wt
105236	FSIS11814262	USA	N	N	wt
105257	FSIS11814272	USA	N	Y	wt
105266	FSIS11814247	USA	N	N	E
105309	FSIS11815661	USA	N	N	wt
105346	FSIS11815481	USA	N	Y	wt
105364	FSIS11815000	USA	N	Y	nd
105371	FSIS11815242	USA	N	N	wt
108930	KKC321	USA	N	N	wt

[1] unk: an incomplete gene sequence and could not determine if it was functional; Y: present ; N: no gene

[2] unk: an incomplete gene sequence and could not determine if it was functional; Y: present ; N: no gene

[3] wt: wt *cts* genes; E: ctsE mutation ; F: ctsF mutation
