# Supplementary material for: Genomic Analysis Points to Multiple Genetic Mechanisms for Non-Transformable Campylobacter jejuni ST-50
Source: Microorganisms. 2024 Feb 4;12(2):327. doi: 10.3390/microorganisms12020327 (PMC10893306; doi:10.3390/microorganisms12020327)
Supplement: Supplementary file 1 [file microorganisms-12-00327-s001.zip › TableS5-Parker_et_al2024.pdf]

| Selected | index | ID | Cjc_cgc_10 | Cjc_cgc_100 | Cjc_cgc_200 | Cjc_cgc_25 |
|---|---|---|---|---|---|---|
| | 1 | 6310 | 239 | 66 | 23 | 217 |
| | 2 | 22255 | 1126 | 24 | 23 | 885 |
| | 3 | 30569 | 2087 | 858 | 398 | 1543 |
| | 4 | 32893 | 20 | 16 | 23 | 19 |
| | 5 | 37570 | 27 | 23 | 22 | 26 |
| | 6 | 39737 | 8589 | 858 | 398 | 2330 |
| | 7 | 40513 | 27 | 23 | 22 | 26 |
| | 8 | 43339 | 27 | 23 | 22 | 26 |
| | 9 | 48402 | 5268 | 66 | 23 | 3439 |
| | 10 | 51553 | | | | |
| | 11 | 56445 | 27 | 23 | 22 | 26 |
| | 12 | 56604 | 20 | 16 | 23 | 19 |
| | 13 | 57531 | 11727 | 24 | 23 | 3916 |
| | 14 | 57783 | 578 | 133 | 23 | 185 |
| | 15 | 60600 | | | | |
| | 16 | 61660 | 6885 | 658 | 457 | 4518 |
| | 17 | 62031 | 7719 | 24 | 23 | 3916 |
| | 18 | 62368 | 7034 | 24 | 23 | 4643 |
| | 19 | 62797 | 20 | 16 | 23 | 19 |
| | 20 | 69767 | 7552 | 120 | 100 | 2127 |
| | 21 | 69781 | 7559 | 120 | 100 | 2127 |
| | 22 | 70110 | 6509 | 66 | 23 | 4264 |
| | 23 | 70943 | 8001 | 2463 | 1504 | 5236 |
| | 24 | 76581 | 7554 | 120 | 100 | 2127 |
| | 25 | 76902 | 27 | 23 | 22 | 26 |
| | 26 | 77678 | 8711 | 24 | 23 | 5667 |
| | 27 | 79837 | 20 | 16 | 23 | 19 |
| | 28 | 80734 | 9340 | 24 | 23 | 3916 |
| | 29 | 83558 | 10516 | 24 | 23 | 3916 |
| | 30 | 84435 | 5304 | 24 | 23 | 305 |
| | 31 | 92074 | 9943 | 24 | 23 | 6497 |
| | 32 | 92223 | 13017 | 24 | 23 | 8355 |
| | 33 | 92532 | 13079 | 24 | 23 | 8397 |
| | 34 | 92583 | 13096 | 24 | 23 | 3916 |
| | 35 | 92597 | 13101 | 3161 | 1785 | 7703 |
| | 36 | 92750 | 5304 | 24 | 23 | 305 |
| | 37 | 92951 | 13186 | 24 | 23 | 3916 |
| | 38 | 92959 | 13059 | 24 | 23 | 3916 |
| | 39 | 93123 | 13209 | 24 | 23 | 3916 |
| | 40 | 93493 | 9487 | 34 | 23 | 6188 |

| | | | | | |
|---|---|---|---|---|---|
| 41 | 93623 | 13315 | 24 | 23 | 3916 |
| 42 | 93697 | 13330 | 24 | 23 | 7549 |
| 43 | 93736 | 12757 | 3312 | 23 | 8199 |
| 44 | 93773 | 13348 | 24 | 23 | 8551 |
| 45 | 98915 | 9397 | 24 | 23 | 3916 |
| 46 | 98987 | 5304 | 24 | 23 | 305 |
| 47 | 99148 | 9487 | 34 | 23 | 6188 |
| 48 | 99443 | 9673 | 24 | 23 | 6327 |
| 49 | 103252 | 13017 | 24 | 23 | 8355 |
| 50 | 104567 | 9787 | 34 | 23 | 6398 |
| 51 | 104732 | 9889 | 24 | 23 | 6465 |
| 52 | 106388 | 16571 | 24 | 23 | 10510 |
| 53 | 110094 | 17094 | 24 | 23 | 305 |
| 54 | 110642 | | | | |
| 55 | 111135 | 7985 | 24 | 23 | 5226 |
| 56 | 111508 | 17311 | 4223 | 23 | 11045 |
| 57 | 111713 | 17354 | 24 | 23 | 11074 |
| 58 | 111728 | 17354 | 24 | 23 | 11074 |
| 59 | 114671 | 342 | 24 | 23 | 305 |
| 60 | 116314 | 29860 | 24 | 23 | 19695 |
| 61 | 116315 | 29861 | 24 | 23 | 19696 |
| 62 | 116316 | 29862 | 7865 | 23 | 19697 |
| 63 | 116317 | 29863 | 7866 | 23 | 19698 |

| Cjc_cgc_5 | Cjc_cgc_50 | comments | continent | country | isolate | penner |
|---|---|---|---|---|---|---|
| 249 | 185 | | Europe | UK [England] | OXC453 | |
| 1248 | 26 | | Europe | UK [England] | OXC7071 | |
| 2425 | 1146 | | Europe | UK [England] | OXC8885 | |
| 777 | 18 | | Europe | UK [England] | OXC9837 | |
| 27 | 25 | Grey seal pup | Europe | UK [Scotland] | Seal183 | |
| 3906 | 1674 | iCaMPS3 | Europe | UK [Scotland] | B0050 | |
| 27 | 25 | iCaMPS3 | Europe | UK [Scotland] | C0788 | |
| 27 | 25 | Scottish_FSA | Europe | UK [Scotland] | C0261 | |
| 6437 | 80 | iCaMPS-4 | Europe | UK [Scotland] | ARI4066 | |
| | | meat | Europe | Spain | 11_S10 | |
| 27 | 25 | iCaMPS-4 | Europe | UK [Scotland] | ARI4473 | |
| 5478 | 18 | iCaMPS-4 | Europe | UK [Scotland] | C1585 | |
| 7410 | 2754 | crow | North Americ | USA | BCW_6872 | |
| 622 | 162 | | Europe | UK | chick50 | |
| | | | North Americ | Canada | RM3412 | |
| 8492 | 3169 | Lamb/Sheep | Europe | UK | FDI295 | |
| 8598 | 2754 | Animal-Cattle | North Americ | USA | FSIS1606748 | |
| 8654 | 26 | | Europe | France | AC0745 | |
| 777 | 18 | | Europe | UK [England] | NWC1796 | |
| 9366 | 148 | | Oceania | New Zealand | 16CC0850 | |
| 9375 | 148 | | Oceania | New Zealand | 16CC0947 | |
| 8024 | 80 | | Europe | UK | FDI452 | |
| 9921 | 3613 | | Europe | Finland | Po_1 | |
| 10374 | 148 | | Europe | UK [England] | NWC2398 | |
| 27 | 25 | | Europe | UK | FDI580 | |
| 10867 | 3864 | | Europe | UK | PHEHIST096 | |
| 777 | 18 | | Europe | UK [England] | RL16000343 | |
| 11639 | 2754 | rMLST indicat | North Americ | USA | PS00309 | |
| 13024 | 2754 | | North Americ | USA | 2016D-0258 | |
| 6480 | 26 | | North Americ | USA | 2016D-0064 | |
| 12327 | 4372 | USDA-FSIS; Su | North Americ | USA | FSIS1701236 | |
| 16220 | 5421 | USDA-FSIS; Su | North Americ | USA | FSIS1710996 | |
| 16293 | 4066 | USDA-FSIS; Su | North Americ | USA | FSIS1607853 | |
| 16314 | 2754 | USDA-FSIS; Su | North Americ | USA | FSIS1702913 | |
| 16319 | 5049 | USDA-FSIS; Su | North Americ | USA | FSIS21820901 | |
| 6480 | 26 | USDA-FSIS; Su | North Americ | USA | FSIS1609374 | |
| 16422 | 2754 | USDA-FSIS; Su | North Americ | USA | FSIS1709833 | |
| 16269 | 2754 | USDA-FSIS; Su | North Americ | USA | FSIS1609357 | |
| 16454 | 2754 | USDA-FSIS; Su | North Americ | USA | FSIS1608758 | |
| 11803 | 4188 | USDA-FSIS; Su | North Americ | USA | FSIS21720686 | |

| | | | | | | |
|---|---|---|---|---|---|---|
| 16584 | 2754 | USDA-FSIS; Su | North Americ | USA | FSIS1710700 | |
| 16602 | 2685 | USDA-FSIS; Su | North Americ | USA | FSIS1703025 | |
| 15893 | 5329 | USDA-FSIS; Su | North Americ | USA | FSIS11705500 | |
| 16622 | 4202 | USDA-FSIS; Su | North Americ | USA | FSIS21720655 | |
| 15000 | 2754 | USDA-FSIS; Su | North Americ | USA | FSIS11812592 | |
| 6480 | 26 | USDA-FSIS; Su | North Americ | USA | FSIS11812063 | |
| 11803 | 4188 | USDA-FSIS; Su | North Americ | USA | FSIS11811270 | |
| 17865 | 4272 | USDA-FSIS; Su | North Americ | USA | FSIS1607146 | |
| 16220 | 5421 | USDA-FSIS; Su | North Americ | USA | FSIS1701497 | |
| 12149 | 4188 | USDA-FSIS; Su | North Americ | USA | FSIS11918239 | |
| 12262 | 2754 | USDA-FSIS; Su | North Americ | USA | FSIS11917669 | |
| 20787 | 6667 | | North Americ | Canada | RM3405 | |
| 21416 | 26 | | Europe | Luxembourg | LNS9143194 | |
| | | | Asia | Japan | 1753 | |
| 10420 | 26 | | Europe | Portugal | Cj-4207 | |
| 21681 | 7034 | | Asia | South Korea | CS41 | |
| 21731 | 7057 | | North Americ | Canada | R1S3-12 | |
| 21731 | 7057 | | North Americ | Canada | R1S3-W2A | |
| 27885 | 26 | rMLST=Excluc | Europe | France | bigsid_8896 | |
| 37115 | 12956 | | Europe | Italy | RM5146 | HS:8 |
| 37116 | 26 | | Europe | Italy | RM5148 | HS:8 |
| 37117 | 12957 | | Europe | Italy | RM5149 | HS:1 |
| 37118 | 12958 | | Europe | Italy | RM5156 | HS:1 |

| region | source | town or city | year |
|---|---|---|---|
| Oxfordshire | human stool | | 2004 |
| Oxfordshire | human stool | | 2012 |
| Oxfordshire | human stool | | 2014 |
| Oxfordshire | human stool | | 2015 |
| | other animal | | 2011 |
| | cattle | Aberdeen | 2013 |
| | turkey | Aberdeen | 2013 |
| | chicken | Aberdeen | 2011 |
| | human stool | Aberdeen | 2015 |
| | chicken | Madrid | 2010 |
| | human stool | Aberdeen | 2016 |
| | chicken | Aberdeen | 2016 |
| | wild bird | | |
| | chicken | | 2009 |
| | human unspecified | | 1980 |
| | lamb offal or meat | | 2017 |
| PA | cattle | | 2016 |
| | chicken | | |
| Newcastle/N | human stool | | 2017 |
| Hawke's Bay | human stool | | 2016 |
| Hawke's Bay | human stool | | 2016 |
| | beef offal or meat | | 2018 |
| | human stool | | 2002 |
| Newcastle/N | human stool | | 2018 |
| | chicken offal or meat | | 2018 |
| | human stool | | 1997 |
| | chicken offal or meat | | 2016 |
| PA | human unspecified | | 2018 |
| WA | chicken | | 2017 |
| IL | chicken offal or meat | | 2017 |
| WI | chicken offal or meat | | 2016 |
| TX | chicken offal or meat | | 2017 |
| SC | chicken offal or meat | | 2017 |
| GA | chicken offal or meat | | 2016 |
| AL | chicken offal or meat | | 2016 |
| FL | chicken | | 2016 |
| GA | chicken | | 2016 |
| GA | chicken offal or meat | | 2017 |

| | | |
|---|---|---|
| FL | chicken | 2017 |
| IN | chicken offal or meat | 2017 |
| LA | chicken | 2017 |
| AL | chicken offal or meat | 2017 |
| KS | cattle | 2018 |
| DE | chicken | 2018 |
| SC | chicken | 2018 |
| NE | cattle | 2016 |
| MD | cattle | 2017 |
| KS | pig | 2019 |
| MO | pig | 2019 |
| | | |
| | human stool | 2021 |
| | chicken offal or meat | 2020 |
| | human stool | 2021 |
| | chicken | 2017 |
| British Colum | broiler environment | 2020 |
| British Colum | chicken offal or meat | 2020 |
| | human stool | 2016 |
| | turkey | |
| | turkey | |
| | chicken | |
| | human unspecified | |

Table S5. *Campylobacter jejuni* ST-50 strains from North America and Europe from PubMLST to create Figure 2. Neighbor-joining dendrogram.

| PubMLST ID | strain | continent | country | region | source |
|---|---|---|---|---|---|
| 6310 | OXC453 | Europe | UK | Oxfordshire | human |
| 22255 | OXC7071 | Europe | UK | Oxfordshire | human |
| 30569 | OXC8885 | Europe | UK | Oxfordshire | human |
| 32893 | OXC9837 | Europe | UK | Oxfordshire | human |
| 37570 | Seal183 | Europe | UK | | seal |
| 39737 | B0050 | Europe | UK | | cattle |
| 40513 | C0788 | Europe | UK | | turkey |
| 43339 | C0261 | Europe | UK | | chicken |
| 48402 | ARI4066 | Europe | UK | | human |
| 51553 | 11_S10 | Europe | Spain | | chicken |
| 56445 | ARI4473 | Europe | UK | | human |
| 56604 | C1585 | Europe | UK | | chicken |
| 57531 | BCW_6872 | North America | USA | CA | wild bird |
| 57783 | chick50 | Europe | UK | | chicken |
| 60600 | RM3412 | North America | Canada | | human |
| 61660 | FDI295 | Europe | UK | | lamb |
| 62031 | FSIS1606748 | North America | USA | PA | cattle |
| 62368 | AC0745 | Europe | France | | chicken |
| 62797 | NWC1796 | Europe | UK | Newcastle/North Tyneside | human |
| 69767 | 16CC0850 | Oceania | NZ | Hawke's Bay | human |
| 69781 | 16CC0947 | Oceania | NZ | Hawke's Bay | human |
| 70110 | FDI452 | Europe | UK | | beef |
| 70943 | Po_1 | Europe | Finland | | human |
| 76581 | NWC2398 | Europe | UK | Newcastle/North Tyneside | human |
| 76902 | FDI580 | Europe | UK | | chicken |
| 77678 | PHEHIST096 | Europe | UK | | human |
| 79837 | RL16000343 | Europe | UK | | chicken |
| 80734 | PS00309 | North America | USA | PA | human |
| 83558 | 2016D-0258 | North America | USA | | human |
| 84435 | 2016D-0064 | North America | USA | | human |
| 92074 | FSIS1701236 | North America | USA | WA | chicken |
| 92223 | FSIS1710996 | North America | USA | IL | chicken |
| 92532 | FSIS1607853 | North America | USA | WI | chicken |
| 92583 | FSIS1702913 | North America | USA | TX | chicken |
| 92597 | FSIS21820901 | North America | USA | SC | chicken |
| 92750 | FSIS1609374 | North America | USA | GA | chicken |
| 92951 | FSIS1709833 | North America | USA | AL | chicken |
| 92959 | FSIS1609357 | North America | USA | FL | chicken |
| 93123 | FSIS1608758 | North America | USA | GA | chicken |

| | | | | | |
|---|---|---|---|---|---|
| 93493 | FSIS21720686 | North America | USA | GA | chicken |
| 93623 | FSIS1710700 | North America | USA | FL | chicken |
| 93697 | FSIS1703025 | North America | USA | IN | chicken |
| 93736 | FSIS11705500 | North America | USA | LA | chicken |
| 93773 | FSIS21720655 | North America | USA | AL | chicken |
| 98915 | FSIS11812592 | North America | USA | KS | cattle |
| 98987 | FSIS11812063 | North America | USA | DE | chicken |
| 99148 | FSIS11811270 | North America | USA | SC | chicken |
| 99443 | FSIS1607146 | North America | USA | NE | cattle |
| 103252 | FSIS1701497 | North America | USA | MD | cattle |
| 104567 | FSIS11918239 | North America | USA | KS | pig |
| 104732 | FSIS11917669 | North America | USA | MO | pig |
| 106388 | RM3405 | North America | Canada | | human |
| 110094 | LNS9143194 | Europe | Luxembrg. | | human |
| 110642 | 1753 | Asia | Japan | | chicken |
| 111135 | Cj-4207 | Europe | Portugal | | human |
| 111508 | CS41 | Asia | S. Korea | | chicken |
| 111713 | R1S3-12 | North America | Canada | British Columbia | broiler enviro. |
| 111728 | R1S3-W2A | North America | Canada | British Columbia | chicken |
| 114671 | bigsid_8896 | Europe | France | | human |
| 116314 | RM5146 | Europe | Italy | | turkey |
| 116315 | RM5148 | Europe | Italy | | turkey |
| 116316 | RM5149 | Europe | Italy | | chicken |
| 116317 | RM5156 | Europe | Italy | | human |

used

| year |
|---|
| 2004 |
| 2012 |
| 2014 |
| 2015 |
| 2011 |
| 2013 |
| 2013 |
| 2011 |
| 2015 |
| 2010 |
| 2016 |
| 2016 |
| unk |
| 2009 |
| 1979 |
| 2017 |
| 2016 |
| unk |
| 2017 |
| 2016 |
| 2016 |
| 2018 |
| 2002 |
| 2018 |
| 2018 |
| 1997 |
| 2016 |
| 2018 |
| 2016 |
| 2016 |
| 2017 |
| 2017 |
| 2016 |
| 2017 |
| 2017 |
| 2016 |
| 2016 |
| 2016 |
| 2016 |

| |
|---|
| 2017 |
| 2017 |
| 2017 |
| 2017 |
| 2017 |
| 2018 |
| 2018 |
| 2018 |
| 2016 |
| 2017 |
| 2019 |
| 2019 |
| 1980 |
| 2021 |
| 2020 |
| 2021 |
| 2017 |
| 2020 |
| 2020 |
| 2016 |
| unk |
| unk |
| unk |
| unk |
